# Oceanic dispersal barriers in a holoplanktonic gastropod

**DOI:** 10.1111/jeb.13735

**Published:** 2020-11-21

**Authors:** Le Qin Choo, Thijs M. P. Bal, Erica Goetze, Katja T. C. A. Peijnenburg

**Affiliations:** ^1^ Plankton Diversity and Evolution Naturalis Biodiversity Center Leiden The Netherlands; ^2^ Department of Freshwater and Marine Ecology Institute for Biodiversity and Ecosystem Dynamics University of Amsterdam Amsterdam The Netherlands; ^3^ Faculty of Biosciences and Aquaculture Nord University Bodø Norway; ^4^ Department of Oceanography University of Hawaiʻi at Mānoa Honolulu USA

**Keywords:** Atlantic Ocean, geometric morphometrics, phylogeography, shelled pteropods, zooplankton

## Abstract

Pteropods, a group of holoplanktonic gastropods, are regarded as bioindicators of the effects of ocean acidification on open ocean ecosystems, because their thin aragonitic shells are susceptible to dissolution. While there have been recent efforts to address their capacity for physiological acclimation, it is also important to gain predictive understanding of their ability to adapt to future ocean conditions. However, little is known about the levels of genetic variation and large‐scale population structuring of pteropods, key characteristics enabling local adaptation. We examined the spatial distribution of genetic diversity in the mitochondrial cytochrome *c* oxidase I (COI) and nuclear 28S gene fragments, as well as shell shape variation, across a latitudinal transect in the Atlantic Ocean (35°N–36°S) for the pteropod *Limacina bulimoides*. We observed high levels of genetic variability (COI *π* = 0.034, 28S *π* = 0.0021) and strong spatial structuring (COI Φ_ST_ = 0.230, 28S Φ_ST_ = 0.255) across this transect. Based on the congruence of mitochondrial and nuclear differentiation, as well as differences in shell shape, we identified a primary dispersal barrier in the southern Atlantic subtropical gyre (15–18°S). This barrier is maintained despite the presence of expatriates, a gyral current system, and in the absence of any distinct oceanographic gradients in this region, suggesting that reproductive isolation between these populations must be strong. A secondary dispersal barrier supported only by 28S pairwise Φ_ST_ comparisons was identified in the equatorial upwelling region (between 15°N and 4°S), which is concordant with barriers observed in other zooplankton species. Both oceanic dispersal barriers were congruent with regions of low abundance reported for a similar basin‐scale transect that was sampled 2 years later. Our finding supports the hypothesis that low abundance indicates areas of suboptimal habitat that result in barriers to gene flow in widely distributed zooplankton species. Such species may in fact consist of several populations or (sub)species that are adapted to local environmental conditions, limiting their potential for adaptive responses to ocean changes. Future analyses of genome‐wide diversity in pteropods could provide further insight into the strength, formation and maintenance of oceanic dispersal barriers.

## Graphical abstract

Shelled pteropods or 'sea butterflies' are proposed as bio‐indicators to monitor impacts of ocean acidification on open ocean ecosystems. Local adaptation is affected by the presence and geographic extent of distinct populations, however, little is known about the large‐scale population structure of pteropods. We thus examined the spatial distribution of genetic and morphometric variation in the species *Limacina bulimoides* across the Atlantic Ocean. Two dispersal barriers were identified that were congruent with regions of low abundance, suggesting that regions of suboptimal habitat may create barriers to gene flow in widely‐distributed zooplankton species.

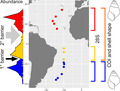

## 1 INTRODUCTION

Anthropogenic carbon emissions are an important cause of perturbations in marine ecosystems, including an overall increase in seawater temperature, ocean deoxygenation and acidification (Gattuso et al., 2015; Gruber et al., 2019). These perturbations are expected to have wide‐ranging impacts on marine organisms and may drive species range shifts and ecosystem regime shifts due to climate‐driven invasions and extinctions (Doney et al., 2012; Kroeker et al., 2013; Poloczanska et al., 2016). It is uncertain whether marine organisms have the short‐term plasticity and/or long‐term evolutionary potential to adapt, given the ongoing and expected rates of environmental change (Donelson et al., 2019; Miller et al., 2018). To gain insight into the evolutionary potential of marine taxa, it is necessary to resolve their underlying genetic variation, population structure and connectivity (Bell, 2013; Harvey et al., 2014; Munday et al., 2013; Poloczanska et al., 2016; Sunday et al., 2014).

Locally adapted populations are increasingly reported in marine systems, showing that the apparent connectivity of the marine environment is often not fully realized by organisms, and populations may be more spatially constrained than once thought (Barth et al., 2017; Nielsen et al., 2009; Sanford & Kelly, 2011). Several case studies have illuminated the processes involved in local adaptation of marine molluscs, for example habitat preference and partial spawning asynchrony in the mussels *Mytilus edulis* and *Mytilus galloprovincialis* (Bierne et al., 2003), ecological selection across microhabitats in the ecotypes of the rocky shore gastropod *Littorina saxatilis* (Butlin et al., 2014; Johannesson et al., 2017; Westram et al., 2018), or variation in thermal stress tolerance in the intertidal gastropod *Chlorostoma funebralis* (Gleason & Burton, 2016). Much of the existing literature has focused on dispersal barriers in marine benthic taxa, some of which have pelagic larval stages. However, little is known about evolutionary pressures acting in the pelagic environment. One reason is that it is difficult to differentiate between selection pressures acting on the larval pelagic and adult benthic life stages on the same genome (Marshall & Morgan, 2011). Thus, it could be useful to study wholly pelagic organisms, such as holozooplankton, to better understand the selective forces acting upon organisms in the open ocean.

Population structure in marine holozooplankton appears to be more influenced by their ability to establish and maintain viable populations outside of their core range, rather than dispersal limitations (De Vargas et al., 1999; Norton & Goetze, 2013; Peijnenburg et al., 2004). Given the large population sizes, extensive distribution patterns, high standing genetic diversity, and short generation times of marine zooplankton in general, adaptive responses to even weak selection may be expected as the loss of variation due to genetic drift should be negligible (Peijnenburg & Goetze, 2013). While some plankton communities show rapid range shifts and phenological changes in response to ocean warming (Beaugrand et al., 2009, 2014; Hays et al., 2005), we only have limited understanding of the potential for evolutionary responses of marine zooplankton to future conditions (Dam, 2013; Peijnenburg & Goetze, 2013).

Pteropods are a group of holoplanktonic gastropods that play important roles in planktonic food webs and ocean biogeochemistry, due to their high abundance and production of calcium carbonate shells (Bé & Gilmer, 1977; Bednaršek et al., 2012; Buitenhuis et al., 2019; Hunt et al., 2008). Shelled pteropods are especially vulnerable to global change, because of their thin aragonitic shells that are prone to dissolution (Bednaršek, Tarling, et al., 2012; Lischka et al., 2011; Manno et al., 2017), and they have been suggested as bioindicators of the effects of ocean acidification (Bednaršek, Klinger, et al., 2017). Several prior genetic studies on shelled pteropods have focused on resolving species boundaries via an integrative approach, combining genetic analyses and morphometric measurements of shells, to obtain a better understanding of species distribution patterns (Burridge et al., 2015, 2019; Shimizu et al., 2018). Other recent research efforts have addressed the response of shelled pteropods to acidified conditions to evaluate their acclimation to rapid increases in ocean acidity (Bednaršek, Feely, et al., 2017; Bogan et al., 2020; Maas et al., 2018; Moya et al., 2016). Little is known, however, about the spatial distribution of natural genetic and phenotypic variability in pteropod species with widespread distributions.

Planktonic ecosystems in the Atlantic Ocean have been relatively well‐sampled owing to spatially extensive (~13,500 km) annual transects of the Atlantic Meridional Transect programme (AMT, https://www.amt‐uk.org), and are thus ideal for studying population structure and dispersal barriers in marine zooplankton. The AMT cruises traverse both the northern and southern subtropical gyres, which are separated by the equatorial upwelling region. The subtropical gyres are oligotrophic systems characterized by clear ocean waters with very low primary productivity in the surface layer, high sea surface temperature, a deep thermocline, and the presence of a deep chlorophyll maximum. Conversely, the mesotrophic equatorial province is characterized by upwelling of nutrient‐rich deep waters that stimulate primary production. Several species of copepods show spatial genetic structuring congruent with these Atlantic oceanic provinces. For example, *Pleuromamma abdominalis* has clades that are endemic to the equatorial province (Hirai et al., 2015). Other mesopelagic copepods, such as *Haloptilus longicornis* (Andrews et al., 2014; Norton & Goetze, 2013) and *Pleuromamma xiphias* (Goetze et al., 2017) show strong genetic breaks between the northern and southern subtropical gyre populations. These dispersal barriers coincide with regions of low abundance, which may represent areas of suboptimal habitat, supporting an ecological basis for these barriers (Goetze et al., 2015, 2017). However, it is unclear whether other zooplankton groups show similar patterns of basin‐scale genetic structuring. For holoplanktonic gastropods, DNA barcoding has revealed intra‐specific genetic differentiation between and within ocean basins, pointing towards undescribed diversity (Burridge, Hörnlein, et al., 2017; Jennings et al., 2010; Wall‐Palmer et al., 2018). Using DNA barcoding, two closely related species of *Protatlanta* (a genus of Atlantidae, commonly known as shelled heteropods) were identified in the Atlantic Ocean: *P. souleyeti*, which is abundant in the subtropical gyres, and *P. sculpta*, which is most abundant in the equatorial upwelling region (Wall‐Palmer et al., 2016). There is also some evidence that populations of the straight‐shelled pteropod *Cuvierina atlantica* show genetic discontinuity across the equatorial upwelling region in the Atlantic Ocean (Burridge et al., 2015).

In this study, we aim to identify barriers to dispersal and spatial population structure in the coiled‐shelled pteropod *L. bulimoides* (d'Orbigny, 1835) across a latitudinal transect in the Atlantic Ocean. This species has a circumglobal warm‐water distribution from ~45°N to ~40°S, a preferred depth range of 80–120 m, and it performs diel vertical migration with higher abundance in surface waters at night (Bé & Gilmer, 1977). In the Atlantic basin, the species is common across several ocean provinces with peaks in abundance in the oligotrophic gyres (Bé & Gilmer, 1977; Burridge, Goetze, et al., 2017). By assessing the distribution of genetic and phenotypic variability in *L. bulimoides* across a latitudinal Atlantic transect, we aim to test the hypothesis that areas of low abundance mark regions of suboptimal habitat that represent barriers to gene flow. To do this, we sequenced fragments of the mitochondrial cytochrome *c* oxidase I (COI) and the nuclear 28S genes and made geometric morphometric measurements of shell shape. Our objectives were to (a) identify the location of dispersal barriers based on two genetic markers, (b) test for congruence of genetic barriers with differences in shell shape, and (c) assess whether the spatial structure was better explained by shifts in abundance or oceanographic parameters. By identifying dispersal barriers and the possible drivers of population structure in holoplanktonic gastropods, we can better predict their capacity to respond to a rapidly acidifying ocean.

## 2 MATERIALS AND METHODS

### 2.1 Species ecology and biology

*Limacina bulimoides* is a sinistrally coiled holoplanktonic gastropod, well‐adapted to life in the open ocean. It has a small (maximum shell length = 2 mm, van der Spoel et al., 1997), thin, aragonitic shell and two parapodia, which it uses to swim through the water column in analogous fashion to how insects fly through the air—a remarkable example of convergent evolution (Murphy et al., 2016). For feeding, an external mucous web traps particulate matter, including phytoplankton and small protists, which is brought to the mouth by ciliary movement (Lalli & Gilmer, 1989). The radula, a typical molluscan structure used for feeding, has been described from a congener, *Limacina helicina*, and was reduced, with 10 rows of teeth (Gilmer & Harbison, 1986; Lalli & Gilmer, 1989; Meisenheimer, 1905; Ritcher, 1979). However, in a recent detailed morphological study of the same species, only six rows of teeth were found, which led the authors to suggest that the number of rows could be variable due to continuous regrowth (Laibl et al., 2019). *Limacina bulimoides* lay free‐floating egg strings (see Figure S1/Video S1), from which free‐swimming veliger larvae hatch (Lalli & Wells, 1978). *Limacina* spp. are protandrous hermaphrodites, hence, the larvae sexually mature into males before transitioning into females (Lalli & Gilmer, 1989). The generation time of *L. bulimoides* is estimated to be less than a year, with larvae metamorphosing into juveniles after 2 months, juveniles reaching sexual maturity as males after a subsequent 5 months, and reaching their maximum size as females 2 months later (Wells, 1976). Reciprocal copulation has been observed between males (Morton, 1954), or between individuals that are in transition between male and female (Lalli & Gilmer, 1989). They possess a penis for internal fertilization (Lalli & Wells, 1978), and it is unknown whether self‐fertilization is possible.

### 2.2 Sampling

**FIGURE 1:**
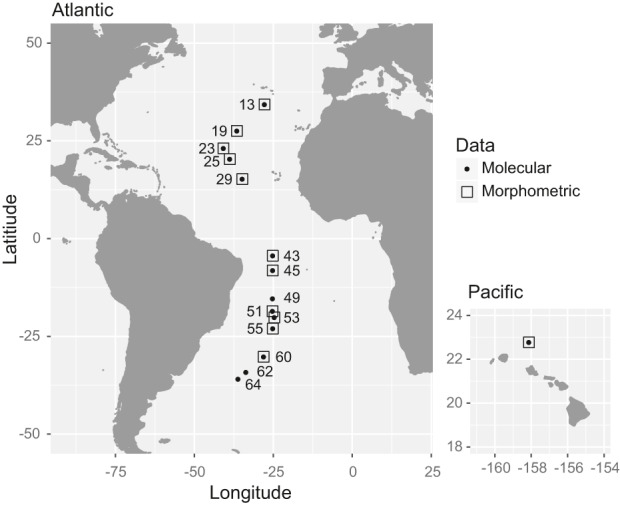
Sampling locations of *Limacina bulimoides* along the basin‐scale Atlantic Meridional Transect (AMT22, left), as well as in the Pacific Ocean off Hawaiʻi (right). Numbers indicate the station number for each site, and symbols indicate the type of data that were obtained (see legend)

Bulk plankton samples were collected on AMT cruise 22 (AMT22) in October and November 2012 (Table 1, Figure 1). The samples were collected at night by oblique tows of a bongo net (200 and 333 µm mesh sizes) or RMT1 midwater trawl (333 µm) from a median depth of 323 m to the sea surface (depth range: 132–402 m). Samples were preserved in 95% ethanol, stored at −20°C, and subsequently sorted in the laboratory. Seawater temperature and chlorophyll *a* concentration in the upper 300 m of the water column were obtained using a Sea‐Bird Electronics 3P Temperature Sensor and Chelsea MKIII Aquatracka Fluorometer, with data calibrated and archived by the British Oceanographic Data Centre (BODC, https://www.bodc.ac.uk). The 14 samples included in this study were collected between 35°N and 36°S in the following Longhurst biogeochemical provinces (Longhurst, 2007; Reygondeau et al., 2013): North Atlantic Subtropical gyre (NAST), North Atlantic Tropical gyre (NATR), Western Tropical Atlantic (WTRA), South Atlantic gyre (SATL), South Subtropical Convergence (SSTL) (Table 1). Additionally, one sample site of *L. bulimoides* from the Pacific was included as an outgroup to provide a broader perspective on the diversity found for the Atlantic individuals (Table 1, Figure 1). For population‐level analyses, the Longhurst province assignments were simplified to four major ocean provinces (North Gyre, Equatorial, South Gyre and Convergence) (Table 1), which also take into account previously reported transitions in planktonic community composition along the AMT transects (Burridge, Goetze, et al., 2017; Burridge et al., 2017; Goetze et al., 2017).

**TABLE 1:** Sampling overview for *Limacina bulimoides* from the Atlantic AMT22 (14 stations) and Pacific KH11‐10 (1 station) cruises, including the location, date collected, Longhurst province assignment of the stations, number of individuals sampled for mitochondrial cytochrome *c* oxidase I (COI), nuclear 28S (28S) genes, and morphometric data

Cruise & Station	Latitude	Longitude	Date	Longhurst code	COI	28S	Morphometrics	Ocean provinces	Population groups
AMT22_13	34°22′N	27°38′W	18/10/2012	NAST/NATR	28	28	10	North Gyre	North
AMT22_19	27°36′N	36°22′W	21/10/2012	NATR	31	26	17	North Gyre	North
AMT22_23	23°9′N	40°38′W	23/10/2012	NATR	28	24	2	North Gyre	North
AMT22_25	20°24′N	38°37′W	24/10/2012	NATR	19	19	21	North Gyre	North
AMT22_29A	15°18′N	34°40′W	26/10/2012	NATR/WTRA	21	19	20	Equatorial	North
AMT22_43A	4°19′S	25°1′W	1/11/2012	WTRA	25	20	3	Equatorial	Equatorial
AMT22_45	8°5′S	25°2′W	3/11/2012	WTRA/SATL	41	41	6	Equatorial	Equatorial
AMT22_49	15°18′S	25°4′W	5/11/2012	SATL	27	31	‐	South Gyre	Equatorial
AMT22_51	18°30′S	25°6′W	6/11/2012	SATL	16	16	1	South Gyre	South
AMT22_53	20°6′S	24°31′W	6/11/2012	SATL	25	25	17	South Gyre	South
AMT22_55	22°57′S	25°0′W	8/11/2012	SATL	38	38	6	South Gyre	South
AMT22_60	30°10′S	27°54′W	12/11/2012	SATL/SSTC	24	28	8	Convergence	South
AMT22_62	34°7′S	33°30′W	14/11/2012	SATL/SSTC	8	7	‐	Convergence	South
AMT22_64A	35°52′S	36°0′W	14/11/2012	SATL/SSTC	3	2	‐	Convergence	South
KH1110_08	22°47′N	158°06′W	19/12/2011	NPTG	22	20	25	–	–
Total					356	344	136		

Note

The Longhurst codes mentioned in the table are as follows: NAST = North Atlantic Subtropical gyre, NATR = North Atlantic Tropical gyre, WTRA = Western Tropical Atlantic, SATL = South Atlantic gyre, SSTC = South Subtropical Convergence, NPTG = North Pacific Tropical gyre. Sites with two Longhurst province assignments indicate transitional zones. A division of sampling sites into groups representing four major ocean provinces and three groups based on abundance along a similar transect are indicated in the last two columns, respectively.

Abundance of *L. bulimoides* was obtained from AMT24 (September–October 2014) as quantitative samples from AMT22 were not available. For this equivalent AMT transect, bulk plankton samples were collected using a bongo net (200 µm mesh size) with a General Oceanics flowmeter (2030RC) mounted in the mouth to quantify the volume of seawater filtered (see Burridge, Hörnlein, et al., 2017). Based on the peaks in abundance of *L. bulimoides* across the AMT24 transect and comparison of oceanographic measurements from both the AMT22 and AMT24 transects, the sampled stations were split into three ‘population groups’ (North, Equatorial, South) (Table 1).

### 2.3 Molecular analyses

To resolve spatial genetic structuring of *L. bulimoides*, 356 individuals were sequenced for a 565 base pair (bp) fragment of the mitochondrial COI gene, and 344 individuals for a 938 bp fragment of the nuclear 28S gene (Table 1). In total, 332 *L. bulimoides* specimens were sequenced for both COI and 28S (Table S1). The average number of individuals sampled per station for COI and 28S was 24 and 23, respectively. Individuals from stations 62 and 64 were combined because of their geographic proximity and low numbers of specimens. DNA was extracted with either the NucleoMag 96 Tissue Kit (Macherey‐Nagel GmbH & Co. KG) or the DNeasy 96 Blood & Tissue Kit (Qiagen). DNA extracts were amplified in polymerase chain reaction (PCR) using the primers jgLCO1490 (5′‐TITCIACIAAYCAYAARGAYATTGG‐3′) and jgHCO2198 (5′‐TAIACYTCIGGRTGICCRAARAAYCA‐3′) for COI (Geller et al., 2013), and for 28S using C1‐F (5′‐ACCCGCTGAATTTAAGCAT‐3′) (Dayrat et al., 2001) and D3‐R (5′‐GACGATTCGATTTGCACGTCA‐3′) (Vonnemann et al., 2005). PCR was carried out with a reaction mix consisting of 1 μl template DNA, 17.8 μl milli‐Q H_2_O (Ultrapure), 2.5 μl PCR buffer CL (10X) (Qiagen), 0.5 μl MgCL_2_ (25 mM) (Qiagen), 0.5 μl BSA (100 mM) (Promega), 1 μl of each primer (10 μM), 0.5 μl dNTPs (2.5 mM) and 0.25 μl Taq polymerase (5 U/μl) (Qiagen). The initial denaturation step of 3 min at 96°C was followed by 40 cycles of 15 s at 96°C, 30 s at 50°C and 40 s at 72°C, ending with a final extension step of 5 min at 72°C. Amplified products were sequenced in both directions at Baseclear B.V. (Leiden, the Netherlands), combined and checked for errors in Geneious v8.1.9 (Kearse et al., 2012) and the final sequences were aligned using MAFFT v7.017 (Katoh & Standley, 2013).

Haplotype (*H*_d_) and nucleotide diversity (*π*) were calculated using DnaSP v6.12.01 (Rozas et al., 2017) and reported for each station for both COI and 28S fragments. The 28S data were phased in DnaSP using PHASE (Stephens & Donnelly, 2003) with default settings of 100 iterations, thinning interval of 1 and burn‐in of 100. Stations were tested for adherence to neutrality assumptions, with Tajima's *D* (Tajima, 1989) calculated in Arlequin version 3.5.2.2 (Excoffier & Lischer, 2010). Minimum‐spanning networks for both genes were calculated and visualized in POPART (Leigh & Bryant, 2015). The phased allele alignment was used to construct the 28S haplotype network.

To identify population subdivision, genetic diversity between populations from different stations was quantified by pairwise Φ_ST_ (Holsinger & Weir, 2009), using the pairwise difference method in Arlequin with 10,000 permutations, and significant comparisons were identified after strict Bonferroni correction (Rice, 1989). Hierarchical Analysis of Molecular Variance (AMOVA) tests were conducted for both genes separately with sampling sites partitioned into (a) four 'ocean province' groups and (b) three abundance‐based 'population' groups (Table 1). To test for isolation‐by‐distance (IBD), Mantel tests with 10,000 permutations were conducted in Arlequin to assess correlation between the COI or 28S pairwise Φ_ST_ matrices and the geographic distance matrix. Pairwise geographic distance between stations was calculated using the R package geodist with geodesic measures (Padgham & Sumner, 2020). The distance between stations 62 and 64 was averaged since they were analysed as a single site. The pairwise scatter plots were produced in R with ggplot2 (Wickham, 2016).

### 2.4 Morphometric analyses

Variation in shell shape was assessed using geometric morphometric measurements of 136 undamaged adult shells (shell length > 0.9 mm). Specimens were positioned in a standardized apertural orientation and photographed using a Zeiss V20 stacking stereomicroscope with Axiovision software, prior to destructive DNA extraction. The resulting images were processed and digitized at eight (semi‐)landmarks in TpsUtil and TpsDig (Rohlf, 2015) (Figure S2). The coordinates of the (semi‐)landmarks were analysed in TpsRelW (Rohlf, 2015), using a generalized least‐squares Procrustes superimposition (Rohlf & Slice, 1990; Zelditch et al., 2004). To ensure that the digitization process was repeatable, a subset of 30 individuals was photographed and digitized twice, with eight landmarks placed on each image. Centroid size and relative warp (RW) scores between the pairs of images per specimen after Procrustes Fit were compared using intra‐class coefficient (ICC) in PAST3.0 (Hammer et al., 2001), and ICC values > 0.75 were considered sufficiently repeatable.

We tested for significant variation in shell shape across genetically distinct groups with a nonparametric Permutational Multivariate Analysis of Variance (one‐way PERMANOVA) using Euclidean distances and 9,999 permutations in R with vegan (Oksanen et al., 2019). Only centroid size and repeatable RWs were used in the one‐way PERMANOVA, and strict Bonferroni corrections were applied for the pairwise PERMANOVAs. The first two RW axes were plotted to visualize shell shape variation for different groups of *L. bulimoides*. Additionally, a Canonical Variates Analysis (CVA) was conducted in R (R Core Team, 2017) to discriminate shell morphometric differences between groups. A one‐way ANOVA with a post hoc Tukey HSD test was also conducted in R to test if the means of the canonical variate for each group were different from the other groups.

## 3 RESULTS

### 3.1 Genetic variability

**FIGURE 2:**
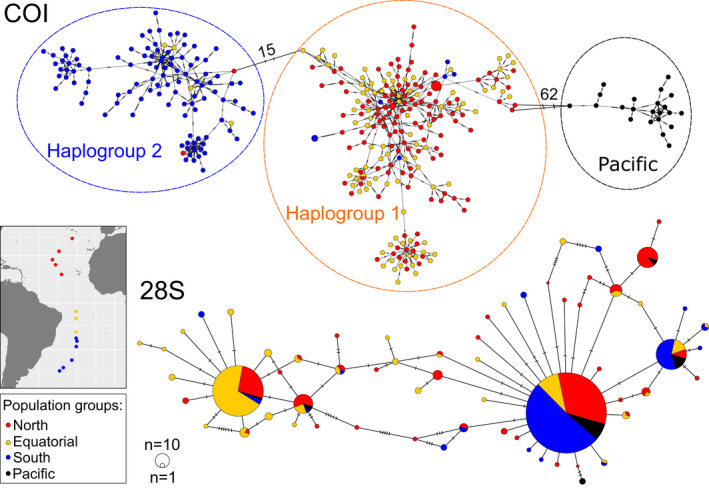
Minimum‐spanning networks of *Limacina bulimoides* gene fragments of mitochondrial cytochrome *c* oxidase I (COI, top) and nuclear 28S rDNA (28S, bottom). The size of the filled circles represents the number of individuals with each haplotype (COI, 28S), with the smallest circles representing one individual with that haplotype, while colour represents abundance‐based population groups (see map insert and Table 1). For the COI network, the dashed lines around the haplotype clusters indicate the three main haplogroups. Hatch marks on the branches represent the number of mutational steps, with large numbers of mutations between the haplogroups indicated with numbers

The COI locus was highly polymorphic with global haplotype diversity (*H*_d_) of 0.9998 (range: 0.9820–1.000) and nucleotide diversity (*π*) of 0.05449 (range: 0.0283–0.0402) across all sites (Table 2). In the COI minimum‐spanning network, 349 unique haplotypes were observed across 356 individuals, and there was no shared central haplotype (Figure 2). Instead, Atlantic individuals were clustered into two main haplogroups that were separated by 15 substitutions (haplogroups 1 & 2). A third cluster was comprised of all Pacific individuals, which was separated from Atlantic haplogroup 1 by 62 substitutions. Population samples from stations 13, 19, 25, 43 and 45 showed deviations from neutrality based on Tajima's *D* (Table 2).

**TABLE 2:** Diversity indices of *Limacina bulimoides* for both mitochondrial cytochrome *c* oxidase I (COI) and nuclear 28S (28S) genes, including haplotype diversity (*H*_d_), nucleotide diversity (*π*), and Tajima's *D* (*D*)

Station	Population groups	COI			28S		
		*H*_d_	*π*	*D*	*H*_d_	*π*	*D*
13	North	1	0.0283	−1.65*	0.623	0.0019	−1.12
19	North	1	0.0322	−1.47*	0.698	0.0028	0.359
23	North	1	0.0295	−1.44	0.855	0.0035	−0.517
25	North	0.982	0.0315	−1.57*	0.735	0.0024	−0.475
29	North	1	0.0299	−1.57	0.723	0.0031	1.46
43	Equatorial	1	0.0285	−1.53*	0.847	0.0036	−0.0628
45	Equatorial	1	0.0298	−1.46*	0.666	0.0024	−0.486
49	Equatorial	1	0.0379	−1.30	0.729	0.0032	−0.291
51	South	1	0.0356	−1.26	0.474	0.0011	−0.877
53	South	1	0.0372	−1.16	0.433	0.0006	−0.884
55	South	1	0.0402	−1.30	0.374	0.0007	−1.50*
60	South	1	0.0399	−1.19	0.458	0.0014	−1.10
62, 64	South	1	0.0382	−1.03	0.627	0.0010	−0.970
Pacific		1	0.0323	−1.33	0.818	0.0021	−0.728
**Total**		1	0.0545	−1.37	0.674	0.0027	−0.514

Note

Significant Tajima's *D* values (*p* < .05), are indicated by *. Individuals from stations 62 and 64 were combined and analysed as one station due to the low numbers of individuals per site.

The 28S gene was less polymorphic than COI, with a global *H*_d_ of 0.6740 (range: 0.3740–0.8550) and a *π* of 0.0027 (range: 0.0006–0.0035) across all sites (Table 2). This reduced polymorphism is visible in the 28S minimum‐spanning network (Figure 2), with only 63 haplotypes observed across 344 individuals. The three most abundant haplotypes comprised 369, 129 and 45 copies, respectively, and included representatives from both Atlantic and Pacific samples. The second most abundant haplotype was mainly comprised of individuals sampled from the equatorial upwelling region. Only station 55 showed a deviation from neutrality based on Tajima's *D* (Table 2).

### 3.2 Spatial structure

Overall Φ_ST_ calculated for both genes across the Atlantic supports the inference of significant spatial population structure (COI Φ_ST_ = 0.230, *p* = 0.00000; 28S Φ_ST_ = 0.255 *p* = 0.00000). Pairwise Φ_ST_ values for COI ranged from −0.017 to 0.451 for comparisons along the Atlantic transect and from 0.724 to 0.783 for comparisons between Atlantic and Pacific samples (Table 3). All comparisons between stations 13–49 (North & Equatorial) and stations 51–64 (South) were significant, with high Φ_ST_ values ranging from 0.250 to 0.451. We did not find any significant Φ_ST_ values in pairwise comparisons between stations 13–49. For nuclear 28S, pairwise Φ_ST_ values ranged from −0.001 to 0.621 for comparisons along the Atlantic transect, and from 0.079 to 0.532 for comparisons between Atlantic and Pacific samples (Table 3). The 28S pairwise Φ_ST_ values show a similar genetic break as for COI, separating stations 13–49 (North & Equatorial) from 51–64 (South), as 35 of the 40 pairwise Φ_ST_ values were significant (Φ_ST_ ranging from 0.012 to 0.621). There is additional spatial structuring separating samples from stations 13–29 (North) and 43–49 (Equatorial), as 13 of 17 pairwise Φ_ST_ comparisons were significant (Φ_ST_ ranging from −0.001 to 0.497, Table 3).

**TABLE 3:** Pairwise Φ_ST_ for mitochondrial cytochrome *c* oxidase I (COI) (below diagonal) and nuclear 28S (28S) genes (above the diagonal) between all samples of *Limacina bulimoides*

Station	North					Equatorial			South					Pacific
	13	19	23	25	29	43	45	49	51	53	55	60	62, 64	
13		**0.061**	0.039	0.034	**0.153**	**0.329**	**0.497**	**0.328**	0.025	**0.054**	**0.039**	**0.026**	0.037	**0.157**
19	0.010		0.005	0.039	0.020	**0.150**	**0.321**	**0.153**	**0.093**	**0.160**	**0.162**	**0.077**	**0.134**	**0.167**
23	0.000	−0.001		0.042	0.032	**0.163**	**0.329**	**0.168**	**0.070**	**0.120**	**0.134**	**0.062**	**0.101**	**0.154**
25	0.014	0.000	−0.004		**0.114**	**0.268**	**0.447**	**0.270**	0.042	**0.092**	**0.081**	0.035	0.062	**0.079**
29	0.012	0.014	0.003	−0.002		0.057	**0.205**	0.056	**0.190**	**0.273**	**0.304**	**0.137**	**0.241**	**0.244**
43	0.041	0.010	0.019	0.028	0.056		0.045	−0.001	**0.360**	**0.443**	**0.488**	**0.279**	**0.388**	**0.374**
45	0.002	0.000	0.006	0.003	0.005	0.034		0.052	**0.534**	**0.593**	**0.621**	**0.448**	**0.567**	**0.532**
49	0.019	0.011	0.012	0.006	0.022	0.032	0.012		**0.354**	**0.427**	**0.463**	**0.286**	**0.396**	**0.376**
51	**0.438**	**0.414**	**0.431**	**0.398**	**0.424**	**0.451**	**0.419**	**0.299**		0.023	0.013	0.012	0.065	**0.181**
53	**0.380**	**0.355**	**0.370**	**0.341**	**0.364**	**0.392**	**0.364**	**0.250**	0.007		0.036	0.023	0.070	**0.216**
55	**0.373**	**0.351**	**0.359**	**0.331**	**0.353**	**0.378**	**0.360**	**0.254**	0.005	−0.017		**0.028**	0.058	**0.242**
60	**0.408**	**0.389**	**0.400**	**0.369**	**0.391**	**0.416**	**0.398**	**0.289**	0.009	0.011	0.004		0.023	**0.106**
62, 64	**0.389**	**0.366**	**0.382**	**0.345**	**0.371**	**0.401**	**0.379**	**0.253**	0.022	−0.011	−0.009	0.021		**0.136**
Pacific	**0.780**	**0.769**	**0.781**	**0.773**	**0.778**	**0.783**	**0.772**	**0.742**	**0.759**	**0.748**	**0.731**	**0.724**	**0.735**	

Not<del author="Le Qin Choo" command="Delete" timestamp="1605599468055" title="Deleted by Le Qin Choo on 11/17/2020, 8:51:08 AM" class="reU3">e</del>

Significant Φ_ST_ values after Bonferroni correction (*α* = 0.05, *p* < 0.000549) are in bold.

**FIGURE 3:**
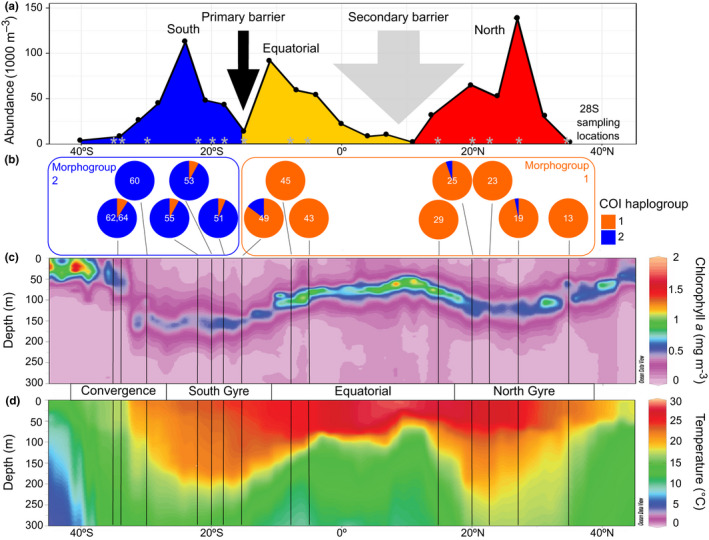
Environmental profile of two oceanic dispersal barriers for *Limacina bulimoides* in the Atlantic Ocean. The primary dispersal barrier is located at 15–18°S, while the secondary barrier is located in the region between 15°N and 4°S. (a) Abundance of *L. bulimoides* per 1,000 m^3^ seawater along the meridional transect sampled in 2014 (data from Burridge, Hörnlein, et al., 2017). Population groups are coloured as in Figure 2, supported by 28S genetic structuring (see Table 3) along the Atlantic Meridional Transect 22 (AMT22) sampled in 2012, locations indicated by grey asterisks. (b) Piecharts showing mitochondrial cytochrome *c* oxidase I (COI) haplogroup frequencies per sampling site with the division into two morphogroups superimposed (see also Figure 4b). (c) Ocean section plots of chlorophyll *a* concentration and (d) seawater temperature from the upper 300 metres of the water column with latitude. Sampling locations are marked by vertical lines, and major oceanographic provinces (Convergence, South Gyre, Equatorial, and North Gyre) are indicated. Plots b, c and d derive from material sampled in 2012 (AMT22 transect)

Hierarchical analyses of molecular variance show that the spatial structure based on the trimodal pattern of abundance of *L. bulimoides* across a similar Atlantic transect (AMT24, Figure 3a) explains the distribution of genetic variation better than partitioning based on oceanographic parameters (Table 4). For both COI and 28S fragments, the fixation index among groups was higher when partitioned among three abundance‐based 'population groups' (COI: Φ_CT_ = 0.285, *p* = 0.00129; 28S: Φ_CT_ = 0.280, *p* = 0.00010) than among four 'ocean provinces' (COI: Φ_CT_ = 0.205, *p* = 0.00446; 28S: Φ_CT_ = 0.140, *p* = 0.0620). Thus, we find a strong primary dispersal barrier separating stations 13–49 (North & Equatorial) from stations 51–64 (South) located at 15–18°S that is supported by both genes (Figure 3a,b). Surprisingly, this barrier is located in the core of the southern oligotrophic gyre and appears incongruent with any strong oceanographic gradients (Figure 3c,d). A more subtle secondary barrier is located in the region 15°N–4°S, which corresponds to the equatorial upwelling province and is mainly supported by 28S data.

**TABLE 4:** Results of hierarchical AMOVA for mitochondrial cytochrome *c* oxidase I (COI) (*n* = 334) and nuclear 28S (28S) (*n* = 324, 2*n* = 648) sequence variants in Atlantic individuals of *Limacina bulimoides*, partitioned based on four ocean provinces or three abundance‐based population groups (Table 1)

Gene	Grouping	*df*	Variance components	Percentage of variation (%)	Fixation indices
COI	Ocean provinces	3	Among groups	20.49	Φ_CT_ = 0.205**
		9	Among populations within groups	5.91	Φ_SC_ = 0.0743***
		321	Within populations	73.6	Φ_ST_ = 0.264***
	Population groups	2	Among groups	28.51	Φ_CT_ = 0.285**
		10	Among populations within groups	0.61	Φ_SC_ = 0.00849*
		321	Within populations	70.88	Φ_ST_ = 0.291***
28S	Ocean provinces	3	Among groups	14.02	Φ_CT_ = 0.140(*n*.s.)
		9	Among populations within groups	13.3	Φ_SC_ = 0.155***
		1,283	Within populations	72.67	Φ_ST_ = 0.273***
	Population groups	2	Among groups	27.98	Φ_CT_ = 0.280***
		10	Among populations within groups	2.88	Φ_SC_ = 0.0400***
		1,283	Within populations	69.13	Φ_ST_ = 0.309***

Note

Significance levels for fixation indices are indicated as follows: n.s.: not significant, *: *p* < 0.05, **: *p* < 0.01, ***: *p* < 0.001.

We did not find obvious patterns of IBD across the Atlantic transect (Figure S3). Although there was a significant correlation between the pairwise Φ_ST_ and geographic distance for COI along the complete Atlantic transect (*r* = 0.520, *p* = 0.00120, Table S2), this relationship breaks down when population groups on either side of the primary barrier at 15–18°S were analysed separately (North + Equatorial: *r* = 0.117, *p* = 0.269; South: *r* = 0.0968, *p* = 0.418, Table S2). Pairwise Φ_ST_ values were uniformly high regardless of geographic distance when comparing stations on opposite sides of this dispersal barrier, and uniformly low when comparing stations within population groups (Figure S3a). This suggests that the significant correlation along the entire transect can be explained by the inclusion of high pairwise Φ_ST_ values between the stations across the barrier. For 28S, correlation of genetic and geographic distance matrices was not observed for the entire Atlantic transect (*r* = −0.0942, *p* = 0.770), but only for the North + Equatorial group (*r* = 0.759, *p* = 0.003, Table S2; Figure S3b). Again, this correlation is likely driven by the high Φ_ST_ values for comparisons between North and Equatorial samples on either side of the secondary dispersal barrier. No IBD patterns were found when any of the population groups were analysed separately.

The two mitochondrial haplogroups appear diagnostic for individuals on either side of the primary dispersal barrier with most individuals from the North and Equatorial stations belonging to COI haplogroup 1 and most individuals from the South belonging to COI haplogroup 2 (Figure 3b). However, we found 13 exceptions that are probably expatriates, or individuals sampled outside of their core range. Seven expatriates from the South clustered within haplogroup 1, and six expatriates from the North + Equatorial groups clustered within haplogroup 2 (Table S3; Figure 3b). We could verify the life stage for nine of these expatriates, of which three were adults (shell length > 0.9 mm) and six were juveniles (Table S3). We assessed whether the 28S and mitochondrial haplotypes were congruent for 12 of these expatriates, but these results were inconclusive due to a lack of variability in the 28S fragment (Figure S4a).

### 3.3 Phenotypic variability

**FIGURE 4:**
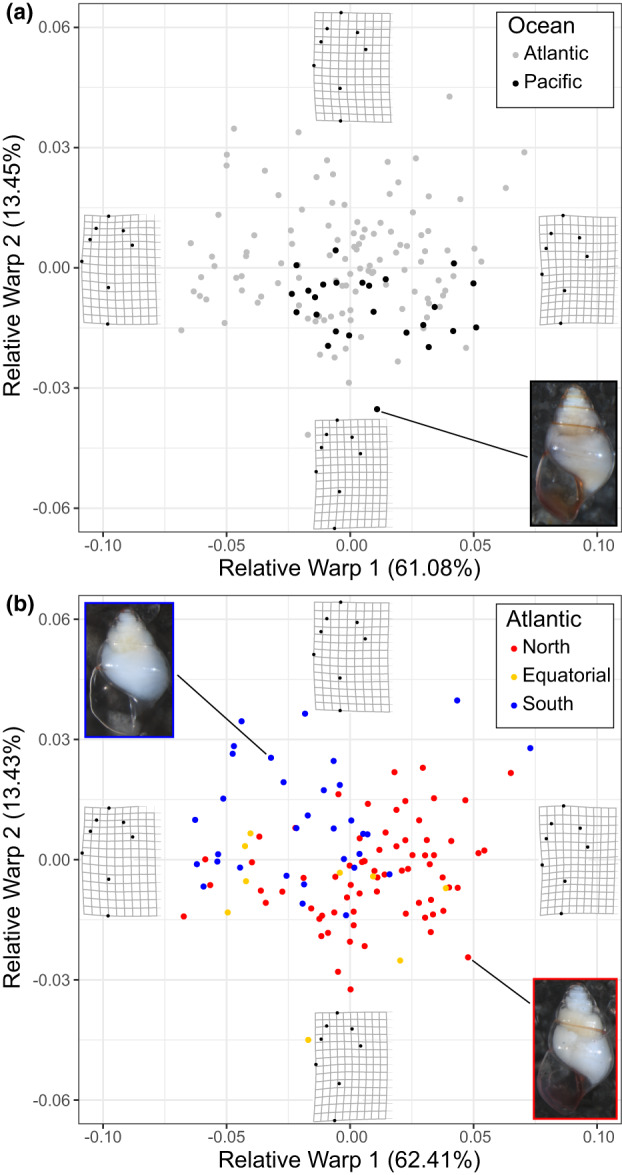
Shell shape variation in adult *Limacina bulimoides* visualized for the first two Relative Warp axes for (a) Atlantic and Pacific individuals (*N* = 136), and (b) Atlantic individuals (*N* = 111) coloured according to abundance‐based population groups. Thin‐plate splines of the most positive and negative deformations along the axes are indicated to depict the variation in shell shape. Photographs of typical individuals from the Atlantic and Pacific (a) and Atlantic North and South population groups (b) are also shown

The centroid size and RW axes 1, 2, 4, 6, and 7 were repeatable and included in the final analyses. Based on the complete dataset of adult *L. bulimoides*, including Pacific individuals (*N* = 136), the repeatable RWs accounted for 85.21% of the total shell shape variation (Table S4). Shell shape variation for all specimens (*N* = 136) and Atlantic specimens only (*N* = 111) was visualized on the first two axes, which explain the most variation: RW1 (All: 61.08%; Atlantic: 62.41%) and RW2 (All: 13.45%; Atlantic: 13.43%) (Figure 4). The thin‐plate spline reconstructions show that a positive RW1 is associated with a taller spire, while a negative RW1 is associated with a shorter, more rounded spire. Along RW2, larger values indicate wider shells.

We did not observe a significant difference in shell shape between Atlantic and Pacific *L. bulimoides* individuals (*N* = 136, *F* (1,135) = 1.66, *p* = 0.204). Shell shapes of the Pacific individuals (*N* = 25) fall within the variation observed for the Atlantic individuals (*N* = 111, Figure 4a). When Atlantic individuals were classified according to their spatial structure with North, Equatorial and South population groups, we found a significant difference in shell shape between groups based on the one‐way PERMANOVA (*F* (2,110) = 14.53, *p* = 0.0001). Shell shape was different between the North and South (*F* = 35.9, *p* = 0.003), whereas comparisons of shell shape between the North and Equatorial or South and Equatorial groups were not significant (*F* = 2.74, *p* = 0.113 and *F* = 2.67, *p* = 0.116, respectively). This result could be due to the low sample size of the Equatorial group (*N* = 9). Individuals from the North population group generally had a higher, narrower spire compared to individuals from the South (Figure 4b). Similarly, there is a significant difference in shell shape across all three groups (*F*(2, 108) = 29.79, *p* = 0.00000) based on the CVA. Significant shell shape differences were recorded for both North‐South (*p* = 0.00000) and Equatorial‐South (*p* = 0.00003) comparisons, but not between North and Equatorial groups (*p* = 0.889) based on Tukey HSD multiple pairwise comparisons (Figure S5, Table S5). Thus, the spatial structuring of samples in terms of shell shape matches the partitioning based on mitochondrial genetic variation (Figure 3b).

## 4 DISCUSSION

We found high levels of genetic variability and significant spatial population structure in the pteropod *L. bulimoides* along a latitudinal transect (35°N–36°S) in the Atlantic Ocean. Specifically, we have evidence for a primary dispersal barrier located in the southern subtropical gyre at 15–18°S and a more subtle, secondary barrier located in the region 15°N–4°S. This finding is in stark contrast to the historical perception of the pelagic environment as a homogeneous habitat lacking strong isolating barriers, and of holoplanktonic organisms as characterized by high dispersal and broad biogeographic ranges (Norris, 2000; Pierrot‐Bults & van der Spoel, 1979). Because shelled pteropods are regarded as sentinel species to assess the impacts of ocean acidification (Bednaršek, Klinger, et al., 2017), it is important to document their genetic variation and population structure, as these characteristics will influence their response to natural selection and global change (Barrett & Schluter, 2008; Donelson et al., 2019; Riebesell & Gattuso, 2015; Sanford & Kelly, 2011). This is the first study examining genetic structuring of a shelled pteropod with rigorous sampling at an ocean basin scale. The presence of strong oceanic dispersal barriers may indicate that the potential for range shifts and adaptive responses to a changing ocean could be more limited than expected, and that local adaptation should also be taken into account, for instance when modelling (future) species distribution patterns. Here, we discuss the nature and possible drivers of the oceanic dispersal barriers reported here for *L. bulimoides*, and more generally for pelagic taxa.

The primary dispersal barrier in the Atlantic Ocean probably represents a species barrier, across which populations on either side are reproductively isolated. This inference is supported by congruent differentiation at independently inherited genetic loci (COI and 28S) and congruent genetic and shell shape differences. Despite the significant IBD test for the COI gene across the Atlantic transect, the disappearance of this result when populations across the primary barrier were tested separately supports the inference that these populations are spatially discrete evolutionary lineages (Teske et al., 2018). Nevertheless, a direct test of reproductive isolation is not possible as the high mortality of pteropods under laboratory conditions precludes meaningful crossing experiments (Howes et al., 2014). Genome‐wide data would be necessary to determine the strength of reproductive isolation by quantifying divergence at many loci across the genome (Butlin & Stankowski, 2020; Gagnaire, 2020).

The observed morphometric variation was associated with the primary genetic barrier and not with any strong environmental transitions. Hence, it is more likely that the shell shape differences are the result of accumulated genetic differences than phenotypic plasticity resulting from distinct environments. Examples of strong plasticity in shells of marine molluscs are generally found across heterogeneous habitats, such as *L. saxatilis* with discrete ‘crab’ and ‘wave’ ecotypes (e.g. Hollander & Butlin, 2010; Hollander et al., 2006) or *Nacella concinna* with continuous morphological differentiation across a depth gradient (Hoffman et al., 2010). Shell shape variation has also been shown to have a large genetic component, with heritabilities ranging between 0.36 and 0.71 in *L. saxatilis* (Conde‐Padín et al., 2007) and between 0.16 and 0.56 in *Nucella lapillus* (Guerra‐Varela et al., 2009). Furthermore, shell shape differences in conjunction with genetic differentiation have been used to identify species boundaries in different genera of holoplanktonic gastropods (Burridge et al., 2015, 2019; Wall‐Palmer et al., 2018) using an integrative species concept (McManus & Katz, 2009; Padial et al., 2010). Hence, strong reproductive isolation is probably involved in the maintenance of this primary dispersal barrier in the southern Atlantic gyre.

The secondary dispersal barrier across the equatorial upwelling province is supported by limited evidence from the 28S gene (Table 3), and is congruent with known barriers in other pelagic taxa. The equatorial province has been suggested as an ecological barrier in some nekton species, such as Atlantic bluefin tuna (Briscoe et al., 2017) and whales (Holt et al., 2020), and as a barrier separating anti‐tropical clades in planktonic foraminifers (Casteleyn et al., 2010). Dispersal barriers across the equatorial upwelling region have also been observed for other zooplankton. For example, in the pteropod genus *Cuvierina*, two distinct morphotypes are found in the Atlantic basin. One morphotype, 'atlantica', has disjunct populations in the northern and southern subtropical gyres, which are genetically differentiated, while the morphotype 'cancapae' dominates in equatorial waters (Burridge et al., 2015). Similar patterns of equatorial dispersal barriers were observed in the epipelagic gastropods *Protatlanta sculpta* and *P. souleyeti* (Wall‐Palmer et al., 2016) and the mesopelagic copepod *H. longicornis* (Andrews et al., 2014; Norton & Goetze, 2013). We additionally observed a pattern of unique diversity in the equatorial region for the 28S gene fragment. This trend of higher equatorial diversity or equatorial endemics was also observed in populations of the copepods *P. abdominalis* (Hirai et al., 2015) and *P. xiphias* (Goetze et al., 2017). It was suggested that this unique diversity resulted from a resident equatorial population, which was supplemented with expatriates by advection from the neighbouring gyre populations (Goetze et al., 2017). It is as yet unknown if expatriates in *L. bulimoides* survive to reproduce with the resident population and contribute to gene flow, or whether expatriates represent evolutionary dead‐ends due to a mismatch of phenotype and environment (Marshall et al., 2010). Increased sampling intensity in the equatorial upwelling region, by having plankton tows with a greater amount of seawater filtered, would allow for the collection of more individuals in this region of low abundance for *L. bulimoides,* and a more thorough investigation into the prevalence and genetic identity of expatriates.

We identified two dispersal barriers for *L. bulimoides* in the Atlantic Ocean, of which one (the secondary barrier) appears more permeable than the other (the primary barrier). The locations of these barriers coincide with regions of low abundance, congruent with the hypothesis that areas of less suitable habitat contribute to the maintenance of population structure in oceanic plankton (Goetze et al., 2017; Norris, 2000; Peijnenburg et al., 2004). The presence of expatriates suggests that Atlantic *L. bulimoides* populations could meet and potentially interbreed. This raises the question: what are the possible isolating mechanisms? These may include overlooked physical boundaries or ecological differences, including their depth habitat, feeding strategy or reproductive biology. Interestingly, the primary dispersal barrier is congruent with complex mesopelagic circulation patterns in the South Atlantic (~20°S, Sutton et al., 2017) where a shift in shrimp species (*Acanthephyra kingsleyi* to *Acanthephyra quadrispinosa*) was observed at 18°S (Judkins, 2014). Little is known, however, about the behaviour or depth habitat of *L. bulimoides* across the Atlantic transect and thus it is possible that mesopelagic currents are important. Since internal fertilization is the reproductive mode in *L. bulimoides*, assortative mate choice could also be a possible reproductive isolating mechanism, with chemical cues facilitating mate location and communication, as observed in a variety of marine gastropods (Ng et al., 2013). Other isolating mechanisms could involve differences in reproductive timing (McClary & Barker, 1998), ecological selection and community interactions (Whittaker & Rynearson, 2017), or post‐zygotic barriers, such as reduced hybrid fitness (Lessios, 2007) or reduced hatching success of offspring (Ellingson & Krug, 2015). A broader range of ecological observations are required to determine the exact nature of dispersal barriers for *L. bulimoides*. For instance, future research to reveal population differences in feeding ecology or depth habitat could include the metabarcoding of gut contents, examination of the radula, or stable isotope analyses. Because we observe differences in the locations of maximum abundance and genetic transitions in different planktonic taxa sampled across similar Atlantic transects (Andrews et al., 2014; Goetze et al., 2015, 2017; Hirai et al., 2015; Norton & Goetze, 2013), we expect that dispersal barriers are species‐specific and largely driven by the ecological characteristics of the respective species.

Many circumglobally distributed marine species have distinct populations or (sub)species in different ocean basins. These subdivisions generally result from divergence in allopatry, that is separated by current systems or continental land masses, and are often congruent with global biogeochemical provinces (Bowen et al., 2016; Costello et al., 2017; Reygondeau et al., 2012). The significant genetic divergence between Atlantic and Pacific *L. bulimoides* is in agreement with the patterns of divergence observed between ocean basins in other circumglobally distributed marine taxa, including fish (e.g. Graves & McDowell, 2015), benthic organisms (e.g. Fauvelot et al., 2020; Prazeres et al., 2020) and other zooplankton (reviewed in Peijnenburg & Goetze, 2013). Based on a strict COI molecular clock rate of 2.4%/million years (MY) for gastropods (Hellberg & Vacquier, 1999), the divergence between the Atlantic and Pacific mitochondrial clades is estimated at ~4.6 MY, which roughly coincides with the emergence of the Isthmus of Panama (Bacon et al., 2015; O'Dea et al., 2016). The rise of the Isthmus of Panama has been directly linked to allopatric divergence across various marine taxa, due to the shoaling of the seaway, which reduced connectivity between mesopelagic populations at about 7 MY. This was followed by a decrease in seasonal upwelling due to the closing seaway at 4 MY, which cut off connectivity between plankton populations and decreased the input of primary productivity into the Caribbean (Knowlton et al., 1993; Leigh et al., 2014). This major marine biogeographic barrier was also used to calibrate the divergence between Atlantic and Pacific populations of three other pteropod species in a phylogenetic analysis (Burridge, Hörnlein, et al., 2017).

The population structure within the Atlantic basin could be the result of different, not mutually exclusive, evolutionary scenarios. Dispersal barriers could have resulted from secondary contact following allopatric divergence in the past. The divergence time of the two Atlantic haplogroups across the primary barrier is estimated at 1.10 million years (MY) using a strict COI molecular clock (Hellberg & Vacquier, 1999). This estimate points to the potential effect of Pleistocene glacial cycles on population divergence of *L. bulimoides*. The effect of Pleistocene cycles as important drivers of phylogeographic structure has also been proposed for many other marine molluscs (e.g. Luttikhuizen et al., 2003; Marko, 2004; Reid et al., 2006), including polar *Limacina* species (Sromek et al., 2015). Another possible scenario is parapatric divergence, where semi‐isolated populations living along an environmental cline diverged due to ecological selection. This would be a more plausible scenario for the secondary dispersal barrier across the equatorial upwelling gradients. The populations would progressively become more distinct, when the effect of divergent selection is greater than that of homogenizing gene flow (Crow et al., 2007; Luttikhuizen et al., 2003; Postel et al., 2020; Schluter, 2009). In order to differentiate between historical divergence followed by secondary contact, ecological selection or a combination of the two, genome‐wide analyses are required to investigate if genetic differentiation is randomly distributed across the genome (suggesting a gradual process) or associated with particular genes or environmental clines (indicative of ecological selection).

Holoplanktonic organisms are useful to gain insight into global biogeographical patterns in pelagic dispersal (Álvarez‐Noriega et al., 2020; Bradbury et al., 2008), as their dispersal potential is not confounded by settlement processes that involve complex biophysical interactions with coastal or benthic habitats (Pineda et al., 2009; Prairie et al., 2012; Weersing & Toonen, 2009). To gain further insight into the nature and drivers of dispersal barriers in the open ocean, as well as to identify signals of selection across the genome, a broader range of ecological observations and in‐depth analyses of genome‐wide diversity in zooplankton will be necessary (Bucklin et al., 2018; Choo et al., 2020; Choquet et al., 2019; Gagnaire et al., 2015). This is important because marine zooplankton are key players in pelagic food webs and useful indicators as rapid responders to environmental variation and climate change (Beaugrand et al., 2009). Shelled pteropods could be particularly suitable model organisms to gain insight into the evolutionary potential of marine zooplankton and to deepen our understanding of speciation processes in the open ocean for a number of reasons. First, there is growing interest in the group because of their vulnerability to ocean acidification and their potential use as bioindicators. Second, unlike most holoplanktonic animals, they have shells, which provide a record of the ecology and life history of an individual that can be easily measured. Third, pteropods are the only living planktonic animals with a good fossil record (Janssen & Peijnenburg, 2017; Peijnenburg et al., 2020), which allows for comparisons of morphological diversity in extant as well as extinct species and populations. We demonstrated significant spatial population structuring across and within ocean basins in *L. bulimoides*, a presumably circumglobal species. This underlying genetic variability and structure will influence the response of populations to global change, and in‐depth study of their life‐history traits, ecology and historical demography, in combination with experimental exposure to future conditions, will be required to more accurately predict their ability to adapt to a rapidly changing ocean.

## CONFLICT OF INTEREST

The authors have no conflict of interest to declare.

### Peer Review

The peer review history for this article is available at https://publons.com/publon/10.1111/jeb.13735

## Supporting information

Appendix S1

Video S1

## Data Availability

The nucleotide sequences for the COI and 28S genes are archived on NCBI GenBank with the accession numbers MN952611–MN952965 and MN950433–MN950775, respectively. Images for morphometric analyses are archived on Dryad (https://doi.org/10.5061/dryad.p8cz8w9nq).

## References

[jeb13735-bib-0001] Álvarez‐Noriega, M. , Burgess, S. C. , Byers, J. E. , Pringle, J. M. , Wares, J. P. , & Marshall, D. J. (2020). Global biogeography of marine dispersal potential. Nature Ecology & Evolution, 4(9), 1196–1203. 10.1038/s41559-020-1238-y 32632257

[jeb13735-bib-0002] Andrews, K. R. , Norton, E. L. , Fernandez‐Silva, I. , Portner, E. , & Goetze, E. (2014). Multilocus evidence for globally distributed cryptic species and distinct populations across ocean gyres in a mesopelagic copepod. Molecular Ecology, 23(22), 5462–5479. 10.1111/mec.12950 25283587

[jeb13735-bib-0003] Bacon, C. D. , Silvestro, D. , Jaramillo, C. , Smith, B. T. , Chakrabarty, P. , & Antonelli, A. (2015). Biological evidence supports an early and complex emergence of the Isthmus of Panama. Proceedings of the National Academy of Sciences of the United States of America, 112(19), 6110–6115. 10.1073/pnas.1423853112 25918375 PMC4434730

[jeb13735-bib-0004] Barrett, R. D. H. , & Schluter, D. (2008). Adaptation from standing genetic variation. Trends in Ecology and Evolution, 23(1), 38–44. 10.1016/j.tree.2007.09.008 18006185

[jeb13735-bib-0005] Barth, J. M. I. , Berg, P. R. , Jonsson, P. R. , Bonanomi, S. , Corell, H. , Hemmer‐Hansen, J. , Jakobsen, K. S. , Johannesson, K. , Jorde, P. E. , Knutsen, H. , Moksnes, P.‐O. , Star, B. , Stenseth, N. C. , Svedäng, H. , Jentoft, S. , & André, C. (2017). Genome architecture enables local adaptation of Atlantic cod despite high connectivity. Molecular Ecology, 26(17), 4452–4466. 10.1111/mec.14207 28626905

[jeb13735-bib-0006] Bé, A. W. H. , & Gilmer, R. (1977). A zoogeographic and taxonomic review of Euthecosomatous Pteropoda. Oceanic Micropaleontology, 1(6), 733–808.

[jeb13735-bib-0007] Beaugrand, G. , Goberville, E. , Luczak, C. , & Kirby, R. R. (2014). Marine biological shifts and climate. Proceedings of the Royal Society B: Biological Sciences, 281(1783), 20133350 10.1098/rspb.2013.3350 PMC3996605 24718760

[jeb13735-bib-0008] Beaugrand, G. , Luczak, C. , & Edwards, M. (2009). Rapid biogeographical plankton shifts in the North Atlantic Ocean. Global Change Biology, 15(7), 1790–1803. 10.1111/j.1365-2486.2009.01848.x

[jeb13735-bib-0009] Bednaršek, N. , Feely, R. A. , Tolimieri, N. , Hermann, A. J. , Siedlecki, S. A. , Waldbusser, G. G. , McElhany, P. , Alin, S. R. , Klinger, T. , Moore‐Maley, B. , & Pörtner, H. O. (2017). Exposure history determines pteropod vulnerability to ocean acidification along the US West Coast article. Scientific Reports, 7(1), 1–12. 10.1038/s41598-017-03934-z 28674406 PMC5495755

[jeb13735-bib-0010] Bednaršek, N. , Klinger, T. , Harvey, C. J. , Weisberg, S. , McCabe, R. M. , Feely, R. A. , Newton, J. , & Tolimieri, N. (2017). New ocean, new needs: Application of pteropod shell dissolution as a biological indicator for marine resource management. Ecological Indicators, 76, 240–244. 10.1016/j.ecolind.2017.01.025

[jeb13735-bib-0011] Bednaršek, N. , Možina, J. , Vogt, M. , O'Brien, C. , & Tarling, G. A. (2012). The global distribution of pteropods and their contribution to carbonate and carbon biomass in the modern ocean. Earth System Science Data, 4(1), 167–186. 10.5194/essd-4-167-2012

[jeb13735-bib-0012] Bednaršek, N. , Tarling, G. A. , Bakker, D. C. E. , Fielding, S. , Jones, E. M. , Venables, H. J. , Ward, P. , Kuzirian, A. , Lézé, B. , Feely, R. A. , & Murphy, E. J. (2012). Extensive dissolution of live pteropods in the Southern Ocean. Nature Geoscience, 5, 881–885. 10.1038/NGEO1635

[jeb13735-bib-0013] Bell, G. (2013). Evolutionary rescue and the limits of adaptation. Philosophical Transactions of the Royal Society B: Biological Sciences, 368(1610), 1–6. 10.1098/rstb.2012.0080 PMC3538447 23209162

[jeb13735-bib-0014] Bierne, N. , Bonhomme, F. , & David, P. (2003). Habitat preference and the marine‐speciation paradox. Proceedings of the Royal Society B: Biological Sciences, 270(1522), 1399–1406. 10.1098/rspb.2003.2404 PMC1691380 12965032

[jeb13735-bib-0015] Bogan, S. N. , Johnson, K. M. , & Hofmann, G. E. (2020). Changes in Genome‐Wide Methylation and Gene Expression in Response to Future pCO2 Extremes in the Antarctic Pteropod *Limacina helicina antarctica* . Frontiers in Marine Science, 6, 1–13. 10.3389/fmars.2019.00788

[jeb13735-bib-0016] Bowen, B. W. , Gaither, M. R. , DiBattista, J. D. , Iacchei, M. , Andrews, K. R. , Grant, W. S. , Toonen, R. J. , & Briggs, J. C. (2016). Comparative phylogeography of the ocean planet. Proceedings of the National Academy of Sciences of the United States of America, 113(29), 7962–7969. 10.1073/pnas.1602404113 27432963 PMC4961182

[jeb13735-bib-0017] Bradbury, I. R. , Laurel, B. , Snelgrove, P. V. R. , Bentzen, P. , & Campana, S. E. (2008). Global patterns in marine dispersal estimates: The influence of geography, taxonomic category and life history. Proceedings of the Royal Society B: Biological Sciences, 275(1644), 1803–1809. 10.1098/rspb.2008.0216 PMC2587791 18445556

[jeb13735-bib-0018] Briscoe, D. K. , Hobday, A. J. , Carlisle, A. , Scales, K. , Eveson, J. P. , Arrizabalaga, H. , Fromentin, J. M. (2017). Ecological bridges and barriers in pelagic ecosystems. Deep Sea Research Part II: Topical Studies in Oceanography, 140, 182–192. 10.1016/j.dsr2.2016.11.004

[jeb13735-bib-0019] Bucklin, A. , DiVito, K. , Smolina, I. , Choquet, M. , Questel, J. M. , Hoarau, G. , & O'Neill, R. J. (2018). Population genomics of marine zooplankton In M. Oleksiak , & O. P. Rajora (Eds.), Population genomics: Marine organisms (1st ed.). Springer International Publishing 10.1007/13836_2017_9

[jeb13735-bib-0020] Buitenhuis, E. T. , Le Quéré, C. , Bednaršek, N. , & Schiebel, R. (2019). Large contribution of pteropods to shallow CaCO3 export. Global Biogeochemical Cycles, 33(3), 458–468. 10.1029/2018GB006110

[jeb13735-bib-0021] Burridge, A. K. , Goetze, E. , Raes, N. , Huisman, J. , & Peijnenburg, K. T. C. A. (2015). Global biogeography and evolution of *Cuvierina* pteropods. BMC Evolutionary Biology, 15(1), 1–16. 10.1186/s12862-015-0310-8 25880735 PMC4443520

[jeb13735-bib-0022] Burridge, A. K. , Goetze, E. , Wall‐Palmer, D. , Le Double, S. L. , Huisman, J. , & Peijnenburg, K. T. C. A. (2017). Diversity and abundance of pteropods and heteropods along a latitudinal gradient across the Atlantic Ocean. Progress in Oceanography, 158, 213–223. 10.1016/j.pocean.2016.10.001

[jeb13735-bib-0023] Burridge, A. K. , Hörnlein, C. , Janssen, A. W. , Hughes, M. , Bush, S. L. , Marlétaz, F. , Gasca, R. , Pierrot‐Bults, A. C. , Michel, E. , Todd, J. A. , Young, J. R. , Osborn, K. J. , Menken, S. B. J. , & Peijnenburg, K. T. C. A. (2017). Time‐calibrated molecular phylogeny of pteropods. PLoS One, 12(6), 1–22. 10.1371/journal.pone.0177325 PMC5467808 28604805

[jeb13735-bib-0024] Burridge, A. K. , Tump, M. , Vonk, R. , Goetze, E. , & Peijnenburg, K. T. C. A. (2017). Diversity and distribution of hyperiid amphipods along a latitudinal transect in the Atlantic Ocean. Progress in Oceanography, 158, 224–235. 10.1016/j.pocean.2016.08.003

[jeb13735-bib-0025] Burridge, A. K. , Van der Hulst, R. , Goetze, E. , & Peijnenburg, K. T. C. A. (2019). Assessing species boundaries in the open sea: An integrative taxonomic approach to the pteropod genus Diacavolinia. Zoological Journal of the Linnean Society, 187(4), 1016–1040. 10.1093/zoolinnean/zlz049

[jeb13735-bib-0026] Butlin, R. K. , Saura, M. , Charrier, G. , Jackson, B. , André, C. , Caballero, A. , Coyne, J. A. , Galindo, J. , Grahame, J. W. , Hollander, J. , Kemppainen, P. , Martínez‐Fernández, M. , Panova, M. , Quesada, H. , Johannesson, K. , & Rolán‐Alvarez, E. (2014). Parallel evolution of local adaptation and reproductive isolation in the face of gene flow. Evolution, 68(4), 935–949. 10.1111/evo.12329 24299519 PMC4261988

[jeb13735-bib-0027] Butlin, R. K. , & Stankowski, S. (2020). Is it time to abandon the biological species concept? No. National Science Review, 7(8), 1400–1401. 10.1093/nsr/nwaa109 PMC8288981 34692168

[jeb13735-bib-0028] Casteleyn, G. , Leliaert, F. , Backeljau, T. , Debeer, A.‐E. , Kotaki, Y. , Rhodes, L. , Lundholm, N. , Sabbe, K. , & Vyverman, W. (2010). Limits to gene flow in a cosmopolitan marine planktonic diatom. Proceedings of the National Academy of Sciences of the United States of America, 107(29), 12952–12957. 10.1073/pnas.1001380107 20615950 PMC2919969

[jeb13735-bib-0029] Choo, L. Q. , Bal, T. M. P. , Choquet, M. , Smolina, I. , Ramos‐Silva, P. , Marlétaz, F. , Kopp, M. , Hoarau, G. , & Peijnenburg, K. T. C. A. (2020). Novel genomic resources for shelled pteropods: A draft genome and target capture probes for *Limacina bulimoides*, tested for cross‐species relevance. BMC Genomics, 21(1), 1–14. 10.1186/s12864-019-6372-z PMC6942316 31900119

[jeb13735-bib-0030] Choquet, M. , Smolina, I. , Dhanasiri, A. K. S. , Kopp, M. , Jueterbock, A. , Sundaram, A. Y. M. , & Hoarau, G. (2019). Towards population genomics in non‐model species with large genomes; a case study of the marine zooplankton *Calanus finmarchicus* . Royal Society Open Science, 6, 1–36. 10.1098/RSOS.180608 PMC6408391 30891252

[jeb13735-bib-0031] Conde‐Padín, P. , Carvajal‐Rodríguez, A. , Carballo, M. , Caballero, A. , & Rolán‐Alvarez, E. (2007). Genetic variation for shell traits in a direct‐developing marine snail involved in a putative sympatric ecological speciation process. Evolutionary Ecology, 21(5), 635–650. 10.1007/s10682-006-9142-8

[jeb13735-bib-0032] Costello, M. J. , Tsai, P. , Wong, P. S. , Cheung, A. K. L. , Basher, Z. , & Chaudhary, C. (2017). Marine biogeographic realms and species endemicity. Nature Communications, 8(1), 1–9. 10.1038/s41467-017-01121-2 PMC5648874 29051522

[jeb13735-bib-0033] Crow, K. D. , Munehara, H. , Kanamoto, Z. , Balanov, A. , Antonenko, D. , & Bernardi, G. (2007). Maintenance of species boundaries despite rampant hybridization between three species of reef fishes (Hexagrammidae): Implications for the role of selection. Biological Journal of the Linnean Society, 91(1), 135–147. 10.1111/j.1095-8312.2007.00786.x

[jeb13735-bib-0034] Dam, H. G. (2013). Evolutionary adaptation of marine zooplankton to global change. Annual Review of Marine Science, 5(1), 349–370. 10.1146/annurev-marine-121211-172229 22809192

[jeb13735-bib-0035] Dayrat, B. , Tillier, A. , Lecointre, G. , & Tillier, S. (2001). New clades of euthyneuran gastropods (Mollusca) from 28S rRNA sequences. Molecular Phylogenetics and Evolution, 19(2), 225–235. 10.1006/mpev.2001.0926 11341805

[jeb13735-bib-0036] De Vargas, C. , Norris, R. , Zaninetti, L. , Gibb, S. W. , & Pawlowski, J. (1999). Molecular evidence of cryptic speciation in planktonic foraminifers and their relation to oceanic provinces. Proceedings of the National Academy of Sciences of the United States of America, 96(6), 2864–2868. 10.1073/pnas.96.6.2864 10077602 PMC15860

[jeb13735-bib-0037] Donelson, J. M. , Sunday, J. M. , Figueira, W. F. , Gaitán‐Espitia, J. D. , Hobday, A. J. , Johnson, C. R. , Leis, J. M. , Ling, S. D. , Marshall, D. , Pandolfi, J. M. , Pecl, G. , Rodgers, G. G. , Booth, D. J. , & Munday, P. L. (2019). Understanding interactions between plasticity, adaptation and range shifts in response to marine environmental change. Philosophical Transactions of the Royal Society B: Biological Sciences, 374(1768), 20180186 10.1098/rstb.2018.0186 PMC6365866 30966966

[jeb13735-bib-0038] Doney, S. C. , Ruckelshaus, M. , Emmett Duffy, J. , Barry, J. P. , Chan, F. , English, C. A. , Galindo, H. M. , Grebmeier, J. M. , Hollowed, A. B. , Knowlton, N. , Polovina, J. , Rabalais, N. N. , Sydeman, W. J. , & Talley, L. D. (2012). Climate change impacts on marine ecosystems. Annual Review of Marine Science, 4(1), 11–37. 10.1146/annurev-marine-041911-111611 22457967

[jeb13735-bib-0039] Ellingson, R. A. , & Krug, P. J. (2015). Reduced genetic diversity and increased reproductive isolation follow population‐level loss of larval dispersal in a marine gastropod. Evolution, 70(1), 18–37. 10.1111/evo.12830 26635309

[jeb13735-bib-0040] Excoffier, L. , & Lischer, H. E. L. (2010). Arlequin suite ver 3.5: A new series of programs to perform population genetics analyses under Linux and Windows. Molecular Ecology Resources, 10(3), 564–567. 10.1111/j.1755-0998.2010.02847.x 21565059

[jeb13735-bib-0041] Fauvelot, C. , Zuccon, D. , Borsa, P. , Grulois, D. , Magalon, H. , Riquet, F. , Bouchet, P. (2020). Phylogeographical patterns and a cryptic species provide new insights into Western Indian Ocean giant clams phylogenetic relationships and colonization history. Journal of Biogeography, 47(5), 1086–1105. 10.1111/jbi.13797

[jeb13735-bib-0042] Gagnaire, P. A. (2020). Comparative genomics approach to evolutionary process connectivity. Evolutionary Applications, 13(6), 1320–1334. 10.1111/eva.12978 32684961 PMC7359831

[jeb13735-bib-0043] Gagnaire, P.‐A. , Broquet, T. , Aurelle, D. , Viard, F. , Souissi, A. , Bonhomme, F. , Arnaud‐Haond, S. , & Bierne, N. (2015). Using neutral, selected, and hitchhiker loci to assess connectivity of marine populations in the genomic era. Evolutionary Applications, 8(8), 769–786. 10.1111/eva.12288 26366195 PMC4561567

[jeb13735-bib-0044] Gattuso, J. P. , Magnan, A. , Billé, R. , Cheung, W. W. L. , Howes, E. L. , Joos, F. , Allemand, D. , Bopp, L. , Cooley, S. R. , Eakin, C. M. , Hoegh‐Guldberg, O. , Kelly, R. P. , Pörtner, H.‐O. , Rogers, A. D. , Baxter, J. M. , Laffoley, D. , Osborn, D. , Rankovic, A. , & Rochette, J. , … Turley, C. (2015). Contrasting futures for ocean and society from different anthropogenic CO2 emissions scenarios. Science, 349(6243), aac4722 10.1126/science.aac4722 26138982

[jeb13735-bib-0045] Geller, J. , Meyer, C. , Parker, M. , & Hawk, H. (2013). Redesign of PCR primers for mitochondrial cytochrome c oxidase subunit I for marine invertebrates and application in all‐taxa biotic surveys. Molecular Ecology Resources, 13(5), 851–861. 10.1111/1755-0998.12138 23848937

[jeb13735-bib-0046] Gilmer, R. W. , & Harbison, G. R. (1986). Morphology and field behavior of pteropod molluscs: Feeding methods in the families Cavoliniidae, Limacinidae and Peraclididae (Gastropoda: Thecosomata). Marine Biology, 91(1), 47–57. 10.1007/BF00397570

[jeb13735-bib-0047] Gleason, L. U. , & Burton, R. S. (2016). Genomic evidence for ecological divergence against a background of population homogeneity in the marine snail *Chlorostoma funebralis* . Molecular Ecology, 25(15), 3557–3573. 10.1111/mec.13703 27199218

[jeb13735-bib-0048] Goetze, E. , Andrews, K. R. , Peijnenburg, K. T. C. A. , Portner, E. , Norton, E. L. , & Dam, H. G. (2015). Temporal stability of genetic structure in a mesopelagic copepod. PLoS One, 10(8), 1–16. 10.1371/journal.pone.0136087 PMC4547763 26302332

[jeb13735-bib-0049] Goetze, E. , Hüdepohl, P. T. , Chang, C. , Van Woudenberg, L. , Iacchei, M. , & Peijnenburg, K. T. C. A. (2017). Ecological dispersal barrier across the equatorial Atlantic in a migratory planktonic copepod. Progress in Oceanography, 158, 203–212. 10.1016/j.pocean.2016.07.001

[jeb13735-bib-0140] Graves, J. E. , & McDowell, J. R. (2015). Population structure of istiophorid billfishes. Fisheries Research, 166, 21–28. 10.1016/j.fishres.2014.08.016

[jeb13735-bib-0050] Gruber, N. , Clement, D. , Carter, B. R. , Feely, R. A. , van Heuven, S. , Hoppema, M. , & Wanninkhof, R. (2019). The oceanic sink for anthropogenic CO_2_ from 1994 to 2007. Science, 363(6432), 1193–1199. 10.1126/science.aau5153 30872519

[jeb13735-bib-0051] Guerra‐Varela, J. , Colson, I. , Backeljau, T. , Breugelmans, K. , Hughes, R. N. , & Rolán‐Alvarez, E. (2009). The evolutionary mechanism maintaining shell shape and molecular differentiation between two ecotypes of the dogwhelk *Nucella lapillus* . Evolutionary Ecology, 23(2), 261–280. 10.1007/s10682-007-9221-5

[jeb13735-bib-0052] Hammer, Ø. , Harper, D. A. T. , & Ryan, P. D. (2001). PAST: Paleontological statistics software package for education and data analysis. Paleontologica Electronica, 4(1), 9

[jeb13735-bib-0053] Harvey, B. , Al‐Janabi, B. , Broszeit, S. , Cioffi, R. , Kumar, A. , Aranguren‐Gassis, M. , Bailey, A. , Green, L. , Gsottbauer, C. , Hall, E. , Lechler, M. , Mancuso, F. , Pereira, C. , Ricevuto, E. , Schram, J. , Stapp, L. , Stenberg, S. , & Rosa, L. (2014). Evolution of marine organisms under climate change at different levels of biological organisation. Water (Switzerland), 6(11), 3545–3574. 10.3390/w6113545

[jeb13735-bib-0054] Hays, G. C. , Richardson, A. J. , & Robinson, C. (2005). Climate change and marine plankton. Trends in Ecology and Evolution, 20(6), 337–344. 10.1016/j.tree.2005.03.004 16701390

[jeb13735-bib-0055] Hellberg, M. E. , & Vacquier, V. D. (1999). Rapid evolution of fertilization selectivity and lysin cDNA sequences in Teguline gastropods. Molecular Biology and Evolution, 16(6), 839–848. 10.1093/oxfordjournals.molbev.a026168 10368961

[jeb13735-bib-0056] Hirai, J. , Tsuda, A. , & Goetze, E. (2015). Extensive genetic diversity and endemism across the global range of the oceanic copepod *Pleuromamma abdominalis* . Progress in Oceanography, 138, 77–90. 10.1016/j.pocean.2015.09.002

[jeb13735-bib-0057] Hoffman, J. I. , Peck, L. S. , Hillyard, G. , Zieritz, A. , & Clark, M. S. (2010). No evidence for genetic differentiation between Antarctic limpet *Nacella concinna* morphotypes. Marine Biology, 157(4), 765–778. 10.1007/s00227-009-1360-5

[jeb13735-bib-0058] Hollander, J. , & Butlin, R. K. (2010). The adaptive value of phenotypic plasticity in two ecotypes of a marine gastropod. BMC Evolutionary Biology, 10(1), 333 10.1186/1471-2148-10-333 21029403 PMC2984422

[jeb13735-bib-0059] Hollander, J. , Collyer, M. L. , Adams, D. C. , & Johannesson, K. (2006). Phenotypic plasticity in two marine snails: Constraints superseding life history. Journal of Evolutionary Biology, 19(6), 1861–1872. 10.1111/j.1420-9101.2006.01171.x 17040383

[jeb13735-bib-0060] Holsinger, K. E. , & Weir, B. S. (2009). Genetics in geographically structured populations: Defining, estimating and interpreting FST. Nature Reviews Genetics, 10(9), 639–650. 10.1038/nrg2611 PMC4687486 19687804

[jeb13735-bib-0061] Holt, B. G. , Marx, F. G. , Fritz, S. A. , Lessard, J.‐P. , & Rahbek, C. (2020). Evolutionary diversification in the marine realm: A global case study with marine mammals. Frontiers of Biogeography, 12(3), 1–19. 10.21425/F5FBG45184

[jeb13735-bib-0062] Howes, E. L. , Bednaršek, N. , Büdenbender, J. , Comeau, S. , Doubleday, A. , Gallager, S. M. , Hopcroft, R. R. , Lischka, S. , Maas, A. E. , Bijma, J. , & Gattuso, J.‐P. (2014). Sink and swim: A status review of thecosome pteropod culture techniques. Journal of Plankton Research, 36(2), 299–315. 10.1093/plankt/fbu002

[jeb13735-bib-0063] Hunt, B. P. V. , Pakhomov, E. A. , Hosie, G. W. , Siegel, V. , Ward, P. , & Bernard, K. (2008). Pteropods in Southern Ocean ecosystems. Progress in Oceanography, 78(3), 193–221. 10.1016/j.pocean.2008.06.001

[jeb13735-bib-0064] Janssen, A. W. , & Peijnenburg, K. T. C. (2017). An overview of the fossil record of Pteropoda (Mollusca, Gastropoda, Heterobranchia). Cainozoic Research, 1, 3–10.

[jeb13735-bib-0065] Jennings, R. M. , Bucklin, A. , Ossenbrügger, H. , & Hopcroft, R. R. (2010). Species diversity of planktonic gastropods (Pteropoda and Heteropoda) from six ocean regions based on DNA barcode analysis. Deep Sea Research Part II: Topical Studies in Oceanography, 57(24–26), 2199–2210. 10.1016/j.dsr2.2010.09.022

[jeb13735-bib-0066] Johannesson, K. , Butlin, R. K. , Panova, M. , & Westram, A. M. (2017). Mechanisms of Adaptive Divergence and Speciation in *Littorina saxatilis*: Integrating Knowledge from Ecology and Genetics with New Data Emerging from Genomic Studies In M. F. Oleksiak & O. P. Rajora (Eds.), Population genomics: marine organisms (pp. 277–301). Cham, Switzerland: Springer 10.1007/13836_2017_6

[jeb13735-bib-0067] Judkins, D. C. (2014). Geographical distribution of pelagic decapod shrimp in the Atlantic Ocean. Zootaxa, 3895(3), 301–345. 10.11646/zootaxa.3895.3.1 25543573

[jeb13735-bib-0068] Katoh, K. , & Standley, D. M. (2013). MAFFT multiple sequence alignment software version 7: Improvements in performance and usability. Molecular Biology and Evolution, 30(4), 772–780. 10.1093/molbev/mst010 23329690 PMC3603318

[jeb13735-bib-0069] Kearse, M. , Moir, R. , Wilson, A. , Stones‐Havas, S. , Cheung, M. , Sturrock, S. , Buxton, S. , Cooper, A. , Markowitz, S. , Duran, C. , Thierer, T. , Ashton, B. , Meintjes, P. , & Drummond, A. (2012). Geneious Basic: An integrated and extendable desktop software platform for the organization and analysis of sequence data. Bioinformatics, 28(12), 1647–1649. 10.1093/bioinformatics/bts199 22543367 PMC3371832

[jeb13735-bib-0070] Knowlton, N. , Weight, L. A. , Solórzano, L. A. , Mills, D. K. , & Bermingham, E. (1993). Divergence in proteins, mitochondrial DNA, and reproductive compatibility across the isthmus of Panama. Science, 260(5114), 1629–1632. 8503007 10.1126/science.8503007

[jeb13735-bib-0071] Kroeker, K. J. , Kordas, R. L. , Crim, R. , Hendriks, I. E. , Ramajo, L. , Singh, G. S. , Duarte, C. M. , & Gattuso, J.‐P. (2013). Impacts of ocean acidification on marine organisms: Quantifying sensitivities and interaction with warming. Global Change Biology, 19(6), 1884–1896. 10.1111/gcb.12179 23505245 PMC3664023

[jeb13735-bib-0072] Laibl, C. F. , Schrödl, M. , & Kohnert, P. C. (2019). 3D‐microanatomy of a keystone planktonic species, the northern polar pteropod *Limacina helicina helicina* (Gastropoda: Heterobranchia). Journal of Molluscan Studies, 85(1), 133–142. 10.1093/mollus/eyy063

[jeb13735-bib-0073] Lalli, C. M. , & Gilmer, R. W. (1989). Pelagic snails: The biology of holoplanktonic gastropod molluscs. Stanford University Press.

[jeb13735-bib-0074] Lalli, C. M. , & Wells, F. E. (1978). Reproduction in the genus *Limacina* (Ophistobranchia: Thecosomata). Journal of Zoology, 186, 95–108.

[jeb13735-bib-0075] Leigh, E. G. , O'Dea, A. , & Vermeij, G. J. (2014). Historical biogeography of the Isthmus of Panama. Biological Reviews, 89(1), 148–172. 10.1111/brv.12048 23869709

[jeb13735-bib-0076] Leigh, J. W. , & Bryant, D. (2015). POPART: Full‐feature software for haplotype network construction. Methods in Ecology and Evolution, 6(9), 1110–1116. 10.1111/2041-210X.12410

[jeb13735-bib-0077] Lessios, H. A. (2007). Reproductive isolation between species of sea urchins. Bulletin of Marine Science, 81(2), 191–208.

[jeb13735-bib-0078] Lischka, S. , Büdenbender, J. , Boxhammer, T. , & Riebesell, U. (2011). Impact of ocean acidification and elevated temperatures on early juveniles of the polar shelled pteropod *Limacina helicina*: Mortality, shell degradation, and shell growth. Biogeosciences, 8, 919–932. 10.5194/bg-8-919-2011

[jeb13735-bib-0079] Longhurst, A. R. (2007). Ecological geography of the Sea. Academic Press 10.1016/B978-0-12-455521-1.X5000-1

[jeb13735-bib-0080] Luttikhuizen, P. C. , Drent, J. , & Baker, A. J. (2003). Disjunct distribution of highly diverged mitochondrial lineage clade and population subdivision in a marine bivalve with pelagic larval dispersal. Molecular Ecology, 12(8), 2215–2229. 10.1046/j.1365-294X.2003.01872.x 12859640

[jeb13735-bib-0081] Maas, A. E. , Lawson, G. L. , Bergan, A. J. , & Tarrant, A. M. (2018). Exposure to CO2 influences metabolism, calcification, and gene expression of the thecosome pteropod *Limacina retroversa* . The Journal of Experimental Biology, 221, 164400 10.1242/jeb.164400 29191863

[jeb13735-bib-0082] Manno, C. , Bednaršek, N. , Tarling, G. A. , Peck, V. L. , Comeau, S. , Adhikari, D. , Bakker, D. C. E. , Bauerfeind, E. , Bergan, A. J. , Berning, M. I. , Buitenhuis, E. , Burridge, A. K. , Chierici, M. , Flöter, S. , Fransson, A. , Gardner, J. , Howes, E. L. , Keul, N. , Kimoto, K. , … Ziveri, P. (2017). Shelled pteropods in peril: Assessing vulnerability in a high CO2 ocean. Earth‐Science Reviews, 169, 132–145. 10.1016/j.earscirev.2017.04.005

[jeb13735-bib-0083] Marko, P. B. (2004). “What's larvae got to do with it?” Disparate patterns of post‐glacial population structure in two benthic marine gastropods with identical dispersal potential. Molecular Ecology, 13(3), 597–611. 10.1046/j.1365-294X.2004.02096.x 14871364

[jeb13735-bib-0084] Marshall, D. J. , Monro, K. , Bode, M. , Keough, M. J. , & Swearer, S. (2010). Phenotype‐environment mismatches reduce connectivity in the sea. Ecology Letters, 13(1), 128–140. 10.1111/j.1461-0248.2009.01408.x 19968695

[jeb13735-bib-0085] Marshall, D. J. , & Morgan, S. G. (2011). Ecological and evolutionary consequences of linked life‐history stages in the sea. Current Biology, 21(18), R718–R725. 10.1016/j.cub.2011.08.022 21959162

[jeb13735-bib-0086] McClary, D. , & Barker, M. (1998). Reproductive Isolation? Interannual variability in the timing of reproduction in sympatric sea urchins, Genus *Pseudechinus* . Invertebrate Biology, 117(1), 75–93.

[jeb13735-bib-0087] McManus, G. B. , & Katz, L. A. (2009). Molecular and morphological methods for identifying plankton: What makes a successful marriage? Journal of Plankton Research, 31(10), 1119–1129. 10.1093/plankt/fbp061

[jeb13735-bib-0088] Meisenheimer, J. (1905). Die Pteropoden der Deutsche Südpolar Expedition 1901–1903. Reimer.

[jeb13735-bib-0089] Miller, D. D. , Ota, Y. , Sumaila, U. R. , Cisneros‐Montemayor, A. M. , & Cheung, W. W. L. (2018). Adaptation strategies to climate change in marine systems. Global Change Biology, 24(1), e1–e14. 10.1111/gcb.13829 28727217

[jeb13735-bib-0090] Morton, J. E. (1954). The pelagic Mollusca of Benguela current Part I. First survey, R.R.S “William Scoresby”, March 1950 With an account of the reproductive system and sexual succession of *Limacina bulimoides* Discovery Reports (Vol. 27, pp. 163–200). Cambridge: University Press.

[jeb13735-bib-0091] Moya, A. , Howes, E. L. , Lacoue‐Labarthe, T. , Forêt, S. , Hanna, B. , Medina, M. , Munday, P. L. , Ong, J.‐S. , Teyssié, J.‐L. , Torda, G. , Watson, S.‐A. , Miller, D. J. , Bijma, J. , & Gattuso, J. P. (2016). Near‐future pH conditions severely impact calcification, metabolism and the nervous system in the pteropod *Heliconoides inflatus* . Global Change Biology, 22(12), 3888–3900. 10.1111/gcb.13350 27279327

[jeb13735-bib-0092] Munday, P. L. , Warner, R. R. , Monro, K. , Pandolfi, J. M. , & Marshall, D. J. (2013). Predicting evolutionary responses to climate change in the sea. Ecology Letters, 16(12), 1488–1500. 10.1111/ele.12185 24119205

[jeb13735-bib-0093] Murphy, D. W. , Adhikari, D. , Webster, D. R. , & Yen, J. (2016). Underwater flight by the planktonic sea butterfly. Journal of Experimental Biology, 219(4), 535–543. 10.1242/jeb.129205 26889002

[jeb13735-bib-0094] Ng, T. P. T. , Saltin, S. H. , Davies, M. S. , Johannesson, K. , Stafford, R. , & Williams, G. A. (2013). Snails and their trails: The multiple functions of trail‐following in gastropods. Biological Reviews, 88(3), 683–700. 10.1111/brv.12023 23374161

[jeb13735-bib-0095] Nielsen, E. E. , Hemmer‐Hansen, J. , Larsen, P. F. , & Bekkevold, D. (2009). Population genomics of marine fishes: Identifying adaptive variation in space and time. Molecular Ecology, 18(15), 3128–3150. 10.1111/j.1365-294X.2009.04272.x 19627488

[jeb13735-bib-0096] Norris, R. D. (2000). Pelagic species diversity, biogeography, and evolution. Paleobiology, 26(S4), 236–258. 10.1017/S0094837300026956

[jeb13735-bib-0097] Norton, E. L. , & Goetze, E. (2013). Equatorial dispersal barriers and limited population connectivity among oceans in a planktonic copepod. Limnology and Oceanography, 58(5), 1581–1596. 10.4319/lo.2013.58.5.1581

[jeb13735-bib-0098] O'Dea, A. , Lessios, H. A. , Coates, A. G. , Eytan, R. I. , Restrepo‐Moreno, S. A. , Cione, A. L. , Collins, L. S. , de Queiroz, A. , Farris, D. W. , Norris, R. D. , Stallard, R. F. , Woodburne, M. O. , Aguilera, O. , Aubry, M.‐P. , Berggren, W. A. , Budd, A. F. , Cozzuol, M. A. , Coppard, S. E. , Duque‐Caro, H. , … Jackson, J. B. C. (2016). Formation of the isthmus of Panama. Science Advances, 2(8), 1–12. 10.1126/sciadv.1600883 PMC4988774 27540590

[jeb13735-bib-0099] Oksanen, J. , Guillaume Blanchet, F. , Friendly, M. , Kindt, R. , Legendre, P. , McGlinn, D. , Minchin, P. R. , O'Hara, R. B. , Simpson, G. L. , Solymos, P. , Henry, M. , Stevens, H. , , & Wagner, H. (2019). vegan: Community ecology package R package version 2.5‐6. Retrieved from https://CRAN.R‐project.org/package=vegan

[jeb13735-bib-0100] Padgham, M. , & Sumner, M. D. (2020). geodist: Fast, dependency‐free geodesic distance calculations R package version 0.0.4. Retrieved from https://cran.r‐project.org/package=geodist

[jeb13735-bib-0101] Padial, J. M. , Miralles, A. , De la Riva, I. , & Vences, M. (2010). The integrative future of taxonomy. Frontiers in Zoology, 7, 1–14. 10.1186/1742-9994-7-16 20500846 PMC2890416

[jeb13735-bib-0102] Peijnenburg, K. T. C. A. , Breeuwer, J. A. J. , Pierrot‐Bults, A. C. , & Menken, S. B. J. (2004). Phylogeography of the planktonic chaetognath *Sagitta setosa* reveals isolation in European seas. Evolution, 58(7), 1472–1487. 10.1111/j.0014-3820.2004.tb01728.x 15341150

[jeb13735-bib-0103] Peijnenburg, K. T. C. A. , & Goetze, E. (2013). High evolutionary potential of marine zooplankton. Ecology and Evolution, 3(8), 2765–2783. 10.1002/ece3.644 24567838 PMC3930040

[jeb13735-bib-0104] Peijnenburg, K. T. C. A. , Janssen, A. W. , Wall‐Palmer, D. , Goetze, E. , Maas, A. E. , Todd, J. A. , & Marlétaz, F. (2020). The origin and diversification of pteropods precede past perturbations in the Earth's carbon cycle. Proceedings of the National Academy of Sciences of the United States of America.117(41), 25609–25617. 10.1073/pnas.1920918117 32973093 PMC7568333

[jeb13735-bib-0105] Pierrot‐Bults, A. C. , & Van der Spoel, S. (1979). Speciation in macrozooplankton In Zoogeography and diversity in Plankton (Vol. 9, pp. 144–167). Halsted.

[jeb13735-bib-0106] Pineda, J. , Reyns, N. B. , & Starczak, V. R. (2009). Complexity and simplification in understanding recruitment in benthic populations. Population Ecology, 51(1), 17–32. 10.1007/s10144-008-0118-0

[jeb13735-bib-0107] Poloczanska, E. S. , Burrows, M. T. , Brown, C. J. , García Molinos, J. , Halpern, B. S. , Hoegh‐Guldberg, O. , Kappel, C. V. , Moore, P. J. , Richardson, A. J. , Schoeman, D. S. , & Sydeman, W. J. (2016). Responses of marine organisms to climate change across oceans. Frontiers in Marine Science, 3(62), 1–21. 10.3389/fmars.2016.00062

[jeb13735-bib-0108] Postel, U. , Glemser, B. , Salazar Alekseyeva, K. , Eggers, S. L. , Groth, M. , Glöckner, G. , John, U. , Mock, T. , Klemm, K. , Valentin, K. , & Beszteri, B. (2020). Adaptive divergence across Southern Ocean gradients in the pelagic diatom *Fragilariopsis kerguelensis* . Molecular Ecology, 1–12. 10.1111/mec.15554 32672394

[jeb13735-bib-0109] Prairie, J. C. , Sutherland, K. R. , Nickols, K. J. , & Kaltenberg, A. M. (2012). Biophysical interactions in the plankton: A cross‐scale review. Limnology and Oceanography: Fluids and Environments, 2(1), 121–145. 10.1215/21573689-1964713

[jeb13735-bib-0110] Prazeres, M. , Morard, R. , Roberts, T. E. , Doo, S. S. , Jompa, J. , Schmidt, C. , Stuhr, M. , Renema, W. , & Kucera, M. (2020). High dispersal capacity and biogeographic breaks shape the genetic diversity of a globally‐distributed reef‐dwelling calcifier. Ecology and Evolution, 10(12), 5976–5989. 10.1002/ece3.6335 32607205 PMC7319125

[jeb13735-bib-0111] R Core Team (2017). R: A language and environment for statistical computing, Vienna, Austria: R Foundation for Statistical Computing Retrieved from https://www.r‐project.org/

[jeb13735-bib-0112] Reid, D. G. , Lal, K. , Mackenzie‐Dodds, J. , Kaligis, F. , Littlewood, D. T. J. , & Williams, S. T. (2006). Comparative phylogeography and species boundaries in *Echinolittorina* snails in the central Indo‐West Pacific. Journal of Biogeography, 33(6), 990–1006. 10.1111/j.1365-2699.2006.01469.x

[jeb13735-bib-0113] Reygondeau, G. , Longhurst, A. , Martinez, E. , Beaugrand, G. , Antoine, D. , & Maury, O. (2013). Dynamic biogeochemical provinces in the global ocean. Global Biogeochemical Cycles, 27(4), 1046–1058. 10.1002/gbc.20089

[jeb13735-bib-0114] Reygondeau, G. , Maury, O. , Beaugrand, G. , Fromentin, J. M. , Fonteneau, A. , & Cury, P. (2012). Biogeography of tuna and billfish communities. Journal of Biogeography, 39(1), 114–129. 10.1111/j.1365-2699.2011.02582.x

[jeb13735-bib-0115] Rice, W. R. (1989). Analyzing tables of statistical tests. Evolution, 43(1), 223–225. 10.1111/j.1558-5646.1989.tb04220.x 28568501

[jeb13735-bib-0116] Riebesell, U. , & Gattuso, J. P. (2015). Lessons learned from ocean acidification research. Nature Climate Change, 5(1), 12–14. 10.1038/nclimate2456

[jeb13735-bib-0117] Ritcher, G. (1979). Die thecosomen Pteropoden der “Meteor”‐Expedition in den Indischen Ozean 1964/65. Forsch. Ergenbnisse, 29(D), 1–29.

[jeb13735-bib-0118] Rohlf, F. J. (2015). The tps series of software. Hystrix, 26(1), 9–12. 10.4404/hystrix-26.1-11264

[jeb13735-bib-0119] Rohlf, F. J. , & Slice, D. (1990). Extension of the procrustes method for the optimal superimposition of landmarks. Systematic Zoology, 39, 40–59.

[jeb13735-bib-0120] Rozas, J. , Ferrer‐Mata, A. , Sanchez‐DelBarrio, J. C. , Guirao‐Rico, S. , Librado, P. , Ramos‐Onsins, S. E. , & Sanchez‐Gracia, A. (2017). DnaSP 6: DNA sequence polymorphism analysis of large data sets. Molecular Biology and Evolution, 34(12), 3299–3302. 10.1093/molbev/msx248 29029172

[jeb13735-bib-0121] Sanford, E. , & Kelly, M. W. (2011). Local Adaptation in Marine Invertebrates. Annual Review of Marine Science, 3(1), 509–535. 10.1146/annurev-marine-120709-142756 21329215

[jeb13735-bib-0122] Schluter, D. (2009). Evidence for ecological speciation and its alternative. Science, 323(5915), 737–741. 10.1126/science.1160006 19197053

[jeb13735-bib-0123] Shimizu, K. , Kimoto, K. , Noshita, K. , Wakita, M. , Fujiki, T. , & Sasaki, T. (2018). Phylogeography of the pelagic snail *Limacina helicina* (Gastropoda: Thecosomata) in the subarctic western North Pacific. Journal of Molluscan Studies, 84(1), 30–37. 10.1093/mollus/eyx040

[jeb13735-bib-0124] Sromek, L. , Lasota, R. , & Wolowicz, M. (2015). Impact of glaciations on genetic diversity of pelagic mollusks: Antarctic *Limacina antarctica* and Arctic *Limacina helicina* . Marine Ecology Progress Series, 525, 143–152. 10.3354/meps11237

[jeb13735-bib-0125] Stephens, M. , & Donnelly, P. (2003). A comparison of Bayesian methods for haplotype reconstruction from population genotype data. American Journal of Human Genetics, 73(5), 1162–1169. 10.1086/379378 14574645 PMC1180495

[jeb13735-bib-0126] Sunday, J. M. , Calosi, P. , Dupont, S. , Munday, P. L. , Stillman, J. H. , & Reusch, T. B. H. (2014). Evolution in an acidifying ocean. Trends in Ecology and Evolution, 29(2), 117–125. 10.1016/j.tree.2013.11.001 24355315

[jeb13735-bib-0127] Sutton, T. T. , Clark, M. R. , Dunn, D. C. , Halpin, P. N. , Rogers, A. D. , Guinotte, J. , Bograd, S. J. , Angel, M. V. , Perez, J. A. A. , Wishner, K. , Haedrich, R. L. , Lindsay, D. J. , Drazen, J. C. , Vereshchaka, A. , Piatkowski, U. , Morato, T. , Błachowiak‐Samołyk, K. , Robison, B. H. , Gjerde, K. M. , … Heino, M. (2017). A global biogeographic classification of the mesopelagic zone. Deep Sea Research Part I: Oceanographic Research Papers, 126, 85–102. 10.1016/j.dsr.2017.05.006

[jeb13735-bib-0128] Tajima, F. (1989). Statistical method for testing the neutral mutation hypothesis by DNA polymorphism. Genetics, 123, 585–595. 2513255 10.1093/genetics/123.3.585 PMC1203831

[jeb13735-bib-0129] Teske, P. R. , Golla, T. R. , Sandoval‐Castillo, J. , Emami‐Khoyi, A. , van der Lingen, C. D. , von der Heyden, S. , Chiazzari, B. , Jansen van Vuuren, B. , & Beheregaray, L. B. (2018). Mitochondrial DNA is unsuitable to test for isolation by distance. Scientific Reports, 8(1), 1–9. 10.1038/s41598-018-25138-9 29855482 PMC5981212

[jeb13735-bib-0130] van der Spoel, S. , Newman, L. J. , & Estep, K. W. (1997). Pelagic molluscs of the world. Retrieved from http://species‐identification.org/species.php?species_group=pelagic_molluscs&menuentry=soorten ETI World Biodiversity Database CD‐ROM series, Amsterdam: Expert‐Center for Taxonomic Identification http://species‐identification.org/

[jeb13735-bib-0131] Vonnemann, V. , Schrödl, M. , Klussmann‐Kolb, A. , & Wägele, H. (2005). Reconstruction of the phylogeny of the opisthobranchia (Mollusca: Gastropoda) by means of 18s and 28s rRNA gene sequences. Journal of Molluscan Studies, 71(2), 113–125. 10.1093/mollus/eyi014

[jeb13735-bib-0132] Wall‐Palmer, D. , Burridge, A. K. , Goetze, E. , Stokvis, F. R. , Janssen, A. W. , Mekkes, L. , Moreno‐Alcántara, M. , Bednaršek, N. , Schiøtte, T. , Sørensen, M. V. , Smart, C. W. , & Peijnenburg, K. T. C. A. (2018). Biogeography and genetic diversity of the atlantid heteropods. Progress in Oceanography, 160, 1–25. 10.1016/j.pocean.2017.11.004 29479121 PMC5819870

[jeb13735-bib-0133] Wall‐Palmer, D. , Burridge, A. K. , Peijnenburg, K. T. C. A. , Janssen, A. , Goetze, E. , Kirby, R. , Hart, M. B. , & Smart, C. W. (2016). Evidence for the validity of *Protatlanta sculpta* (Gastropoda: Pterotracheoidea). Contributions to Zoology, 85(4), 423–435. 10.1163/18759866-08504003

[jeb13735-bib-0134] Weersing, K. , & Toonen, R. J. (2009). Population genetics, larval dispersal, and connectivity in marine systems. Marine Ecology Progress Series, 393, 1–12. 10.3354/meps08287

[jeb13735-bib-0135] Wells, F. E. (1976). Growth rate of four species of euthecosomatous pteropods occuring off Barbados, West Indies. Nautilus, 90, 114–116.

[jeb13735-bib-0136] Westram, A. M. , Rafajlović, M. , Chaube, P. , Faria, R. , Larsson, T. , Panova, M. , Ravinet, M. , Blomberg, A. , Mehlig, B. , Johannesson, K. , & Butlin, R. (2018). Clines on the seashore: The genomic architecture underlying rapid divergence in the face of gene flow. Evolution Letters, 2(4), 297–309. 10.1002/evl3.74 30283683 PMC6121805

[jeb13735-bib-0137] Whittaker, K. A. , & Rynearson, T. A. (2017). Evidence for environmental and ecological selection in a microbe with no geographic limits to gene flow. Proceedings of the National Academy of Sciences of the United States of America, 114(10), 2651–2656. 10.1073/pnas.1612346114 28209775 PMC5347567

[jeb13735-bib-0138] Wickham, H. (2016). ggplot2: Elegant graphics for data analysis. Springer‐Verlag Retrieved from https://ggplot2.tidyverse.org

[jeb13735-bib-0139] Zelditch, M. L. , Swiderski, D. L. , Sheets, H. D. , & Fink, W. L. (2004). Geometric morphometrics for biologists: A primer, 1, Elsevier Academic Press.

