# Supplementary material for: Oceanic dispersal barriers in a holoplanktonic gastropod
Source: J Evol Biol. 2020 Nov 21;34(1):224–40. doi: 10.1111/jeb.13735 (PMC7894488; doi:10.1111/jeb.13735)
Supplement: Supplementary file 1 — Appendix S1 [file JEB-34-224-s001.docx]

**Appendix S1**

**Supplementary material: Oceanic dispersal barriers in a holoplanktonic gastropod**

**
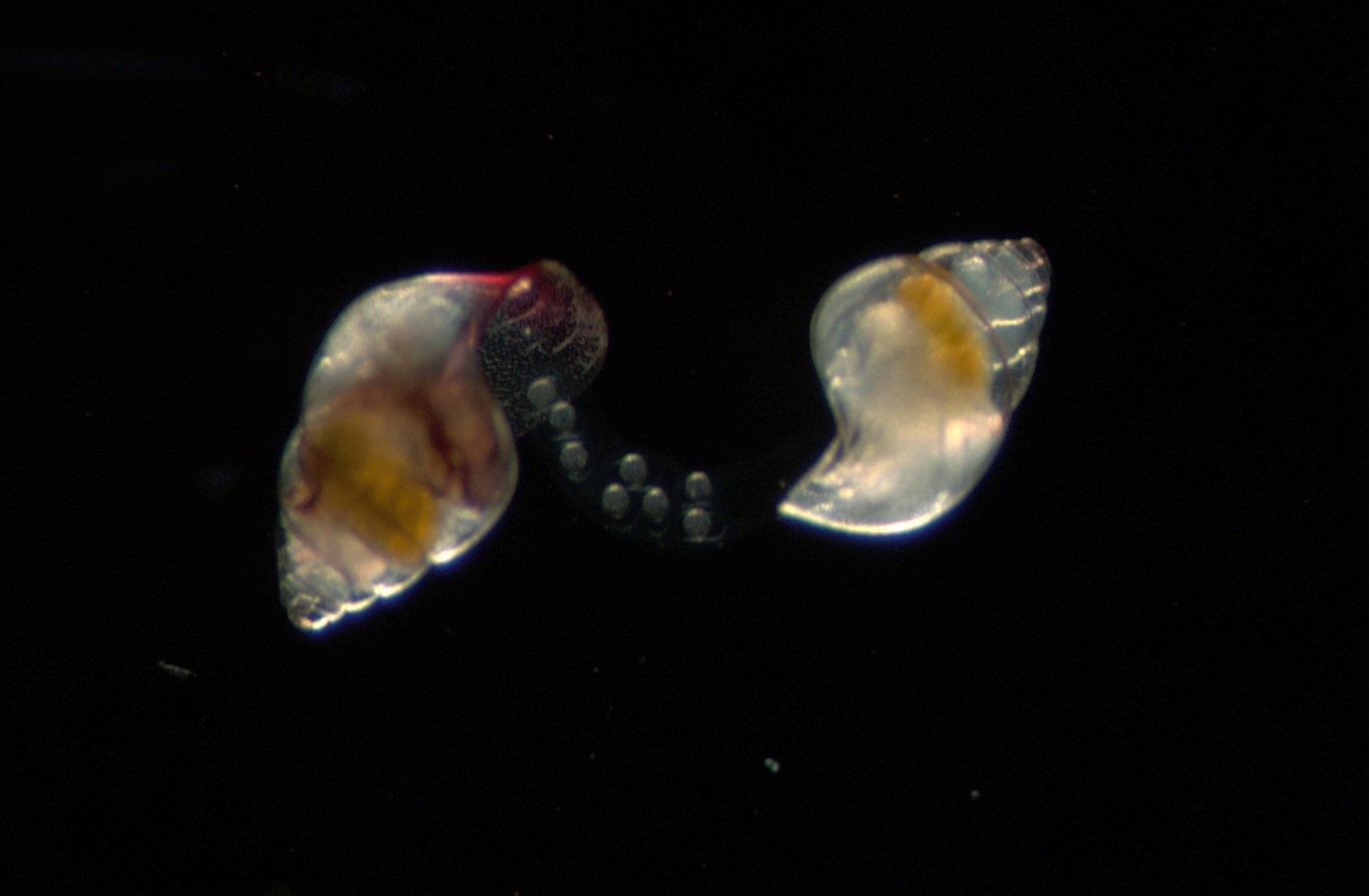
**

**Fig. S1** Two individuals of *Limacina. bulimoides*, with left individual laying free-floating egg strings, sampled from the North Atlantic (32°52.92 N, 26° 53.94 W) during the AMT27 cruise in 2017. See also Supplementary Video 1 which shows an individual of *L. bulimoides* (separate individual from this picture) in the process of laying egg strings.


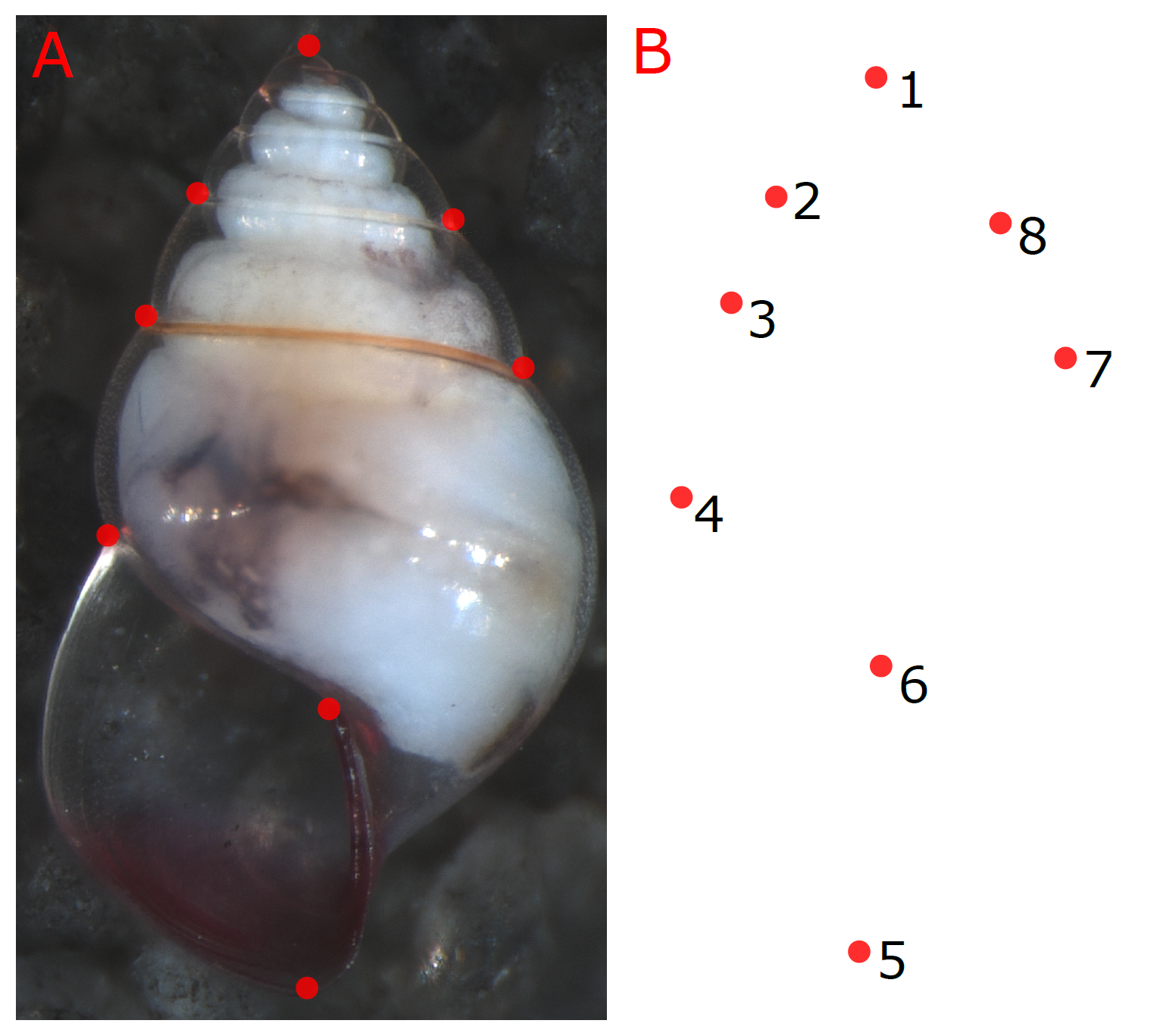


**Fig. S2** Representation of (semi-) landmark placement for 2D geometric morphometric analysis of shell shape for *Limacina bulimoides*. (A) The position of eight (semi-) landmarks on a shell. (B) Consensus plot of the shell shape calculated by the (semi-) landmark positions on 136 shells.

**
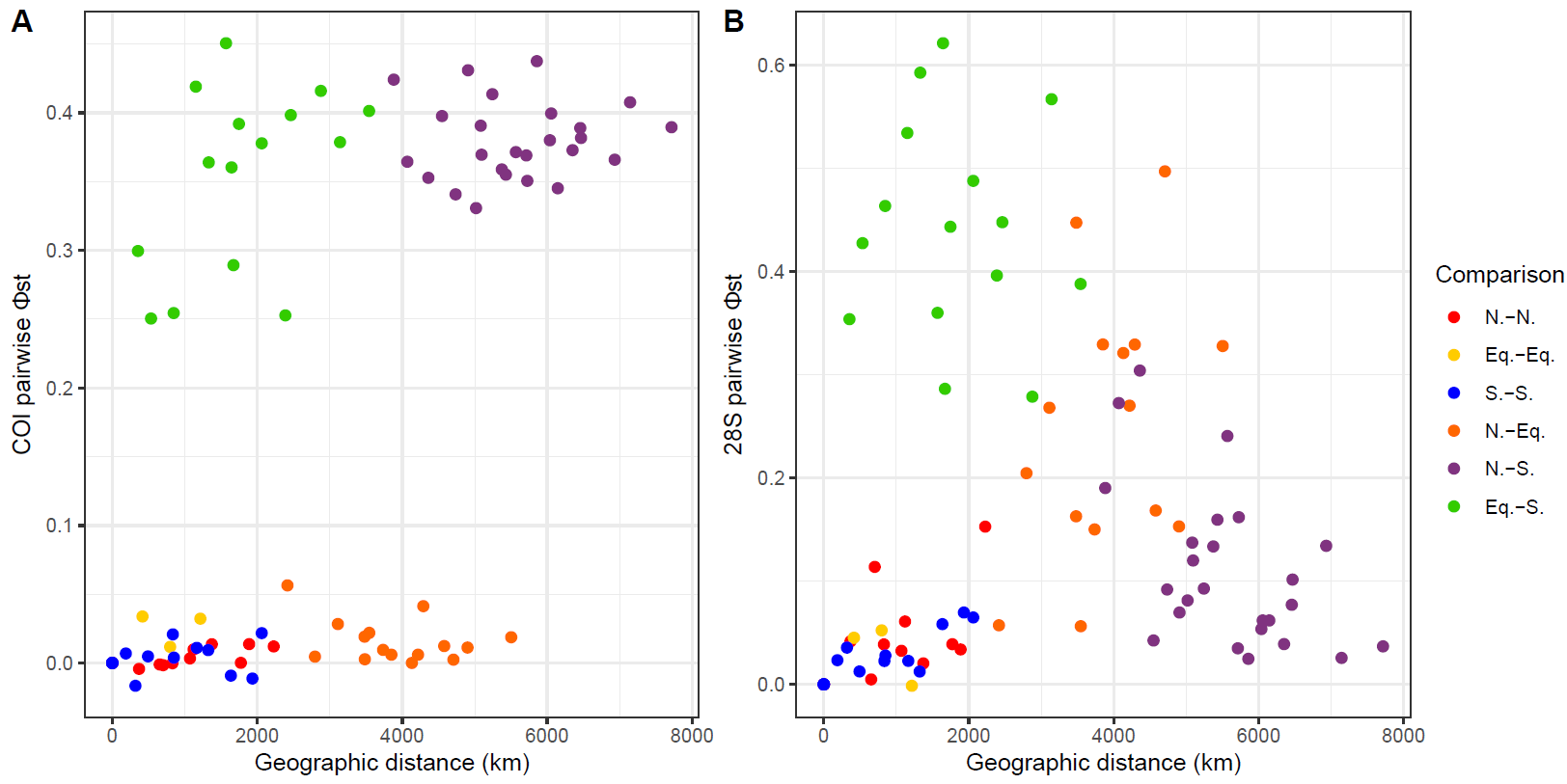
**

**Fig. S3** Scatter plots of genetic differentiation between Atlantic samples of *Limacina bulimoides* (pairwise Φ_ST_) and geographic distance for A) mitochondrial cytochrome *c* oxidase I (COI) and B) nuclear 28S rDNA (28S). Comparisons within and between population groups are indicated with the following colour scheme: within North: red, within Equatorial: yellow, within South: blue, between North and Equatorial: orange, between North and South: purple, between Equatorial and South: green (see legend).


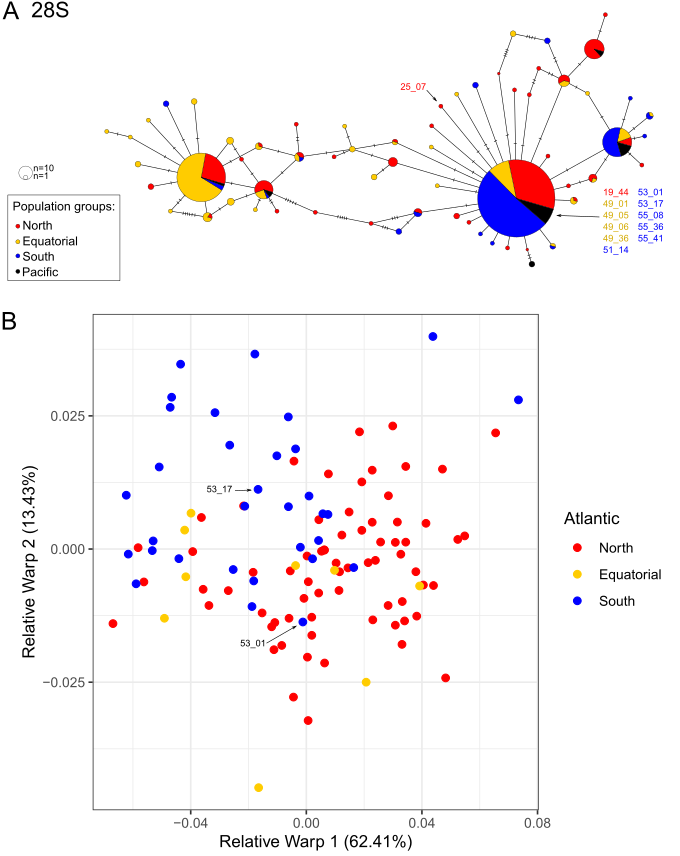


**Fig. S4** Information about the nuclear 28S haplotype (A) and morphotype (B) of the expatriates identified based on mitochondrial haplogroups (see also Table S3). 28S haplotype data was available for 12 individuals (A) but only two individuals were included in the morphometric analysis (B).


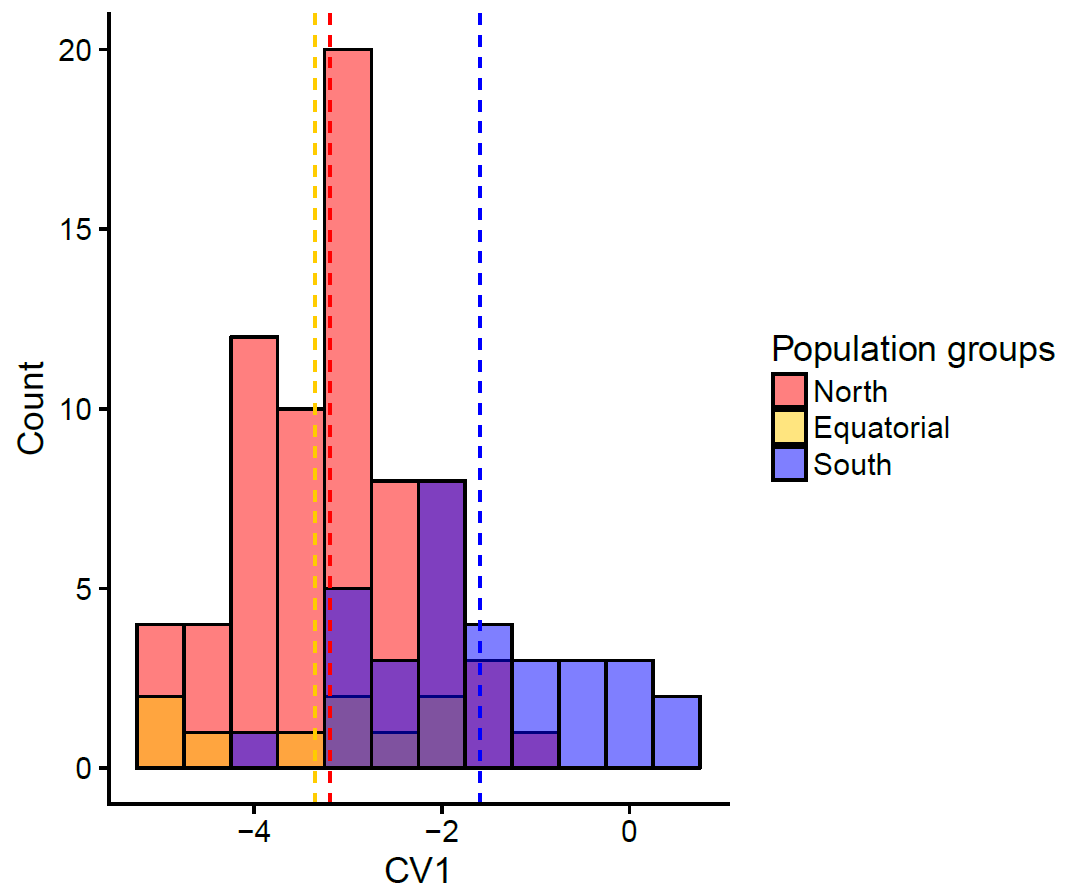


**Fig. S5** Canonical variate analysis of shell shape variation based on the repeatable Relative Warps (RWs), consisting of centroid size, RW1, RW2, RW4, RW6 and RW7. Shell shape variation between the Atlantic stations North (stations 13-29), Equatorial (43-45) and South (51-60) clades is maximised along the canonical variate 1 (CV1) axis. Group means for each group are indicated by dotted lines with the respective colour on the CV1 axis. Results of multiple pairwise comparisons of the means are reported in Table S5.

**Table S1** Specimen information for the 413 *Limacina bulimoides* individuals included in this study, including specimen voucher, specimen ID, location, collection date, NCBI accession numbers for mitochondrial cytochrome *c* oxidase I (COI) and nuclear 28S rDNA (28S) genes, and if it was included in the morphometrics dataset.

| **Specimen voucher** | **Specimen ID** | **Ocean** | **Station** | **Latitude** | **Longitude** | **Collection date** | **COI** | **28S** | **Included in morphometrics** |
| --- | --- | --- | --- | --- | --- | --- | --- | --- | --- |
| RMNH.MOL.342218 | Lbul_AMT22_13_01 | Atlantic | AMT22_13 | 34.37 | -27.63 | 18/10/2012 | MN952611 | MN950433 |  |
| RMNH.MOL.342219 | Lbul_AMT22_13_02 | Atlantic | AMT22_13 | 34.37 | -27.63 | 18/10/2012 | MN952612 | MN950434 |  |
| RMNH.MOL.342220 | Lbul_AMT22_13_03 | Atlantic | AMT22_13 | 34.37 | -27.63 | 18/10/2012 | MN952613 | MN950435 |  |
| RMNH.MOL.342221 | Lbul_AMT22_13_04 | Atlantic | AMT22_13 | 34.37 | -27.63 | 18/10/2012 | MN952614 | MN950436 |  |
| RMNH.MOL.342222 | Lbul_AMT22_13_05 | Atlantic | AMT22_13 | 34.37 | -27.63 | 18/10/2012 | MN952615 | MN950437 |  |
| RMNH.MOL.342223 | Lbul_AMT22_13_09 | Atlantic | AMT22_13 | 34.37 | -27.63 | 18/10/2012 | MN952616 | MN950438 |  |
| RMNH.MOL.342224 | Lbul_AMT22_13_10 | Atlantic | AMT22_13 | 34.37 | -27.63 | 18/10/2012 | MN952617 | MN950439 |  |
| RMNH.MOL.342225 | Lbul_AMT22_13_14 | Atlantic | AMT22_13 | 34.37 | -27.63 | 18/10/2012 | MN952618 | MN950440 |  |
| RMNH.MOL.342226 | Lbul_AMT22_13_15 | Atlantic | AMT22_13 | 34.37 | -27.63 | 18/10/2012 | MN952619 | MN950441 |  |
| RMNH.MOL.342227 | Lbul_AMT22_13_16 | Atlantic | AMT22_13 | 34.37 | -27.63 | 18/10/2012 | MN952620 | MN950442 |  |
| RMNH.MOL.342228 | Lbul_AMT22_13_20 | Atlantic | AMT22_13 | 34.37 | -27.63 | 18/10/2012 | MN952621 | MN950443 |  |
| RMNH.MOL.342229 | Lbul_AMT22_13_21 | Atlantic | AMT22_13 | 34.37 | -27.63 | 18/10/2012 | MN952622 | MN950444 |  |
| RMNH.MOL.342230 | Lbul_AMT22_13_23 | Atlantic | AMT22_13 | 34.37 | -27.63 | 18/10/2012 | MN952623 | MN950445 |  |
| RMNH.MOL.342231 | Lbul_AMT22_13_24 | Atlantic | AMT22_13 | 34.37 | -27.63 | 18/10/2012 | MN952624 | MN950446 |  |
| RMNH.MOL.342232 | Lbul_AMT22_13_25 | Atlantic | AMT22_13 | 34.37 | -27.63 | 18/10/2012 | MN952625 | MN950447 |  |
| RMNH.MOL.342233 | Lbul_AMT22_13_26 | Atlantic | AMT22_13 | 34.37 | -27.63 | 18/10/2012 | MN952626 | MN950448 |  |
| RMNH.MOL.342234 | Lbul_AMT22_13_28 | Atlantic | AMT22_13 | 34.37 | -27.63 | 18/10/2012 | MN952627 | MN950449 | Yes |
| RMNH.MOL.342235 | Lbul_AMT22_13_29 | Atlantic | AMT22_13 | 34.37 | -27.63 | 18/10/2012 | MN952628 | MN950450 | Yes |
| RMNH.MOL.342236 | Lbul_AMT22_13_30 | Atlantic | AMT22_13 | 34.37 | -27.63 | 18/10/2012 |  |  | Yes |
| RMNH.MOL.342237 | Lbul_AMT22_13_31 | Atlantic | AMT22_13 | 34.37 | -27.63 | 18/10/2012 | MN952629 | MN950451 | Yes |
| RMNH.MOL.342238 | Lbul_AMT22_13_32 | Atlantic | AMT22_13 | 34.37 | -27.63 | 18/10/2012 |  |  | Yes |
| RMNH.MOL.342239 | Lbul_AMT22_13_33 | Atlantic | AMT22_13 | 34.37 | -27.63 | 18/10/2012 | MN952630 | MN950452 |  |
| RMNH.MOL.342240 | Lbul_AMT22_13_34 | Atlantic | AMT22_13 | 34.37 | -27.63 | 18/10/2012 |  |  | Yes |
| RMNH.MOL.342241 | Lbul_AMT22_13_35 | Atlantic | AMT22_13 | 34.37 | -27.63 | 18/10/2012 | MN952631 | MN950453 | Yes |
| RMNH.MOL.342242 | Lbul_AMT22_13_36 | Atlantic | AMT22_13 | 34.37 | -27.63 | 18/10/2012 | MN952632 | MN950454 |  |
| RMNH.MOL.342243 | Lbul_AMT22_13_37 | Atlantic | AMT22_13 | 34.37 | -27.63 | 18/10/2012 | MN952633 | MN950455 |  |
| RMNH.MOL.342244 | Lbul_AMT22_13_38 | Atlantic | AMT22_13 | 34.37 | -27.63 | 18/10/2012 | MN952634 | MN950735 | Yes |
| RMNH.MOL.342245 | Lbul_AMT22_13_39 | Atlantic | AMT22_13 | 34.37 | -27.63 | 18/10/2012 | MN952635 | MN950456 | Yes |
| RMNH.MOL.342246 | Lbul_AMT22_13_40 | Atlantic | AMT22_13 | 34.37 | -27.63 | 18/10/2012 | MN952636 | MN950736 | Yes |
| RMNH.MOL.342247 | Lbul_AMT22_13_41 | Atlantic | AMT22_13 | 34.37 | -27.63 | 18/10/2012 | MN952637 | MN950737 |  |
| RMNH.MOL.342248 | Lbul_AMT22_13_42 | Atlantic | AMT22_13 | 34.37 | -27.63 | 18/10/2012 | MN952638 | MN950738 |  |
| RMNH.MOL.342249 | Lbul_AMT22_19_03 | Atlantic | AMT22_19 | 27.60 | -36.37 | 21/10/2012 |  |  | Yes |
| RMNH.MOL.342250 | Lbul_AMT22_19_06 | Atlantic | AMT22_19 | 27.60 | -36.37 | 21/10/2012 | MN952639 | MN950457 |  |
| RMNH.MOL.342251 | Lbul_AMT22_19_09 | Atlantic | AMT22_19 | 27.60 | -36.37 | 21/10/2012 | MN952640 | MN950458 | Yes |
| RMNH.MOL.342252 | Lbul_AMT22_19_10 | Atlantic | AMT22_19 | 27.60 | -36.37 | 21/10/2012 | MN952641 | MN950459 |  |
| RMNH.MOL.342253 | Lbul_AMT22_19_11 | Atlantic | AMT22_19 | 27.60 | -36.37 | 21/10/2012 | MN952642 | MN950460 |  |
| RMNH.MOL.342254 | Lbul_AMT22_19_12 | Atlantic | AMT22_19 | 27.60 | -36.37 | 21/10/2012 | MN952643 | MN950461 |  |
| RMNH.MOL.342255 | Lbul_AMT22_19_13 | Atlantic | AMT22_19 | 27.60 | -36.37 | 21/10/2012 | MN952644 | MN950462 |  |
| RMNH.MOL.342256 | Lbul_AMT22_19_14 | Atlantic | AMT22_19 | 27.60 | -36.37 | 21/10/2012 | MN952645 | MN950463 |  |
| RMNH.MOL.342257 | Lbul_AMT22_19_15 | Atlantic | AMT22_19 | 27.60 | -36.37 | 21/10/2012 | MN952646 | MN950464 |  |
| RMNH.MOL.342258 | Lbul_AMT22_19_16 | Atlantic | AMT22_19 | 27.60 | -36.37 | 21/10/2012 | MN952647 | MN950465 |  |
| RMNH.MOL.342259 | Lbul_AMT22_19_17 | Atlantic | AMT22_19 | 27.60 | -36.37 | 21/10/2012 | MN952648 | MN950466 |  |
| RMNH.MOL.342260 | Lbul_AMT22_19_18 | Atlantic | AMT22_19 | 27.60 | -36.37 | 21/10/2012 | MN952649 | MN950467 |  |
| RMNH.MOL.342261 | Lbul_AMT22_19_19 | Atlantic | AMT22_19 | 27.60 | -36.37 | 21/10/2012 | MN952650 | MN950468 |  |
| RMNH.MOL.342262 | Lbul_AMT22_19_20 | Atlantic | AMT22_19 | 27.60 | -36.37 | 21/10/2012 | MN952651 |  |  |
| RMNH.MOL.342263 | Lbul_AMT22_19_21 | Atlantic | AMT22_19 | 27.60 | -36.37 | 21/10/2012 | MN952652 | MN950469 |  |
| RMNH.MOL.342264 | Lbul_AMT22_19_22 | Atlantic | AMT22_19 | 27.60 | -36.37 | 21/10/2012 | MN952653 | MN950470 |  |
| RMNH.MOL.342265 | Lbul_AMT22_19_23 | Atlantic | AMT22_19 | 27.60 | -36.37 | 21/10/2012 | MN952654 | MN950471 |  |
| RMNH.MOL.342266 | Lbul_AMT22_19_24 | Atlantic | AMT22_19 | 27.60 | -36.37 | 21/10/2012 | MN952655 | MN950472 |  |
| RMNH.MOL.342267 | Lbul_AMT22_19_25 | Atlantic | AMT22_19 | 27.60 | -36.37 | 21/10/2012 | MN952656 | MN950473 |  |
| RMNH.MOL.342268 | Lbul_AMT22_19_26 | Atlantic | AMT22_19 | 27.60 | -36.37 | 21/10/2012 | MN952657 | MN950474 |  |
| RMNH.MOL.342269 | Lbul_AMT22_19_27 | Atlantic | AMT22_19 | 27.60 | -36.37 | 21/10/2012 | MN952658 | MN950475 |  |
| RMNH.MOL.342270 | Lbul_AMT22_19_28 | Atlantic | AMT22_19 | 27.60 | -36.37 | 21/10/2012 |  |  | Yes |
| RMNH.MOL.342271 | Lbul_AMT22_19_29 | Atlantic | AMT22_19 | 27.60 | -36.37 | 21/10/2012 |  |  | Yes |
| RMNH.MOL.342272 | Lbul_AMT22_19_30 | Atlantic | AMT22_19 | 27.60 | -36.37 | 21/10/2012 |  |  | Yes |
| RMNH.MOL.342273 | Lbul_AMT22_19_31 | Atlantic | AMT22_19 | 27.60 | -36.37 | 21/10/2012 | MN952659 | MN950476 |  |
| RMNH.MOL.342274 | Lbul_AMT22_19_32 | Atlantic | AMT22_19 | 27.60 | -36.37 | 21/10/2012 |  |  | Yes |
| RMNH.MOL.342275 | Lbul_AMT22_19_33 | Atlantic | AMT22_19 | 27.60 | -36.37 | 21/10/2012 | MN952660 | MN950477 | Yes |
| RMNH.MOL.342276 | Lbul_AMT22_19_34 | Atlantic | AMT22_19 | 27.60 | -36.37 | 21/10/2012 | MN952661 | MN950478 | Yes |
| RMNH.MOL.342277 | Lbul_AMT22_19_35 | Atlantic | AMT22_19 | 27.60 | -36.37 | 21/10/2012 | MN952662 |  | Yes |
| RMNH.MOL.342278 | Lbul_AMT22_19_36 | Atlantic | AMT22_19 | 27.60 | -36.37 | 21/10/2012 | MN952663 | MN950479 | Yes |
| RMNH.MOL.342279 | Lbul_AMT22_19_37 | Atlantic | AMT22_19 | 27.60 | -36.37 | 21/10/2012 |  |  | Yes |
| RMNH.MOL.342280 | Lbul_AMT22_19_38 | Atlantic | AMT22_19 | 27.60 | -36.37 | 21/10/2012 | MN952664 |  | Yes |
| RMNH.MOL.342281 | Lbul_AMT22_19_39 | Atlantic | AMT22_19 | 27.60 | -36.37 | 21/10/2012 | MN952665 |  | Yes |
| RMNH.MOL.342282 | Lbul_AMT22_19_40 | Atlantic | AMT22_19 | 27.60 | -36.37 | 21/10/2012 | MN952666 |  | Yes |
| RMNH.MOL.342283 | Lbul_AMT22_19_41 | Atlantic | AMT22_19 | 27.60 | -36.37 | 21/10/2012 | MN952667 | MN950480 | Yes |
| RMNH.MOL.342284 | Lbul_AMT22_19_42 | Atlantic | AMT22_19 | 27.60 | -36.37 | 21/10/2012 |  |  | Yes |
| RMNH.MOL.342285 | Lbul_AMT22_19_44 | Atlantic | AMT22_19 | 27.60 | -36.37 | 21/10/2012 | MN952668 | MN950481 |  |
| RMNH.MOL.342286 | Lbul_AMT22_19_45 | Atlantic | AMT22_19 | 27.60 | -36.37 | 21/10/2012 | MN952669 | MN950482 | Yes |
| RMNH.MOL.342287 | Lbul_AMT22_23_01 | Atlantic | AMT22_23 | 23.15 | -40.63 | 23/10/2012 | MN952670 |  |  |
| RMNH.MOL.342288 | Lbul_AMT22_23_02 | Atlantic | AMT22_23 | 23.15 | -40.63 | 23/10/2012 | MN952671 | MN950483 |  |
| RMNH.MOL.342289 | Lbul_AMT22_23_03 | Atlantic | AMT22_23 | 23.15 | -40.63 | 23/10/2012 | MN952672 | MN950484 |  |
| RMNH.MOL.342290 | Lbul_AMT22_23_04 | Atlantic | AMT22_23 | 23.15 | -40.63 | 23/10/2012 | MN952673 | MN950485 |  |
| RMNH.MOL.342291 | Lbul_AMT22_23_06 | Atlantic | AMT22_23 | 23.15 | -40.63 | 23/10/2012 | MN952674 |  |  |
| RMNH.MOL.342292 | Lbul_AMT22_23_07 | Atlantic | AMT22_23 | 23.15 | -40.63 | 23/10/2012 | MN952675 | MN950486 |  |
| RMNH.MOL.342293 | Lbul_AMT22_23_08 | Atlantic | AMT22_23 | 23.15 | -40.63 | 23/10/2012 | MN952676 | MN950487 |  |
| RMNH.MOL.342294 | Lbul_AMT22_23_09 | Atlantic | AMT22_23 | 23.15 | -40.63 | 23/10/2012 | MN952677 | MN950488 |  |
| RMNH.MOL.342295 | Lbul_AMT22_23_10 | Atlantic | AMT22_23 | 23.15 | -40.63 | 23/10/2012 | MN952678 | MN950489 |  |
| RMNH.MOL.342296 | Lbul_AMT22_23_11 | Atlantic | AMT22_23 | 23.15 | -40.63 | 23/10/2012 | MN952679 | MN950490 |  |
| RMNH.MOL.342297 | Lbul_AMT22_23_12 | Atlantic | AMT22_23 | 23.15 | -40.63 | 23/10/2012 | MN952680 | MN950491 |  |
| RMNH.MOL.342298 | Lbul_AMT22_23_13 | Atlantic | AMT22_23 | 23.15 | -40.63 | 23/10/2012 | MN952681 | MN950492 |  |
| RMNH.MOL.342299 | Lbul_AMT22_23_14 | Atlantic | AMT22_23 | 23.15 | -40.63 | 23/10/2012 | MN952682 | MN950493 |  |
| RMNH.MOL.342300 | Lbul_AMT22_23_17 | Atlantic | AMT22_23 | 23.15 | -40.63 | 23/10/2012 | MN952683 | MN950494 |  |
| RMNH.MOL.342301 | Lbul_AMT22_23_18 | Atlantic | AMT22_23 | 23.15 | -40.63 | 23/10/2012 | MN952684 | MN950495 |  |
| RMNH.MOL.342302 | Lbul_AMT22_23_19 | Atlantic | AMT22_23 | 23.15 | -40.63 | 23/10/2012 | MN952685 | MN950496 |  |
| RMNH.MOL.342303 | Lbul_AMT22_23_21 | Atlantic | AMT22_23 | 23.15 | -40.63 | 23/10/2012 | MN952686 | MN950497 |  |
| RMNH.MOL.342304 | Lbul_AMT22_23_22 | Atlantic | AMT22_23 | 23.15 | -40.63 | 23/10/2012 | MN952687 | MN950498 |  |
| RMNH.MOL.342305 | Lbul_AMT22_23_23 | Atlantic | AMT22_23 | 23.15 | -40.63 | 23/10/2012 | MN952688 |  |  |
| RMNH.MOL.342306 | Lbul_AMT22_23_24 | Atlantic | AMT22_23 | 23.15 | -40.63 | 23/10/2012 | MN952689 | MN950499 |  |
| RMNH.MOL.342307 | Lbul_AMT22_23_25 | Atlantic | AMT22_23 | 23.15 | -40.63 | 23/10/2012 |  |  | Yes |
| RMNH.MOL.342308 | Lbul_AMT22_23_26 | Atlantic | AMT22_23 | 23.15 | -40.63 | 23/10/2012 | MN952690 | MN950500 | Yes |
| RMNH.MOL.342309 | Lbul_AMT22_23_27 | Atlantic | AMT22_23 | 23.15 | -40.63 | 23/10/2012 | MN952691 | MN950501 |  |
| RMNH.MOL.342310 | Lbul_AMT22_23_28 | Atlantic | AMT22_23 | 23.15 | -40.63 | 23/10/2012 | MN952692 | MN950502 |  |
| RMNH.MOL.342311 | Lbul_AMT22_23_29 | Atlantic | AMT22_23 | 23.15 | -40.63 | 23/10/2012 | MN952693 | MN950503 |  |
| RMNH.MOL.342312 | Lbul_AMT22_23_30 | Atlantic | AMT22_23 | 23.15 | -40.63 | 23/10/2012 | MN952694 |  |  |
| RMNH.MOL.342313 | Lbul_AMT22_23_31 | Atlantic | AMT22_23 | 23.15 | -40.63 | 23/10/2012 | MN952695 | MN950504 |  |
| RMNH.MOL.342314 | Lbul_AMT22_23_32 | Atlantic | AMT22_23 | 23.15 | -40.63 | 23/10/2012 | MN952696 | MN950505 |  |
| RMNH.MOL.342315 | Lbul_AMT22_23_34 | Atlantic | AMT22_23 | 23.15 | -40.63 | 23/10/2012 | MN952697 | MN950506 |  |
| RMNH.MOL.342316 | Lbul_AMT22_25_02 | Atlantic | AMT22_25 | 20.40 | -38.62 | 24/10/2012 | MN952698 | MN950507 | Yes |
| RMNH.MOL.342317 | Lbul_AMT22_25_03 | Atlantic | AMT22_25 | 20.40 | -38.62 | 24/10/2012 | MN952699 | MN950508 | Yes |
| RMNH.MOL.342318 | Lbul_AMT22_25_04 | Atlantic | AMT22_25 | 20.40 | -38.62 | 24/10/2012 | MN952700 | MN950509 | Yes |
| RMNH.MOL.342319 | Lbul_AMT22_25_05 | Atlantic | AMT22_25 | 20.40 | -38.62 | 24/10/2012 | MN952701 | MN950510 |  |
| RMNH.MOL.342320 | Lbul_AMT22_25_06 | Atlantic | AMT22_25 | 20.40 | -38.62 | 24/10/2012 | MN952702 | MN950511 |  |
| RMNH.MOL.342321 | Lbul_AMT22_25_07 | Atlantic | AMT22_25 | 20.40 | -38.62 | 24/10/2012 | MN952703 | MN950739 |  |
| RMNH.MOL.342322 | Lbul_AMT22_25_08 | Atlantic | AMT22_25 | 20.40 | -38.62 | 24/10/2012 | MN952704 | MN950512 | Yes |
| RMNH.MOL.342323 | Lbul_AMT22_25_09 | Atlantic | AMT22_25 | 20.40 | -38.62 | 24/10/2012 |  | MN950740 |  |
| RMNH.MOL.342324 | Lbul_AMT22_25_10 | Atlantic | AMT22_25 | 20.40 | -38.62 | 24/10/2012 |  |  | Yes |
| RMNH.MOL.342325 | Lbul_AMT22_25_13 | Atlantic | AMT22_25 | 20.40 | -38.62 | 24/10/2012 | MN952705 | MN950513 | Yes |
| RMNH.MOL.342326 | Lbul_AMT22_25_14 | Atlantic | AMT22_25 | 20.40 | -38.62 | 24/10/2012 | MN952706 | MN950514 | Yes |
| RMNH.MOL.342327 | Lbul_AMT22_25_15 | Atlantic | AMT22_25 | 20.40 | -38.62 | 24/10/2012 |  |  | Yes |
| RMNH.MOL.342328 | Lbul_AMT22_25_16 | Atlantic | AMT22_25 | 20.40 | -38.62 | 24/10/2012 | MN952707 | MN950741 | Yes |
| RMNH.MOL.342329 | Lbul_AMT22_25_17 | Atlantic | AMT22_25 | 20.40 | -38.62 | 24/10/2012 | MN952708 | MN950515 | Yes |
| RMNH.MOL.342330 | Lbul_AMT22_25_18 | Atlantic | AMT22_25 | 20.40 | -38.62 | 24/10/2012 | MN952709 | MN950516 | Yes |
| RMNH.MOL.342331 | Lbul_AMT22_25_19 | Atlantic | AMT22_25 | 20.40 | -38.62 | 24/10/2012 |  |  | Yes |
| RMNH.MOL.342332 | Lbul_AMT22_25_20 | Atlantic | AMT22_25 | 20.40 | -38.62 | 24/10/2012 |  |  | Yes |
| RMNH.MOL.342333 | Lbul_AMT22_25_21 | Atlantic | AMT22_25 | 20.40 | -38.62 | 24/10/2012 | MN952710 | MN950517 | Yes |
| RMNH.MOL.342334 | Lbul_AMT22_25_22 | Atlantic | AMT22_25 | 20.40 | -38.62 | 24/10/2012 | MN952711 | MN950518 | Yes |
| RMNH.MOL.342335 | Lbul_AMT22_25_23 | Atlantic | AMT22_25 | 20.40 | -38.62 | 24/10/2012 | MN952712 |  | Yes |
| RMNH.MOL.342336 | Lbul_AMT22_25_24 | Atlantic | AMT22_25 | 20.40 | -38.62 | 24/10/2012 |  |  | Yes |
| RMNH.MOL.342337 | Lbul_AMT22_25_25 | Atlantic | AMT22_25 | 20.40 | -38.62 | 24/10/2012 | MN952713 | MN950519 | Yes |
| RMNH.MOL.342338 | Lbul_AMT22_25_26 | Atlantic | AMT22_25 | 20.40 | -38.62 | 24/10/2012 | MN952714 | MN950520 | Yes |
| RMNH.MOL.342339 | Lbul_AMT22_25_27 | Atlantic | AMT22_25 | 20.40 | -38.62 | 24/10/2012 | MN952715 | MN950521 | Yes |
| RMNH.MOL.342340 | Lbul_AMT22_25_28 | Atlantic | AMT22_25 | 20.40 | -38.62 | 24/10/2012 | MN952716 | MN950522 | Yes |
| RMNH.MOL.342341 | Lbul_AMT22_29A_01 | Atlantic | AMT22_29A | 15.30 | -34.67 | 26/10/2012 | MN952717 | MN950523 | Yes |
| RMNH.MOL.342342 | Lbul_AMT22_29A_02 | Atlantic | AMT22_29A | 15.30 | -34.67 | 26/10/2012 |  |  | Yes |
| RMNH.MOL.342343 | Lbul_AMT22_29A_03 | Atlantic | AMT22_29A | 15.30 | -34.67 | 26/10/2012 | MN952718 | MN950524 |  |
| RMNH.MOL.342344 | Lbul_AMT22_29A_04 | Atlantic | AMT22_29A | 15.30 | -34.67 | 26/10/2012 | MN952719 | MN950525 |  |
| RMNH.MOL.342345 | Lbul_AMT22_29A_05 | Atlantic | AMT22_29A | 15.30 | -34.67 | 26/10/2012 | MN952720 | MN950526 | Yes |
| RMNH.MOL.342346 | Lbul_AMT22_29A_06 | Atlantic | AMT22_29A | 15.30 | -34.67 | 26/10/2012 | MN952721 | MN950527 |  |
| RMNH.MOL.342347 | Lbul_AMT22_29A_07 | Atlantic | AMT22_29A | 15.30 | -34.67 | 26/10/2012 | MN952722 | MN950528 | Yes |
| RMNH.MOL.342348 | Lbul_AMT22_29A_08 | Atlantic | AMT22_29A | 15.30 | -34.67 | 26/10/2012 | MN952723 | MN950529 |  |
| RMNH.MOL.342349 | Lbul_AMT22_29A_09 | Atlantic | AMT22_29A | 15.30 | -34.67 | 26/10/2012 | MN952724 | MN950530 |  |
| RMNH.MOL.342350 | Lbul_AMT22_29A_10 | Atlantic | AMT22_29A | 15.30 | -34.67 | 26/10/2012 | MN952725 | MN950531 |  |
| RMNH.MOL.342351 | Lbul_AMT22_29A_11 | Atlantic | AMT22_29A | 15.30 | -34.67 | 26/10/2012 | MN952726 | MN950532 |  |
| RMNH.MOL.342352 | Lbul_AMT22_29A_14 | Atlantic | AMT22_29A | 15.30 | -34.67 | 26/10/2012 | MN952727 | MN950533 |  |
| RMNH.MOL.342353 | Lbul_AMT22_29A_15 | Atlantic | AMT22_29A | 15.30 | -34.67 | 26/10/2012 | MN952728 | MN950534 |  |
| RMNH.MOL.342354 | Lbul_AMT22_29A_17 | Atlantic | AMT22_29A | 15.30 | -34.67 | 26/10/2012 | MN952729 | MN950535 |  |
| RMNH.MOL.342355 | Lbul_AMT22_29A_19 | Atlantic | AMT22_29A | 15.30 | -34.67 | 26/10/2012 | MN952730 | MN950536 |  |
| RMNH.MOL.342356 | Lbul_AMT22_29A_21 | Atlantic | AMT22_29A | 15.30 | -34.67 | 26/10/2012 | MN952731 |  |  |
| RMNH.MOL.342357 | Lbul_AMT22_29A_22 | Atlantic | AMT22_29A | 15.30 | -34.67 | 26/10/2012 | MN952732 | MN950537 |  |
| RMNH.MOL.342358 | Lbul_AMT22_29A_23 | Atlantic | AMT22_29A | 15.30 | -34.67 | 26/10/2012 | MN952733 | MN950538 |  |
| RMNH.MOL.342359 | Lbul_AMT22_29A_24 | Atlantic | AMT22_29A | 15.30 | -34.67 | 26/10/2012 | MN952734 |  |  |
| RMNH.MOL.342360 | Lbul_AMT22_29A_25 | Atlantic | AMT22_29A | 15.30 | -34.67 | 26/10/2012 |  |  | Yes |
| RMNH.MOL.342361 | Lbul_AMT22_29A_26 | Atlantic | AMT22_29A | 15.30 | -34.67 | 26/10/2012 |  |  | Yes |
| RMNH.MOL.342362 | Lbul_AMT22_29A_27 | Atlantic | AMT22_29A | 15.30 | -34.67 | 26/10/2012 |  |  | Yes |
| RMNH.MOL.342363 | Lbul_AMT22_29A_28 | Atlantic | AMT22_29A | 15.30 | -34.67 | 26/10/2012 |  |  | Yes |
| RMNH.MOL.342364 | Lbul_AMT22_29A_29 | Atlantic | AMT22_29A | 15.30 | -34.67 | 26/10/2012 |  |  | Yes |
| RMNH.MOL.342365 | Lbul_AMT22_29A_30 | Atlantic | AMT22_29A | 15.30 | -34.67 | 26/10/2012 | MN952735 | MN950539 | Yes |
| RMNH.MOL.342366 | Lbul_AMT22_29A_31 | Atlantic | AMT22_29A | 15.30 | -34.67 | 26/10/2012 |  |  | Yes |
| RMNH.MOL.342367 | Lbul_AMT22_29A_32 | Atlantic | AMT22_29A | 15.30 | -34.67 | 26/10/2012 |  |  | Yes |
| RMNH.MOL.342368 | Lbul_AMT22_29A_33 | Atlantic | AMT22_29A | 15.30 | -34.67 | 26/10/2012 |  |  | Yes |
| RMNH.MOL.342369 | Lbul_AMT22_29A_34 | Atlantic | AMT22_29A | 15.30 | -34.67 | 26/10/2012 |  |  | Yes |
| RMNH.MOL.342370 | Lbul_AMT22_29A_35 | Atlantic | AMT22_29A | 15.30 | -34.67 | 26/10/2012 |  |  | Yes |
| RMNH.MOL.342371 | Lbul_AMT22_29A_36 | Atlantic | AMT22_29A | 15.30 | -34.67 | 26/10/2012 |  |  | Yes |
| RMNH.MOL.342372 | Lbul_AMT22_29A_37 | Atlantic | AMT22_29A | 15.30 | -34.67 | 26/10/2012 | MN952736 | MN950540 | Yes |
| RMNH.MOL.342373 | Lbul_AMT22_29A_38 | Atlantic | AMT22_29A | 15.30 | -34.67 | 26/10/2012 | MN952737 | MN950541 | Yes |
| RMNH.MOL.342374 | Lbul_AMT22_29A_39 | Atlantic | AMT22_29A | 15.30 | -34.67 | 26/10/2012 |  |  | Yes |
| RMNH.MOL.342375 | Lbul_AMT22_29A_40 | Atlantic | AMT22_29A | 15.30 | -34.67 | 26/10/2012 |  |  | Yes |
| RMNH.MOL.342376 | Lbul_AMT22_43A_01 | Atlantic | AMT22_43A | -4.32 | -25.02 | 1/11/2012 | MN952738 | MN950542 |  |
| RMNH.MOL.342377 | Lbul_AMT22_43A_03 | Atlantic | AMT22_43A | -4.32 | -25.02 | 1/11/2012 | MN952739 | MN950543 |  |
| RMNH.MOL.342378 | Lbul_AMT22_43A_04 | Atlantic | AMT22_43A | -4.32 | -25.02 | 1/11/2012 | MN952740 | MN950544 |  |
| RMNH.MOL.342379 | Lbul_AMT22_43A_05 | Atlantic | AMT22_43A | -4.32 | -25.02 | 1/11/2012 | MN952741 |  |  |
| RMNH.MOL.342380 | Lbul_AMT22_43A_06 | Atlantic | AMT22_43A | -4.32 | -25.02 | 1/11/2012 | MN952742 | MN950545 |  |
| RMNH.MOL.342381 | Lbul_AMT22_43A_08 | Atlantic | AMT22_43A | -4.32 | -25.02 | 1/11/2012 | MN952743 | MN950546 |  |
| RMNH.MOL.342382 | Lbul_AMT22_43A_11 | Atlantic | AMT22_43A | -4.32 | -25.02 | 1/11/2012 | MN952744 | MN950547 |  |
| RMNH.MOL.342383 | Lbul_AMT22_43A_13 | Atlantic | AMT22_43A | -4.32 | -25.02 | 1/11/2012 | MN952745 | MN950548 |  |
| RMNH.MOL.342384 | Lbul_AMT22_43A_20 | Atlantic | AMT22_43A | -4.32 | -25.02 | 1/11/2012 | MN952746 |  |  |
| RMNH.MOL.342385 | Lbul_AMT22_43A_21 | Atlantic | AMT22_43A | -4.32 | -25.02 | 1/11/2012 | MN952747 | MN950549 |  |
| RMNH.MOL.342386 | Lbul_AMT22_43A_22 | Atlantic | AMT22_43A | -4.32 | -25.02 | 1/11/2012 | MN952748 | MN950550 |  |
| RMNH.MOL.342387 | Lbul_AMT22_43A_24 | Atlantic | AMT22_43A | -4.32 | -25.02 | 1/11/2012 | MN952749 |  |  |
| RMNH.MOL.342388 | Lbul_AMT22_43A_25 | Atlantic | AMT22_43A | -4.32 | -25.02 | 1/11/2012 | MN952750 | MN950551 |  |
| RMNH.MOL.342389 | Lbul_AMT22_43A_26 | Atlantic | AMT22_43A | -4.32 | -25.02 | 1/11/2012 | MN952751 | MN950552 |  |
| RMNH.MOL.342390 | Lbul_AMT22_43A_27 | Atlantic | AMT22_43A | -4.32 | -25.02 | 1/11/2012 |  |  | Yes |
| RMNH.MOL.342391 | Lbul_AMT22_43A_28 | Atlantic | AMT22_43A | -4.32 | -25.02 | 1/11/2012 | MN952752 |  |  |
| RMNH.MOL.342392 | Lbul_AMT22_43A_29 | Atlantic | AMT22_43A | -4.32 | -25.02 | 1/11/2012 | MN952753 |  |  |
| RMNH.MOL.342393 | Lbul_AMT22_43A_30 | Atlantic | AMT22_43A | -4.32 | -25.02 | 1/11/2012 | MN952754 | MN950553 |  |
| RMNH.MOL.342394 | Lbul_AMT22_43A_31 | Atlantic | AMT22_43A | -4.32 | -25.02 | 1/11/2012 | MN952755 | MN950554 |  |
| RMNH.MOL.342395 | Lbul_AMT22_43A_32 | Atlantic | AMT22_43A | -4.32 | -25.02 | 1/11/2012 | MN952756 | MN950555 |  |
| RMNH.MOL.342396 | Lbul_AMT22_43A_33 | Atlantic | AMT22_43A | -4.32 | -25.02 | 1/11/2012 |  |  | Yes |
| RMNH.MOL.342397 | Lbul_AMT22_43A_34 | Atlantic | AMT22_43A | -4.32 | -25.02 | 1/11/2012 | MN952757 | MN950556 | Yes |
| RMNH.MOL.342398 | Lbul_AMT22_43A_35 | Atlantic | AMT22_43A | -4.32 | -25.02 | 1/11/2012 | MN952758 | MN950742 |  |
| RMNH.MOL.342399 | Lbul_AMT22_43A_36 | Atlantic | AMT22_43A | -4.32 | -25.02 | 1/11/2012 | MN952759 | MN950557 |  |
| RMNH.MOL.342400 | Lbul_AMT22_43A_37 | Atlantic | AMT22_43A | -4.32 | -25.02 | 1/11/2012 | MN952760 | MN950558 |  |
| RMNH.MOL.342401 | Lbul_AMT22_43A_38 | Atlantic | AMT22_43A | -4.32 | -25.02 | 1/11/2012 | MN952761 | MN950743 |  |
| RMNH.MOL.342402 | Lbul_AMT22_43A_39 | Atlantic | AMT22_43A | -4.32 | -25.02 | 1/11/2012 | MN952762 | MN950744 |  |
| RMNH.MOL.342403 | Lbul_AMT22_45_01 | Atlantic | AMT22_45 | -8.08 | -25.03 | 3/11/2012 | MN952763 | MN950559 |  |
| RMNH.MOL.342404 | Lbul_AMT22_45_02 | Atlantic | AMT22_45 | -8.08 | -25.03 | 3/11/2012 | MN952764 | MN950560 |  |
| RMNH.MOL.342405 | Lbul_AMT22_45_03 | Atlantic | AMT22_45 | -8.08 | -25.03 | 3/11/2012 | MN952765 | MN950561 |  |
| RMNH.MOL.342406 | Lbul_AMT22_45_04 | Atlantic | AMT22_45 | -8.08 | -25.03 | 3/11/2012 | MN952766 | MN950562 |  |
| RMNH.MOL.342407 | Lbul_AMT22_45_05 | Atlantic | AMT22_45 | -8.08 | -25.03 | 3/11/2012 | MN952767 | MN950563 |  |
| RMNH.MOL.342408 | Lbul_AMT22_45_06 | Atlantic | AMT22_45 | -8.08 | -25.03 | 3/11/2012 | MN952768 | MN950564 |  |
| RMNH.MOL.342409 | Lbul_AMT22_45_08 | Atlantic | AMT22_45 | -8.08 | -25.03 | 3/11/2012 | MN952769 | MN950565 |  |
| RMNH.MOL.342410 | Lbul_AMT22_45_09 | Atlantic | AMT22_45 | -8.08 | -25.03 | 3/11/2012 | MN952770 | MN950566 |  |
| RMNH.MOL.342411 | Lbul_AMT22_45_10 | Atlantic | AMT22_45 | -8.08 | -25.03 | 3/11/2012 | MN952771 | MN950567 |  |
| RMNH.MOL.342412 | Lbul_AMT22_45_11 | Atlantic | AMT22_45 | -8.08 | -25.03 | 3/11/2012 | MN952772 | MN950568 |  |
| RMNH.MOL.342413 | Lbul_AMT22_45_12 | Atlantic | AMT22_45 | -8.08 | -25.03 | 3/11/2012 | MN952773 | MN950569 |  |
| RMNH.MOL.342414 | Lbul_AMT22_45_13 | Atlantic | AMT22_45 | -8.08 | -25.03 | 3/11/2012 | MN952774 | MN950570 |  |
| RMNH.MOL.342415 | Lbul_AMT22_45_14 | Atlantic | AMT22_45 | -8.08 | -25.03 | 3/11/2012 | MN952775 | MN950571 |  |
| RMNH.MOL.342416 | Lbul_AMT22_45_15 | Atlantic | AMT22_45 | -8.08 | -25.03 | 3/11/2012 | MN952776 | MN950572 |  |
| RMNH.MOL.342417 | Lbul_AMT22_45_16 | Atlantic | AMT22_45 | -8.08 | -25.03 | 3/11/2012 | MN952777 | MN950573 |  |
| RMNH.MOL.342418 | Lbul_AMT22_45_17 | Atlantic | AMT22_45 | -8.08 | -25.03 | 3/11/2012 | MN952778 | MN950574 |  |
| RMNH.MOL.342419 | Lbul_AMT22_45_18 | Atlantic | AMT22_45 | -8.08 | -25.03 | 3/11/2012 | MN952779 | MN950575 |  |
| RMNH.MOL.342420 | Lbul_AMT22_45_19 | Atlantic | AMT22_45 | -8.08 | -25.03 | 3/11/2012 | MN952780 | MN950576 |  |
| RMNH.MOL.342421 | Lbul_AMT22_45_20 | Atlantic | AMT22_45 | -8.08 | -25.03 | 3/11/2012 | MN952781 | MN950577 |  |
| RMNH.MOL.342422 | Lbul_AMT22_45_21 | Atlantic | AMT22_45 | -8.08 | -25.03 | 3/11/2012 | MN952782 | MN950578 |  |
| RMNH.MOL.342423 | Lbul_AMT22_45_22 | Atlantic | AMT22_45 | -8.08 | -25.03 | 3/11/2012 | MN952783 | MN950579 |  |
| RMNH.MOL.342424 | Lbul_AMT22_45_23 | Atlantic | AMT22_45 | -8.08 | -25.03 | 3/11/2012 |  |  | Yes |
| RMNH.MOL.342425 | Lbul_AMT22_45_24 | Atlantic | AMT22_45 | -8.08 | -25.03 | 3/11/2012 | MN952784 | MN950580 | Yes |
| RMNH.MOL.342426 | Lbul_AMT22_45_25 | Atlantic | AMT22_45 | -8.08 | -25.03 | 3/11/2012 | MN952785 | MN950581 |  |
| RMNH.MOL.342427 | Lbul_AMT22_45_26 | Atlantic | AMT22_45 | -8.08 | -25.03 | 3/11/2012 | MN952786 | MN950582 | Yes |
| RMNH.MOL.342428 | Lbul_AMT22_45_27 | Atlantic | AMT22_45 | -8.08 | -25.03 | 3/11/2012 | MN952787 | MN950583 | Yes |
| RMNH.MOL.342429 | Lbul_AMT22_45_28 | Atlantic | AMT22_45 | -8.08 | -25.03 | 3/11/2012 | MN952788 | MN950584 | Yes |
| RMNH.MOL.342430 | Lbul_AMT22_45_29 | Atlantic | AMT22_45 | -8.08 | -25.03 | 3/11/2012 | MN952789 | MN950585 |  |
| RMNH.MOL.342431 | Lbul_AMT22_45_30 | Atlantic | AMT22_45 | -8.08 | -25.03 | 3/11/2012 | MN952790 | MN950586 | Yes |
| RMNH.MOL.342432 | Lbul_AMT22_45_31 | Atlantic | AMT22_45 | -8.08 | -25.03 | 3/11/2012 | MN952791 | MN950587 |  |
| RMNH.MOL.342433 | Lbul_AMT22_45_32 | Atlantic | AMT22_45 | -8.08 | -25.03 | 3/11/2012 | MN952792 |  |  |
| RMNH.MOL.342434 | Lbul_AMT22_45_33 | Atlantic | AMT22_45 | -8.08 | -25.03 | 3/11/2012 | MN952793 | MN950745 |  |
| RMNH.MOL.342435 | Lbul_AMT22_45_34 | Atlantic | AMT22_45 | -8.08 | -25.03 | 3/11/2012 | MN952794 | MN950746 |  |
| RMNH.MOL.342436 | Lbul_AMT22_45_35 | Atlantic | AMT22_45 | -8.08 | -25.03 | 3/11/2012 | MN952795 | MN950747 |  |
| RMNH.MOL.342437 | Lbul_AMT22_45_38 | Atlantic | AMT22_45 | -8.08 | -25.03 | 3/11/2012 |  | MN950748 |  |
| RMNH.MOL.342438 | Lbul_AMT22_45_39 | Atlantic | AMT22_45 | -8.08 | -25.03 | 3/11/2012 | MN952796 | MN950749 |  |
| RMNH.MOL.342439 | Lbul_AMT22_45_40 | Atlantic | AMT22_45 | -8.08 | -25.03 | 3/11/2012 | MN952797 | MN950588 |  |
| RMNH.MOL.342440 | Lbul_AMT22_45_41 | Atlantic | AMT22_45 | -8.08 | -25.03 | 3/11/2012 | MN952798 | MN950589 |  |
| RMNH.MOL.342441 | Lbul_AMT22_45_42 | Atlantic | AMT22_45 | -8.08 | -25.03 | 3/11/2012 | MN952799 | MN950590 |  |
| RMNH.MOL.342442 | Lbul_AMT22_45_43 | Atlantic | AMT22_45 | -8.08 | -25.03 | 3/11/2012 | MN952800 | MN950591 |  |
| RMNH.MOL.342443 | Lbul_AMT22_45_44 | Atlantic | AMT22_45 | -8.08 | -25.03 | 3/11/2012 | MN952801 | MN950592 |  |
| RMNH.MOL.342444 | Lbul_AMT22_45_45 | Atlantic | AMT22_45 | -8.08 | -25.03 | 3/11/2012 | MN952802 | MN950593 |  |
| RMNH.MOL.342445 | Lbul_AMT22_45_46 | Atlantic | AMT22_45 | -8.08 | -25.03 | 3/11/2012 | MN952803 | MN950594 |  |
| RMNH.MOL.342446 | Lbul_AMT22_49_01 | Atlantic | AMT22_49 | -15.30 | -25.07 | 5/11/2012 | MN952804 | MN950595 |  |
| RMNH.MOL.342447 | Lbul_AMT22_49_04 | Atlantic | AMT22_49 | -15.30 | -25.07 | 5/11/2012 | MN952805 |  |  |
| RMNH.MOL.342448 | Lbul_AMT22_49_05 | Atlantic | AMT22_49 | -15.30 | -25.07 | 5/11/2012 | MN952806 | MN950596 |  |
| RMNH.MOL.342449 | Lbul_AMT22_49_06 | Atlantic | AMT22_49 | -15.30 | -25.07 | 5/11/2012 | MN952807 | MN950597 |  |
| RMNH.MOL.342450 | Lbul_AMT22_49_07 | Atlantic | AMT22_49 | -15.30 | -25.07 | 5/11/2012 | MN952808 | MN950598 |  |
| RMNH.MOL.342451 | Lbul_AMT22_49_08 | Atlantic | AMT22_49 | -15.30 | -25.07 | 5/11/2012 | MN952809 | MN950599 |  |
| RMNH.MOL.342452 | Lbul_AMT22_49_09 | Atlantic | AMT22_49 | -15.30 | -25.07 | 5/11/2012 | MN952810 | MN950600 |  |
| RMNH.MOL.342453 | Lbul_AMT22_49_10 | Atlantic | AMT22_49 | -15.30 | -25.07 | 5/11/2012 | MN952811 | MN950601 |  |
| RMNH.MOL.342454 | Lbul_AMT22_49_11 | Atlantic | AMT22_49 | -15.30 | -25.07 | 5/11/2012 | MN952812 | MN950602 |  |
| RMNH.MOL.342455 | Lbul_AMT22_49_12 | Atlantic | AMT22_49 | -15.30 | -25.07 | 5/11/2012 | MN952813 | MN950603 |  |
| RMNH.MOL.342456 | Lbul_AMT22_49_13 | Atlantic | AMT22_49 | -15.30 | -25.07 | 5/11/2012 | MN952814 | MN950604 |  |
| RMNH.MOL.342457 | Lbul_AMT22_49_14 | Atlantic | AMT22_49 | -15.30 | -25.07 | 5/11/2012 | MN952815 | MN950605 |  |
| RMNH.MOL.342458 | Lbul_AMT22_49_15 | Atlantic | AMT22_49 | -15.30 | -25.07 | 5/11/2012 | MN952816 | MN950606 |  |
| RMNH.MOL.342459 | Lbul_AMT22_49_16 | Atlantic | AMT22_49 | -15.30 | -25.07 | 5/11/2012 | MN952817 | MN950607 |  |
| RMNH.MOL.342460 | Lbul_AMT22_49_17 | Atlantic | AMT22_49 | -15.30 | -25.07 | 5/11/2012 | MN952818 | MN950608 |  |
| RMNH.MOL.342461 | Lbul_AMT22_49_18 | Atlantic | AMT22_49 | -15.30 | -25.07 | 5/11/2012 | MN952819 | MN950609 |  |
| RMNH.MOL.342462 | Lbul_AMT22_49_19 | Atlantic | AMT22_49 | -15.30 | -25.07 | 5/11/2012 | MN952820 | MN950610 |  |
| RMNH.MOL.342463 | Lbul_AMT22_49_20 | Atlantic | AMT22_49 | -15.30 | -25.07 | 5/11/2012 | MN952821 | MN950611 |  |
| RMNH.MOL.342464 | Lbul_AMT22_49_21 | Atlantic | AMT22_49 | -15.30 | -25.07 | 5/11/2012 | MN952822 | MN950612 |  |
| RMNH.MOL.342465 | Lbul_AMT22_49_22 | Atlantic | AMT22_49 | -15.30 | -25.07 | 5/11/2012 | MN952823 | MN950613 |  |
| RMNH.MOL.342466 | Lbul_AMT22_49_23 | Atlantic | AMT22_49 | -15.30 | -25.07 | 5/11/2012 | MN952824 | MN950614 |  |
| RMNH.MOL.342467 | Lbul_AMT22_49_24 | Atlantic | AMT22_49 | -15.30 | -25.07 | 5/11/2012 | MN952825 | MN950615 |  |
| RMNH.MOL.342468 | Lbul_AMT22_49_25 | Atlantic | AMT22_49 | -15.30 | -25.07 | 5/11/2012 | MN952826 | MN950616 |  |
| RMNH.MOL.342469 | Lbul_AMT22_49_26 | Atlantic | AMT22_49 | -15.30 | -25.07 | 5/11/2012 | MN952827 | MN950617 |  |
| RMNH.MOL.342470 | Lbul_AMT22_49_28 | Atlantic | AMT22_49 | -15.30 | -25.07 | 5/11/2012 | MN952828 | MN950750 |  |
| RMNH.MOL.342471 | Lbul_AMT22_49_29 | Atlantic | AMT22_49 | -15.30 | -25.07 | 5/11/2012 |  | MN950751 |  |
| RMNH.MOL.342472 | Lbul_AMT22_49_30 | Atlantic | AMT22_49 | -15.30 | -25.07 | 5/11/2012 |  | MN950752 |  |
| RMNH.MOL.342473 | Lbul_AMT22_49_31 | Atlantic | AMT22_49 | -15.30 | -25.07 | 5/11/2012 | MN952829 | MN950753 |  |
| RMNH.MOL.342474 | Lbul_AMT22_49_33 | Atlantic | AMT22_49 | -15.30 | -25.07 | 5/11/2012 |  | MN950754 |  |
| RMNH.MOL.342475 | Lbul_AMT22_49_34 | Atlantic | AMT22_49 | -15.30 | -25.07 | 5/11/2012 |  | MN950755 |  |
| RMNH.MOL.340293 | Lbul_AMT22_49_35 | Atlantic | AMT22_49 | -15.30 | -25.07 | 5/11/2012 |  | MK635470 |  |
| RMNH.MOL.342477 | Lbul_AMT22_49_36 | Atlantic | AMT22_49 | -15.30 | -25.07 | 5/11/2012 | MN952830 | MN950618 |  |
| RMNH.MOL.342478 | Lbul_AMT22_51_02 | Atlantic | AMT22_51 | -18.50 | -25.10 | 6/11/2012 | MN952831 | MN950619 |  |
| RMNH.MOL.342479 | Lbul_AMT22_51_03 | Atlantic | AMT22_51 | -18.50 | -25.10 | 6/11/2012 | MN952832 | MN950620 |  |
| RMNH.MOL.342480 | Lbul_AMT22_51_04 | Atlantic | AMT22_51 | -18.50 | -25.10 | 6/11/2012 | MN952833 | MN950621 |  |
| RMNH.MOL.342481 | Lbul_AMT22_51_06 | Atlantic | AMT22_51 | -18.50 | -25.10 | 6/11/2012 | MN952834 | MN950622 |  |
| RMNH.MOL.342482 | Lbul_AMT22_51_11 | Atlantic | AMT22_51 | -18.50 | -25.10 | 6/11/2012 | MN952835 | MN950623 | Yes |
| RMNH.MOL.342483 | Lbul_AMT22_51_12 | Atlantic | AMT22_51 | -18.50 | -25.10 | 6/11/2012 | MN952836 | MN950624 |  |
| RMNH.MOL.342484 | Lbul_AMT22_51_13 | Atlantic | AMT22_51 | -18.50 | -25.10 | 6/11/2012 | MN952837 | MN950625 |  |
| RMNH.MOL.342485 | Lbul_AMT22_51_14 | Atlantic | AMT22_51 | -18.50 | -25.10 | 6/11/2012 | MN952838 | MN950626 |  |
| RMNH.MOL.342486 | Lbul_AMT22_51_15 | Atlantic | AMT22_51 | -18.50 | -25.10 | 6/11/2012 | MN952839 | MN950627 |  |
| RMNH.MOL.342487 | Lbul_AMT22_51_16 | Atlantic | AMT22_51 | -18.50 | -25.10 | 6/11/2012 | MN952840 | MN950628 |  |
| RMNH.MOL.342488 | Lbul_AMT22_51_18 | Atlantic | AMT22_51 | -18.50 | -25.10 | 6/11/2012 | MN952841 | MN950629 |  |
| RMNH.MOL.342489 | Lbul_AMT22_51_19 | Atlantic | AMT22_51 | -18.50 | -25.10 | 6/11/2012 | MN952842 | MN950630 |  |
| RMNH.MOL.342490 | Lbul_AMT22_51_20 | Atlantic | AMT22_51 | -18.50 | -25.10 | 6/11/2012 | MN952843 | MN950631 |  |
| RMNH.MOL.342491 | Lbul_AMT22_51_21 | Atlantic | AMT22_51 | -18.50 | -25.10 | 6/11/2012 | MN952844 | MN950632 |  |
| RMNH.MOL.342492 | Lbul_AMT22_51_22 | Atlantic | AMT22_51 | -18.50 | -25.10 | 6/11/2012 | MN952845 | MN950633 |  |
| RMNH.MOL.342493 | Lbul_AMT22_51_24 | Atlantic | AMT22_51 | -18.50 | -25.10 | 6/11/2012 | MN952846 | MN950634 |  |
| RMNH.MOL.342494 | Lbul_AMT22_53_01 | Atlantic | AMT22_53 | -20.10 | -24.52 | 6/11/2012 | MN952847 | MN950635 | Yes |
| RMNH.MOL.342495 | Lbul_AMT22_53_02 | Atlantic | AMT22_53 | -20.10 | -24.52 | 6/11/2012 | MN952848 | MN950636 | Yes |
| RMNH.MOL.342496 | Lbul_AMT22_53_03 | Atlantic | AMT22_53 | -20.10 | -24.52 | 6/11/2012 | MN952849 | MN950637 | Yes |
| RMNH.MOL.342497 | Lbul_AMT22_53_04 | Atlantic | AMT22_53 | -20.10 | -24.52 | 6/11/2012 |  |  | Yes |
| RMNH.MOL.342498 | Lbul_AMT22_53_05 | Atlantic | AMT22_53 | -20.10 | -24.52 | 6/11/2012 | MN952850 | MN950638 | Yes |
| RMNH.MOL.342499 | Lbul_AMT22_53_07 | Atlantic | AMT22_53 | -20.10 | -24.52 | 6/11/2012 | MN952851 | MN950639 | Yes |
| RMNH.MOL.342500 | Lbul_AMT22_53_08 | Atlantic | AMT22_53 | -20.10 | -24.52 | 6/11/2012 | MN952852 | MN950640 | Yes |
| RMNH.MOL.342501 | Lbul_AMT22_53_09 | Atlantic | AMT22_53 | -20.10 | -24.52 | 6/11/2012 | MN952853 | MN950641 |  |
| RMNH.MOL.342502 | Lbul_AMT22_53_10 | Atlantic | AMT22_53 | -20.10 | -24.52 | 6/11/2012 | MN952854 | MN950642 | Yes |
| RMNH.MOL.342503 | Lbul_AMT22_53_11 | Atlantic | AMT22_53 | -20.10 | -24.52 | 6/11/2012 |  |  | Yes |
| RMNH.MOL.342504 | Lbul_AMT22_53_12 | Atlantic | AMT22_53 | -20.10 | -24.52 | 6/11/2012 |  |  | Yes |
| RMNH.MOL.342505 | Lbul_AMT22_53_13 | Atlantic | AMT22_53 | -20.10 | -24.52 | 6/11/2012 | MN952855 | MN950643 |  |
| RMNH.MOL.342506 | Lbul_AMT22_53_14 | Atlantic | AMT22_53 | -20.10 | -24.52 | 6/11/2012 | MN952856 | MN950644 | Yes |
| RMNH.MOL.342507 | Lbul_AMT22_53_15 | Atlantic | AMT22_53 | -20.10 | -24.52 | 6/11/2012 | MN952857 | MN950645 | Yes |
| RMNH.MOL.342508 | Lbul_AMT22_53_16 | Atlantic | AMT22_53 | -20.10 | -24.52 | 6/11/2012 | MN952858 | MN950646 |  |
| RMNH.MOL.342509 | Lbul_AMT22_53_17 | Atlantic | AMT22_53 | -20.10 | -24.52 | 6/11/2012 | MN952859 | MN950647 | Yes |
| RMNH.MOL.342510 | Lbul_AMT22_53_18 | Atlantic | AMT22_53 | -20.10 | -24.52 | 6/11/2012 |  |  | Yes |
| RMNH.MOL.342511 | Lbul_AMT22_53_20 | Atlantic | AMT22_53 | -20.10 | -24.52 | 6/11/2012 | MN952860 | MN950648 | Yes |
| RMNH.MOL.342512 | Lbul_AMT22_53_21 | Atlantic | AMT22_53 | -20.10 | -24.52 | 6/11/2012 | MN952861 | MN950649 | Yes |
| RMNH.MOL.342513 | Lbul_AMT22_53_22 | Atlantic | AMT22_53 | -20.10 | -24.52 | 6/11/2012 | MN952862 | MN950650 |  |
| RMNH.MOL.342514 | Lbul_AMT22_53_39 | Atlantic | AMT22_53 | -20.10 | -24.52 | 6/11/2012 | MN952863 | MN950651 |  |
| RMNH.MOL.342515 | Lbul_AMT22_53_40 | Atlantic | AMT22_53 | -20.10 | -24.52 | 6/11/2012 | MN952864 | MN950652 | Yes |
| RMNH.MOL.342516 | Lbul_AMT22_53_41 | Atlantic | AMT22_53 | -20.10 | -24.52 | 6/11/2012 | MN952865 | MN950653 |  |
| RMNH.MOL.342517 | Lbul_AMT22_53_42 | Atlantic | AMT22_53 | -20.10 | -24.52 | 6/11/2012 | MN952866 | MN950654 |  |
| RMNH.MOL.342518 | Lbul_AMT22_53_43 | Atlantic | AMT22_53 | -20.10 | -24.52 | 6/11/2012 | MN952867 | MN950655 |  |
| RMNH.MOL.342519 | Lbul_AMT22_53_44 | Atlantic | AMT22_53 | -20.10 | -24.52 | 6/11/2012 | MN952868 | MN950656 |  |
| RMNH.MOL.342520 | Lbul_AMT22_53_45 | Atlantic | AMT22_53 | -20.10 | -24.52 | 6/11/2012 | MN952869 | MN950657 |  |
| RMNH.MOL.342521 | Lbul_AMT22_53_46 | Atlantic | AMT22_53 | -20.10 | -24.52 | 6/11/2012 | MN952870 | MN950658 |  |
| RMNH.MOL.342522 | Lbul_AMT22_53_48 | Atlantic | AMT22_53 | -20.10 | -24.52 | 6/11/2012 | MN952871 | MN950659 |  |
| RMNH.MOL.342523 | Lbul_AMT22_55_01 | Atlantic | AMT22_55 | -22.95 | -25.00 | 8/11/2012 | MN952872 | MN950660 | Yes |
| RMNH.MOL.342524 | Lbul_AMT22_55_02 | Atlantic | AMT22_55 | -22.95 | -25.00 | 8/11/2012 | MN952873 | MN950661 |  |
| RMNH.MOL.342525 | Lbul_AMT22_55_03 | Atlantic | AMT22_55 | -22.95 | -25.00 | 8/11/2012 | MN952874 | MN950662 |  |
| RMNH.MOL.342526 | Lbul_AMT22_55_04 | Atlantic | AMT22_55 | -22.95 | -25.00 | 8/11/2012 | MN952875 | MN950663 |  |
| RMNH.MOL.342527 | Lbul_AMT22_55_05 | Atlantic | AMT22_55 | -22.95 | -25.00 | 8/11/2012 | MN952876 | MN950664 |  |
| RMNH.MOL.342528 | Lbul_AMT22_55_06 | Atlantic | AMT22_55 | -22.95 | -25.00 | 8/11/2012 | MN952877 | MN950665 | Yes |
| RMNH.MOL.342529 | Lbul_AMT22_55_07 | Atlantic | AMT22_55 | -22.95 | -25.00 | 8/11/2012 | MN952878 | MN950666 |  |
| RMNH.MOL.342530 | Lbul_AMT22_55_08 | Atlantic | AMT22_55 | -22.95 | -25.00 | 8/11/2012 | MN952879 | MN950667 |  |
| RMNH.MOL.342531 | Lbul_AMT22_55_10 | Atlantic | AMT22_55 | -22.95 | -25.00 | 8/11/2012 | MN952880 | MN950668 | Yes |
| RMNH.MOL.342532 | Lbul_AMT22_55_12 | Atlantic | AMT22_55 | -22.95 | -25.00 | 8/11/2012 | MN952881 | MN950669 |  |
| RMNH.MOL.342533 | Lbul_AMT22_55_13 | Atlantic | AMT22_55 | -22.95 | -25.00 | 8/11/2012 | MN952882 | MN950670 |  |
| RMNH.MOL.340292 | Lbul_AMT22_55_14 | Atlantic | AMT22_55 | -22.95 | -25.00 | 8/11/2012 | MK642914 | MN950671 |  |
| RMNH.MOL.342535 | Lbul_AMT22_55_15 | Atlantic | AMT22_55 | -22.95 | -25.00 | 8/11/2012 | MN952883 | MN950672 |  |
| RMNH.MOL.342536 | Lbul_AMT22_55_16 | Atlantic | AMT22_55 | -22.95 | -25.00 | 8/11/2012 | MN952884 | MN950673 |  |
| RMNH.MOL.342537 | Lbul_AMT22_55_17 | Atlantic | AMT22_55 | -22.95 | -25.00 | 8/11/2012 | MN952885 | MN950674 |  |
| RMNH.MOL.342538 | Lbul_AMT22_55_18 | Atlantic | AMT22_55 | -22.95 | -25.00 | 8/11/2012 | MN952886 | MN950675 |  |
| RMNH.MOL.342539 | Lbul_AMT22_55_19 | Atlantic | AMT22_55 | -22.95 | -25.00 | 8/11/2012 | MN952887 | MN950676 |  |
| RMNH.MOL.342540 | Lbul_AMT22_55_20 | Atlantic | AMT22_55 | -22.95 | -25.00 | 8/11/2012 | MN952888 | MN950677 |  |
| RMNH.MOL.342541 | Lbul_AMT22_55_21 | Atlantic | AMT22_55 | -22.95 | -25.00 | 8/11/2012 | MN952889 | MN950678 |  |
| RMNH.MOL.342542 | Lbul_AMT22_55_22 | Atlantic | AMT22_55 | -22.95 | -25.00 | 8/11/2012 | MN952890 | MN950679 |  |
| RMNH.MOL.342543 | Lbul_AMT22_55_23 | Atlantic | AMT22_55 | -22.95 | -25.00 | 8/11/2012 | MN952891 | MN950680 |  |
| RMNH.MOL.342544 | Lbul_AMT22_55_24 | Atlantic | AMT22_55 | -22.95 | -25.00 | 8/11/2012 | MN952892 | MN950681 |  |
| RMNH.MOL.342545 | Lbul_AMT22_55_25 | Atlantic | AMT22_55 | -22.95 | -25.00 | 8/11/2012 | MN952893 | MN950682 | Yes |
| RMNH.MOL.342546 | Lbul_AMT22_55_26 | Atlantic | AMT22_55 | -22.95 | -25.00 | 8/11/2012 | MN952894 | MN950683 | Yes |
| RMNH.MOL.342547 | Lbul_AMT22_55_27 | Atlantic | AMT22_55 | -22.95 | -25.00 | 8/11/2012 | MN952895 | MN950684 |  |
| RMNH.MOL.342548 | Lbul_AMT22_55_28 | Atlantic | AMT22_55 | -22.95 | -25.00 | 8/11/2012 | MN952896 | MN950756 |  |
| RMNH.MOL.342549 | Lbul_AMT22_55_29 | Atlantic | AMT22_55 | -22.95 | -25.00 | 8/11/2012 | MN952897 | MN950757 | Yes |
| RMNH.MOL.342550 | Lbul_AMT22_55_30 | Atlantic | AMT22_55 | -22.95 | -25.00 | 8/11/2012 | MN952898 | MN950685 |  |
| RMNH.MOL.342551 | Lbul_AMT22_55_31 | Atlantic | AMT22_55 | -22.95 | -25.00 | 8/11/2012 | MN952899 | MN950758 |  |
| RMNH.MOL.342552 | Lbul_AMT22_55_32 | Atlantic | AMT22_55 | -22.95 | -25.00 | 8/11/2012 | MN952900 | MN950759 |  |
| RMNH.MOL.342553 | Lbul_AMT22_55_33 | Atlantic | AMT22_55 | -22.95 | -25.00 | 8/11/2012 | MN952901 | MN950760 |  |
| RMNH.MOL.342554 | Lbul_AMT22_55_34 | Atlantic | AMT22_55 | -22.95 | -25.00 | 8/11/2012 | MN952902 | MN950686 |  |
| RMNH.MOL.342555 | Lbul_AMT22_55_36 | Atlantic | AMT22_55 | -22.95 | -25.00 | 8/11/2012 | MN952903 | MN950687 |  |
| RMNH.MOL.342556 | Lbul_AMT22_55_40 | Atlantic | AMT22_55 | -22.95 | -25.00 | 8/11/2012 | MN952904 | MN950688 |  |
| RMNH.MOL.342557 | Lbul_AMT22_55_41 | Atlantic | AMT22_55 | -22.95 | -25.00 | 8/11/2012 | MN952905 | MN950689 |  |
| RMNH.MOL.342558 | Lbul_AMT22_55_42 | Atlantic | AMT22_55 | -22.95 | -25.00 | 8/11/2012 | MN952906 | MN950690 |  |
| RMNH.MOL.342559 | Lbul_AMT22_55_43 | Atlantic | AMT22_55 | -22.95 | -25.00 | 8/11/2012 | MN952907 | MN950691 |  |
| RMNH.MOL.342560 | Lbul_AMT22_55_44 | Atlantic | AMT22_55 | -22.95 | -25.00 | 8/11/2012 | MN952908 | MN950692 |  |
| RMNH.MOL.342561 | Lbul_AMT22_60_01 | Atlantic | AMT22_60 | -30.17 | -27.90 | 12/11/2012 | MN952909 | MN950693 |  |
| RMNH.MOL.342562 | Lbul_AMT22_60_02 | Atlantic | AMT22_60 | -30.17 | -27.90 | 12/11/2012 | MN952910 | MN950694 |  |
| RMNH.MOL.342563 | Lbul_AMT22_60_03 | Atlantic | AMT22_60 | -30.17 | -27.90 | 12/11/2012 |  | MN950761 |  |
| RMNH.MOL.342564 | Lbul_AMT22_60_04 | Atlantic | AMT22_60 | -30.17 | -27.90 | 12/11/2012 | MN952911 | MN950695 |  |
| RMNH.MOL.342565 | Lbul_AMT22_60_05 | Atlantic | AMT22_60 | -30.17 | -27.90 | 12/11/2012 | MN952912 | MN950696 |  |
| RMNH.MOL.342566 | Lbul_AMT22_60_08 | Atlantic | AMT22_60 | -30.17 | -27.90 | 12/11/2012 | MN952913 | MN950697 |  |
| RMNH.MOL.342567 | Lbul_AMT22_60_09 | Atlantic | AMT22_60 | -30.17 | -27.90 | 12/11/2012 | MN952914 | MN950698 |  |
| RMNH.MOL.342568 | Lbul_AMT22_60_10 | Atlantic | AMT22_60 | -30.17 | -27.90 | 12/11/2012 | MN952915 | MN950699 |  |
| RMNH.MOL.342569 | Lbul_AMT22_60_11 | Atlantic | AMT22_60 | -30.17 | -27.90 | 12/11/2012 | MN952916 | MN950700 |  |
| RMNH.MOL.342570 | Lbul_AMT22_60_12 | Atlantic | AMT22_60 | -30.17 | -27.90 | 12/11/2012 | MN952917 | MN950701 |  |
| RMNH.MOL.342571 | Lbul_AMT22_60_14 | Atlantic | AMT22_60 | -30.17 | -27.90 | 12/11/2012 | MN952918 | MN950702 |  |
| RMNH.MOL.342572 | Lbul_AMT22_60_15 | Atlantic | AMT22_60 | -30.17 | -27.90 | 12/11/2012 | MN952919 | MN950703 |  |
| RMNH.MOL.342573 | Lbul_AMT22_60_16 | Atlantic | AMT22_60 | -30.17 | -27.90 | 12/11/2012 | MN952920 | MN950704 |  |
| RMNH.MOL.342574 | Lbul_AMT22_60_17 | Atlantic | AMT22_60 | -30.17 | -27.90 | 12/11/2012 | MN952921 | MN950705 |  |
| RMNH.MOL.342575 | Lbul_AMT22_60_18 | Atlantic | AMT22_60 | -30.17 | -27.90 | 12/11/2012 | MN952922 | MN950706 |  |
| RMNH.MOL.342576 | Lbul_AMT22_60_19 | Atlantic | AMT22_60 | -30.17 | -27.90 | 12/11/2012 | MN952923 | MN950707 |  |
| RMNH.MOL.342577 | Lbul_AMT22_60_20 | Atlantic | AMT22_60 | -30.17 | -27.90 | 12/11/2012 | MN952924 | MN950708 |  |
| RMNH.MOL.342578 | Lbul_AMT22_60_21 | Atlantic | AMT22_60 | -30.17 | -27.90 | 12/11/2012 | MN952925 | MN950709 |  |
| RMNH.MOL.342579 | Lbul_AMT22_60_24 | Atlantic | AMT22_60 | -30.17 | -27.90 | 12/11/2012 | MN952926 | MN950710 |  |
| RMNH.MOL.342580 | Lbul_AMT22_60_25 | Atlantic | AMT22_60 | -30.17 | -27.90 | 12/11/2012 |  |  | Yes |
| RMNH.MOL.342581 | Lbul_AMT22_60_26 | Atlantic | AMT22_60 | -30.17 | -27.90 | 12/11/2012 |  |  | Yes |
| RMNH.MOL.342582 | Lbul_AMT22_60_27 | Atlantic | AMT22_60 | -30.17 | -27.90 | 12/11/2012 |  |  | Yes |
| RMNH.MOL.342583 | Lbul_AMT22_60_28 | Atlantic | AMT22_60 | -30.17 | -27.90 | 12/11/2012 |  |  | Yes |
| RMNH.MOL.342584 | Lbul_AMT22_60_29 | Atlantic | AMT22_60 | -30.17 | -27.90 | 12/11/2012 |  |  | Yes |
| RMNH.MOL.342585 | Lbul_AMT22_60_30 | Atlantic | AMT22_60 | -30.17 | -27.90 | 12/11/2012 | MN952927 | MN950762 | Yes |
| RMNH.MOL.342586 | Lbul_AMT22_60_31 | Atlantic | AMT22_60 | -30.17 | -27.90 | 12/11/2012 |  | MN950763 | Yes |
| RMNH.MOL.342587 | Lbul_AMT22_60_32 | Atlantic | AMT22_60 | -30.17 | -27.90 | 12/11/2012 |  | MN950764 |  |
| RMNH.MOL.342588 | Lbul_AMT22_60_33 | Atlantic | AMT22_60 | -30.17 | -27.90 | 12/11/2012 |  | MN950765 | Yes |
| RMNH.MOL.342589 | Lbul_AMT22_60_34 | Atlantic | AMT22_60 | -30.17 | -27.90 | 12/11/2012 |  | MN950766 |  |
| RMNH.MOL.342590 | Lbul_AMT22_60_36 | Atlantic | AMT22_60 | -30.17 | -27.90 | 12/11/2012 | MN952928 | MN950767 |  |
| RMNH.MOL.342591 | Lbul_AMT22_60_37 | Atlantic | AMT22_60 | -30.17 | -27.90 | 12/11/2012 | MN952929 | MN950768 |  |
| RMNH.MOL.342592 | Lbul_AMT22_60_38 | Atlantic | AMT22_60 | -30.17 | -27.90 | 12/11/2012 | MN952930 | MN950769 |  |
| RMNH.MOL.342593 | Lbul_AMT22_60_40 | Atlantic | AMT22_60 | -30.17 | -27.90 | 12/11/2012 | MN952931 | MN950711 |  |
| RMNH.MOL.342594 | Lbul_AMT22_60_41 | Atlantic | AMT22_60 | -30.17 | -27.90 | 12/11/2012 | MN952932 |  |  |
| RMNH.MOL.342595 | Lbul_AMT22_62_03 | Atlantic | AMT22_62 | -34.12 | -33.50 | 14/11/2012 | MN952933 | MN950712 |  |
| RMNH.MOL.342596 | Lbul_AMT22_62_04 | Atlantic | AMT22_62 | -34.12 | -33.50 | 14/11/2012 | MN952934 | MN950713 |  |
| RMNH.MOL.342597 | Lbul_AMT22_62_08 | Atlantic | AMT22_62 | -34.12 | -33.50 | 14/11/2012 | MN952935 | MN950714 |  |
| RMNH.MOL.342598 | Lbul_AMT22_62_12 | Atlantic | AMT22_62 | -34.12 | -33.50 | 14/11/2012 | MN952936 | MN950770 |  |
| RMNH.MOL.342599 | Lbul_AMT22_62_13 | Atlantic | AMT22_62 | -34.12 | -33.50 | 14/11/2012 | MN952937 |  |  |
| RMNH.MOL.342600 | Lbul_AMT22_62_15 | Atlantic | AMT22_62 | -34.12 | -33.50 | 14/11/2012 | MN952938 | MN950715 |  |
| RMNH.MOL.342601 | Lbul_AMT22_62_16 | Atlantic | AMT22_62 | -34.12 | -33.50 | 14/11/2012 | MN952939 | MN950716 |  |
| RMNH.MOL.342602 | Lbul_AMT22_62_25 | Atlantic | AMT22_62 | -34.12 | -33.50 | 14/11/2012 | MN952940 | MN950717 |  |
| RMNH.MOL.342603 | Lbul_AMT22_64A_03 | Atlantic | AMT22_64A | -35.87 | -36.00 | 14/11/2012 | MN952941 | MN950718 |  |
| RMNH.MOL.342604 | Lbul_AMT22_64A_07 | Atlantic | AMT22_64A | -35.87 | -36.00 | 14/11/2012 | MN952942 |  |  |
| RMNH.MOL.342605 | Lbul_AMT22_64A_09 | Atlantic | AMT22_64A | -35.87 | -36.00 | 14/11/2012 | MN952943 | MN950719 |  |
| RMNH.MOL.342606 | Lbul_KH1110_08_01 | Pacific | KH1110_08 | 22.78 | -158.10 | 19/12/2011 | MN952944 | MN950771 | Yes |
| RMNH.MOL.342607 | Lbul_KH1110_08_02 | Pacific | KH1110_08 | 22.78 | -158.10 | 19/12/2011 | MN952945 |  | Yes |
| RMNH.MOL.342608 | Lbul_KH1110_08_03 | Pacific | KH1110_08 | 22.78 | -158.10 | 19/12/2011 | MN952946 | MN950772 | Yes |
| RMNH.MOL.342609 | Lbul_KH1110_08_04 | Pacific | KH1110_08 | 22.78 | -158.10 | 19/12/2011 | MN952947 | MN950720 | Yes |
| RMNH.MOL.342610 | Lbul_KH1110_08_05 | Pacific | KH1110_08 | 22.78 | -158.10 | 19/12/2011 | MN952948 | MN950773 | Yes |
| RMNH.MOL.342611 | Lbul_KH1110_08_06 | Pacific | KH1110_08 | 22.78 | -158.10 | 19/12/2011 | MN952949 | MN950774 | Yes |
| RMNH.MOL.342612 | Lbul_KH1110_08_07 | Pacific | KH1110_08 | 22.78 | -158.10 | 19/12/2011 | MN952950 |  | Yes |
| RMNH.MOL.342613 | Lbul_KH1110_08_08 | Pacific | KH1110_08 | 22.78 | -158.10 | 19/12/2011 | MN952951 | MN950775 | Yes |
| RMNH.MOL.342614 | Lbul_KH1110_08_09 | Pacific | KH1110_08 | 22.78 | -158.10 | 19/12/2011 | MN952952 | MN950721 | Yes |
| RMNH.MOL.342615 | Lbul_KH1110_08_10 | Pacific | KH1110_08 | 22.78 | -158.10 | 19/12/2011 |  |  | Yes |
| RMNH.MOL.342616 | Lbul_KH1110_08_11 | Pacific | KH1110_08 | 22.78 | -158.10 | 19/12/2011 | MN952953 | MN950722 | Yes |
| RMNH.MOL.342617 | Lbul_KH1110_08_12 | Pacific | KH1110_08 | 22.78 | -158.10 | 19/12/2011 | MN952954 | MN950723 | Yes |
| RMNH.MOL.342618 | Lbul_KH1110_08_13 | Pacific | KH1110_08 | 22.78 | -158.10 | 19/12/2011 | MN952955 | MN950724 | Yes |
| RMNH.MOL.342619 | Lbul_KH1110_08_14 | Pacific | KH1110_08 | 22.78 | -158.10 | 19/12/2011 |  |  | Yes |
| RMNH.MOL.342620 | Lbul_KH1110_08_15 | Pacific | KH1110_08 | 22.78 | -158.10 | 19/12/2011 | MN952956 | MN950725 | Yes |
| RMNH.MOL.342621 | Lbul_KH1110_08_16 | Pacific | KH1110_08 | 22.78 | -158.10 | 19/12/2011 | MN952957 | MN950726 | Yes |
| RMNH.MOL.342622 | Lbul_KH1110_08_17 | Pacific | KH1110_08 | 22.78 | -158.10 | 19/12/2011 | MN952958 | MN950727 | Yes |
| RMNH.MOL.342623 | Lbul_KH1110_08_18 | Pacific | KH1110_08 | 22.78 | -158.10 | 19/12/2011 | MN952959 | MN950728 | Yes |
| RMNH.MOL.342624 | Lbul_KH1110_08_19 | Pacific | KH1110_08 | 22.78 | -158.10 | 19/12/2011 | MN952960 | MN950729 | Yes |
| RMNH.MOL.342625 | Lbul_KH1110_08_20 | Pacific | KH1110_08 | 22.78 | -158.10 | 19/12/2011 | MN952961 | MN950730 | Yes |
| RMNH.MOL.342626 | Lbul_KH1110_08_21 | Pacific | KH1110_08 | 22.78 | -158.10 | 19/12/2011 |  |  | Yes |
| RMNH.MOL.342627 | Lbul_KH1110_08_22 | Pacific | KH1110_08 | 22.78 | -158.10 | 19/12/2011 | MN952962 | MN950731 | Yes |
| RMNH.MOL.342628 | Lbul_KH1110_08_23 | Pacific | KH1110_08 | 22.78 | -158.10 | 19/12/2011 | MN952963 | MN950732 | Yes |
| RMNH.MOL.342629 | Lbul_KH1110_08_24 | Pacific | KH1110_08 | 22.78 | -158.10 | 19/12/2011 | MN952964 | MN950733 | Yes |
| RMNH.MOL.342630 | Lbul_KH1110_08_25 | Pacific | KH1110_08 | 22.78 | -158.10 | 19/12/2011 | MN952965 | MN950734 | Yes |

**Table S2** Mantel tests for pairwise Φ_ST_ and geographic distance across the entire Atlantic transect as well as different population groups. Significant Φ_ST_ values after Bonferroni correction (α = 0.05, p < 0.005) are in bold.

| **Group tested** | **COI** | | **28S** | |
| --- | --- | --- | --- | --- |
|  | **r** | **p** | **r** | **p** |
| Atlantic | 0.52 | **0.0012** | -0.0942 | 0.77 |
| North + Equatorial | 0.117 | 0.269 | 0.759 | **0.003** |
| South | 0.0968 | 0.418 | 0.732 | 0.0494 |
| North | - | - | 0.359 | 0.202 |
| Equatorial + South | - | - | 0.428 | 0.0317 |
| North + South | - | - | 0.339 | 0.0301 |
| Equatorial | - | - | -0.814 | 0.832 |

**Table S3** Details of 13 expatriate individuals of *Limacina bulimoides* based on mitochondrial cytochrome *c* oxidase I (COI) haplogroup (see Fig. 2 and 3), including information of nuclear 28S haplotype and life stage (n.a. is not available). See also Fig. S4.

| **Expatriate** | **Site** | **COI**  **haplogroup** | **28S**  **haplotype** | **Life stage** |
| --- | --- | --- | --- | --- |
| Lbul_AMT22_19_44 | 19 (North) | 2 | most common | juvenile |
| Lbul_AMT22_25_07 | 25 (North) | 2 | one step away from most common | juvenile |
| Lbul_AMT22_49_01 | 49 (Eq.) | 2 | most common | adult |
| Lbul_AMT22_49_05 | 49 (Eq.) | 2 | most common | n.a. |
| Lbul_AMT22_49_06 | 49 (Eq.) | 2 | most common | n.a. |
| Lbul_AMT22_49_36 | 49 (Eq.) | 2 | most common | juvenile |
| Lbul_AMT22_51_14 | 51 (South) | 1 | most common | juvenile |
| Lbul_AMT22_53_01 | 53 (South) | 1 | most common | adult |
| Lbul_AMT22_53_17 | 53 (South) | 1 | most common | adult |
| Lbul_AMT22_55_08 | 55 (South) | 1 | most common | n.a. |
| Lbul_AMT22_55_36 | 55 (South) | 1 | most common | juvenile |
| Lbul_AMT22_55_41 | 55 (South) | 1 | most common | juvenile |
| Lbul_AMT22_64A_07 | 64 (South) | 1 | n.a. | n.a. |

**Table S4** Repeatability analysis (N = 30 *Limacina bulimoides* individuals x 2 images each) by comparison of centroid size and relative warps (RW) through intraclass correlation coefficient (ICC). Repeatable parameters, defined as having an ICC of more than 0.75, are labelled in bold.

| **Parameter** | **ICC** | **95% CI** | **Percentage explained (%)** |
| --- | --- | --- | --- |
| **Centroid** | 0.9983 | 0.9962 - 0.9992 | - |
| **RW1** | 0.9948 | 0.9891 - 0.9975 | 81.99 |
| **RW2** | 0.8581 | 0.7247 - 0.9297 | 5.3 |
| RW3 | 0.6626 | 0.3994 - 0.8243 | 4.17 |
| **RW4** | 0.8537 | 0.7126 - 0.928 | 3.22 |
| RW5 | 0.6917 | 0.4435 - 0.8408 | 2.26 |
| **RW6** | 0.8276 | 0.6715 - 0.9138 | 0.86 |
| **RW7** | 0.7906 | 0.6075 - 0.8942 | 0.71 |

**Table S5** Results of ANOVA and Tukey HSD multiple pairwise comparisons of Canonical Variate 1 (see Fig S2) across the three Atlantic population groups of *Limacina bulimoides* (North, Equatorial and South). Significant comparisons are indicated in bold.

| **Group** | **n** | **Mean** | **SD** | **Tukey HSD comparisons** | | |
| --- | --- | --- | --- | --- | --- | --- |
|  |  |  |  | **North** | **Equatorial** | **South** |
| North | 70 | -3.19102 | 0.913883 | - | - | - |
| Equatorial | 9 | -3.35496 | 1.255741 | 0.88871 | - | - |
| South | 32 | -1.59519 | 1.103622 | **0.00000** | **0.00003** | - |
